# Supplementary material for: Highly efficient pure-blue organic light-emitting diodes based on rationally designed heterocyclic phenophosphazinine-containing emitters
Source: Nat Commun. 2024 Jul 22;15:6175. doi: 10.1038/s41467-024-50370-5 (PMC11263564; doi:10.1038/s41467-024-50370-5)
Supplement: Supplementary file 1 — Supplementary Information [file 41467_2024_50370_MOESM1_ESM.pdf]

Supplementary Information

Highly efficient pure-blue organic light-emitting diodes based on rationally designed heterocyclic phenophosphazinine-containing emitters

Longjiang Xing[1], Jianghui Wang[2], Wen-Cheng Chen[1,3]*, Bo Liu[1], Guowei Chen[1], Xiaofeng Wang[1], Ji-Hua Tan,[1] Season Si Chen,[4] Jia-Xiong Chen[1,3], Shaomin Ji[1,3], Zujin Zhao[2,]*, Man-Chung Tang[4,]* and Yanping Huo[1,3,5]*

[1]School of Chemical Engineering and Light Industry, Guangdong University of Technology Guangzhou 510006, P. R. China

[2]State Key Laboratory of Luminescent Materials and Devices Key Laboratory of Luminescence from Molecular Aggregates of Guangdong Province South China University of Technology Guangzhou 510640, P. R. China

[3]Guangdong Provincial Laboratory of Chemistry and Fine Chemical Engineering Jieyang Center, Jieyang, 515200, P. R. China

[4]Institute of Materials Research, Tsinghua Shenzhen International Graduate School, Tsinghua University, Shenzhen, 518055, P. R. China

[5]Analytical & Testing Center Guangdong University of Technology Guangzhou 510006, P. R. China

These authors contributed equally: Longjiang Xing, Jianghui Wang

*E-mail: wencchen@gdut.edu.cn; mszjzhao@scut.edu.cn; kobetang2021@sz.tsinghua.edu.cn; yphuo@gdut.edu.cn

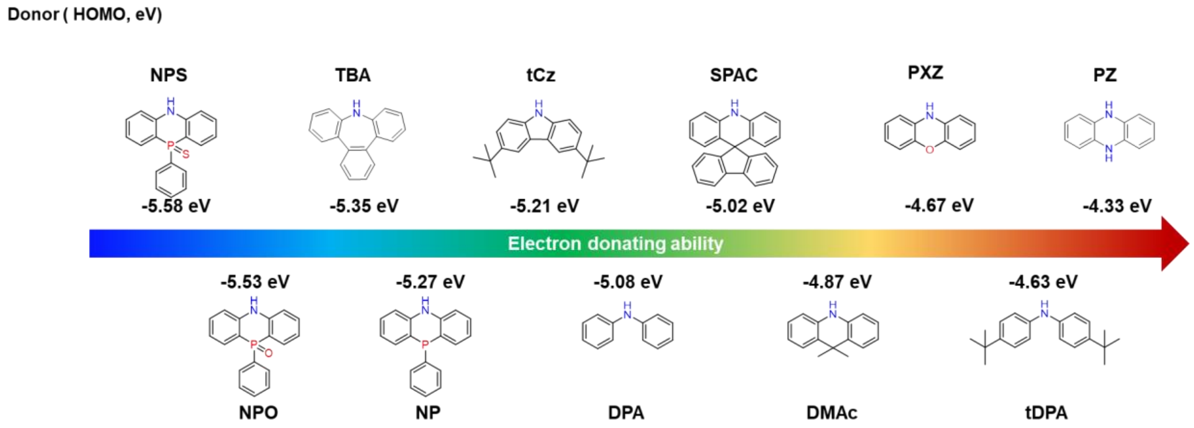

Supplementary Fig. 1 | Molecule structure and HOMO energy levels analysis. Structure and HOMO energy levels of commonly used electron donors and NPO/NPS computed by DFT at the B3LYP/6-31G (d) level.

Supplementary Methods

1. Quantum chemical calculations

All calculations were performed with Gaussian 16_A03 program package.[1] The ground-state geometries were optimized using the density function theory (DFT) method with B3LYP functional at the basis set level of 6-31G (d). Time-dependent DFT (TD-DFT) method were performed at the B3LYP/def2-TZVP level to obtain the NTOs of the singlet and triplet states. The energy levels for singlets and triplets were calculated by SCS-CC2 calculations using the MRCC Program with cc-pVDZ basis set.[2,3] The root-mean squared displacement (RMSD) between S_0 and S_1 molecular geometries was calculated and visualized with the VMD program.[4] NTOs analysis was conducted in Multiwfn 3.7 code.[5] Reorganization energy analysis was carried out using Molecular Materials Property Prediction Package (MOMAP). Based on the optimized S_0 molecular geometries, the spin-orbit coupling matrix elements (SOCEMs) between two first singlet and triplet excited states were calculated in B3LYP/G TZVP level based on the T_1 relaxed geometry using Orca 4.1.2 package.

2. Analysis of rate constants

The rate constants of radiative decay ($k_{r,S}$) and nonradiative decay ($k_{nr,S}$) from S_1 to S_0 states, the rate constants of intersystem crossing (k_{ISC}) and reverse intersystem crossing (k_{RISC}) were calculated from the following equations[6]:

$$k_p = 1/\tau_p \quad (1)$$

$$k_d = 1/\tau_d \quad (2)$$

$$k_{r,S} = \Phi_p k_p + \Phi_p k_d \approx \Phi_p k_p \quad (3)$$

$k_{nr,S} = \frac{1 - \Phi_{PL}}{\Phi_{PL}} k_{r,s}$ .... (4)

$k_{ISC} = k_p - k_{r,s} - k_{nr,s}$ .... (5)

$k_{RISC} = (k_p k_d \Phi_d)/(k_{ISC}\Phi_p)$.. (6)

Where τ_p and τ_d represent the prompt and decay fluorescence lifetime, which determined from transient PL spectra. The k_p and k_d represent the decay rate constants for prompt and delayed fluorescence, respectively. Φ_p and Φ_d indicate prompt and delayed fluorescence components and can be distinguished from the total Φ_{PL} by comparing the integrated intensities of prompt and delayed components in the transient PL spectra.

3. Materials and Synthesis

Commercially available reagents and solvents were used as received unless otherwise noted.

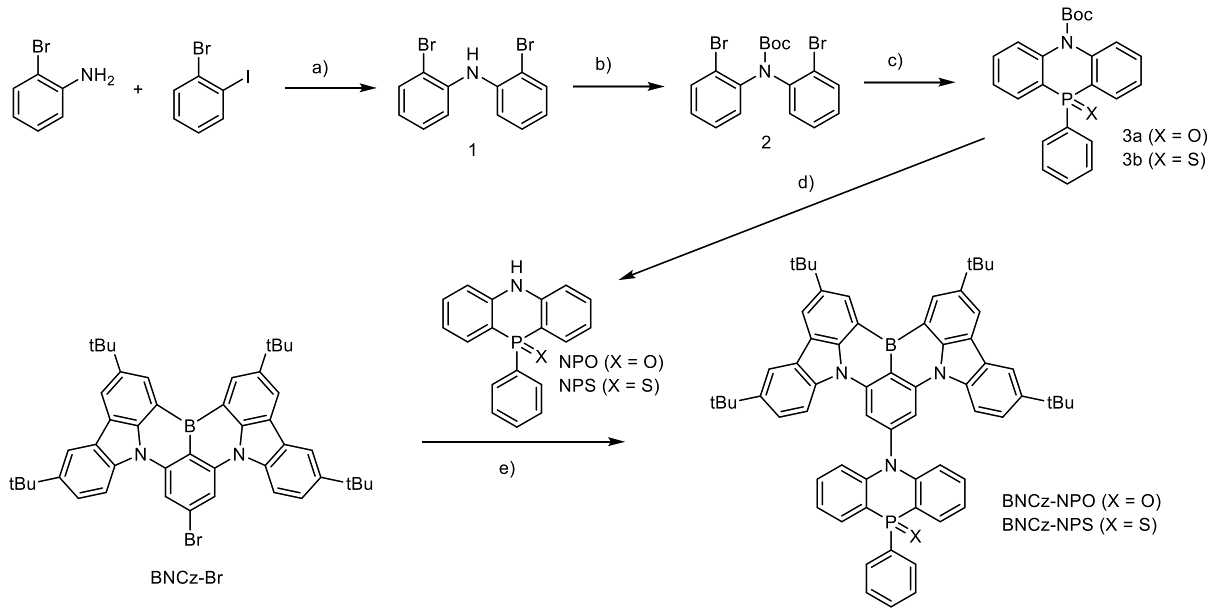

Supplementary Fig. 2 | The synthetic routes of BNCz-NPO and BNCz-NPS. a) $Pd_2(dba)_3$, dppf, *t*-BuONa, toluene, 120 °C, 12 h; b) DMAP, Boc_2O, THF, 80 °C, 12 h; c) *n*-BuLi, THF, -78ºC, 1 h; dichloro(phenyl)phosphane, -78ºC, 1 h； 30% H_2O_2 (for 3a) at room temperature, CH_2Cl_2； or S_8 (for 3b) at 110 ºC, 8 h； d) CF_3COOH, CH_2Cl_2, rt, 12 h; e) $Pd_2(dba)_3$, *t*-BuONa, *t*-Bu_3PHBF_4, toluene, 120 ºC, 12 h.

Synthesis of bis(2-bromophenyl)amine (1). A mixture of 2-bromoaniline (1.72 g, 10 mmol), 1-bromo-2-iodobenzene (4.24 g, 15mmol), Sodium *tert*-butoxide (2.88 g, 30 mmol), 1,1'-bis(diphenylphosphino)ferrocene (0.554 g, 1 mmol) 40 mL toluene and $Pd_2(dba)_3$ (0.45 g, 0.50 mmol) were added and refluxed at 120 °C under nitrogen atmosphere for 12 hours. The mixture

was poured into water to quench the reaction and then extracted with dichloromethane. The organic phase was collected and dried over anhydrous Na_2SO_4. After evaporation of the solvent, the residue was purified via column chromatography using petroleum ether/dichloromethane (10:1, v/v) as an eluent to afford a white solid. (2.59 g, yield: 80%).^{1}H NMR (400 MHz, DMSO$_{d6}$) δ (ppm): 7.64 (dd, J = 8.0, 1.2 Hz, 2H), 7.36 – 7.24 (m, 2H), 7.07 (dd, J = 8.0, 1.1 Hz, 2H), 7.01 (s, 1H), 6.93 (td, J = 8.0, 1.3 Hz, 2H).

Synthesis of *tert*-butyl bis(2-bromophenyl)carbamate (2). A mixture of bis(2-bromophenyl)amine (3.24 g, 10 mmol), *N*,*N*-dimethyl-4-aminopyridine (0.244 g, 2 mmol) , di-*tert*-butyl dicarbonate (4.36 g, 20 mmol) and 60 ml THF were added and refluxed at 80°C under nitrogen atmosphere for 12 hours. The mixture was poured into water to quench the reaction and then extracted with dichloromethane. The organic phase was collected and dried over anhydrous Na_2SO_4. After evaporation of the solvent, the residue was purified via column chromatography using petroleum ether/dichloromethane (3:1, v/v) as eluent to afford white solid. (3.61g, yield: 85%).^{1}H NMR (400 MHz, $CDCl_3$,) δ (ppm): 7.64 (d, J = 6.7 Hz, 2H), 7.43 (d, J = 7.7 Hz, 2H), 7.22 (td, J = 7.8, 1.3 Hz, 2H), 7.10 (td, J = 7.9, 1.5 Hz, 2H), 1.46 (s, 9H).

Synthesis of *tert*-butyl 10-phenyl-5*H*-phenophosphazinine-5-carboxylate 10-oxide (3a). Step 1: A mixture of 2 (4.24 g, 10 mmol), and 40 ml THF were added three-neck flash. The flash was evacuated, and refilled with nitrogen for 3 times and the mixture was cooled to -78 °C. *n*-BuLi (hexane solution, 2.5 M, 30 mmol, 12 mL) was added dropwise under N_2, and the resulting mixture was stirred for 1 h at -78 °C. Dichloro(phenyl)phosphane (3.55 g, 20 mmol) was added at -78 ºC and then the reaction mixture was allowed to warm to room temperature overnight. The mixture was poured into water to quench the reaction and then extracted with dichloromethane. The organic phase was collected and dried over anhydrous Na_2SO_4. After evaporation of the solvent, the mixture was filtered, and the solid was washed with water, ethanol, and dichloromethane, respectively, to afford a white solid. The crude intermediates were directly used to the next step. Step 2: A mixture of the above intermediate (3.75 g, 10 mmol), 30% hydrogen peroxide (H_2O_2) (20 ml), 30 ml dichloromethane were added and stirred at room temperature under a nitrogen atmosphere for 12 h. The mixture was poured into water to quench the reaction and then extracted with dichloromethane. The organic phase was collected and dried over anhydrous Na_2SO_4. After evaporation of the solvent, the residue was purified via column chromatography by using petroleum ether/ ethyl acetate (1:1, v/v) as an eluent to afford white solid. (1.76 g, yield: 45%). ^{1}H NMR (400 MHz, $CDCl_3$,) δ (ppm): 8.12 (ddd, J = 12.2, 7.5, 1.0 Hz, 2H), 7.67 (dt, J = 13.2, 6.6 Hz, 2H), 7.59 – 7.37 (m, 7H), 7.32 (td, J = 7.5, 3.0 Hz, 2H), 1.28 (s, 9H). ^{31}P NMR (162 MHz, $CDCl_3$) δ (ppm): 16.92 (s).

Synthesis of *tert*-butyl 10-phenyl-5*H*-phenophosphazinine-5-carboxylate 10-sulfide (3b). Step 1: the same as synthetic 3a. Step 2: A mixture of the above intermediate (3.75 g, 10 mmol), 3sulfur (0.97 g, 30 mmol), 30 ml toluene were added and stirred at 110 °C under a nitrogen atmosphere for 12 h. The mixture was poured into water to quench the reaction and then extracted with dichloromethane. The organic phase was collected and dried over anhydrous Na_2SO_4. After evaporation of the solvent, the residue was purified via column chromatography using petroleum ether/ ethyl acetate (8:1, v/v) as eluent to afford white solid. (1.62g, yield: 40%). ^{1}H NMR (400 MHz, DMSO$_{d6}$) δ (ppm): 8.16 (ddd, *J* = 14.8, 7.6, 1.4 Hz, 2H), 7.80 – 7.72 (m, 2H), 7.72 – 7.65 (m, 2H), 7.63 – 7.54 (m, 2H), 7.49 – 7.41 (m, 1H), 7.42 – 7.34 (m, 2H), 7.32 – 7.21 (m, 2H), 1.20 (s, 9H). ^{31}P NMR (162 MHz, DMSO$_{d6}$) δ (ppm): 20.73 (s).

Synthesis of 10-phenyl-5*H*-phenophosphazinine 10-oxide (NPO). A mixture of 3a (1.17 g, 3 mmol), trifluoroacetic acid (6.9 ml, 90 mmol), and 20 ml CH_2Cl_2 were added and refluxed at room temperature under a nitrogen atmosphere for 12 hours. The mixture was poured into water to quench the reaction and then extracted with dichloromethane. The organic phase was collected and dried over anhydrous Na_2SO_4. After evaporation of the solvent, the residue was purified via column chromatography using petroleum ether/ ethyl acetate (1:1, v/v) as an eluent to afford a white solid. (0.59 g, yield: 68%). ^{1}H NMR (400 MHz, DMSO$_{d6}$) δ (ppm): 10.40 (s, 1H), 7.69 – 7.36 (m, 9H), 7.26 (dd, *J* = 8.0, 5.9 Hz, 2H), 7.02 (t, *J* = 7.3 Hz, 2H). ^{31}P NMR (162 MHz, DMSO$_{d6}$) δ (ppm): 4.45 (s).

Synthesis of 10-phenyl-5*H*-phenophosphazinine 10-sulfide (NPS). A mixture of 3b (1.22 g, 3 mmol), trifluoroacetic acid (6.9 ml, 90 mmol), and 20 ml CH_2Cl_2 were added and refluxed at room temperature under a nitrogen atmosphere for 12 h. The mixture was poured into water to quench the reaction and then extracted with dichloromethane. The organic phase was collected and dried over anhydrous Na_2SO_4. After evaporation of the solvent, the residue was purified via column chromatography using petroleum ether/ethyl acetate (8:1, v/v) as an eluent to afford a white solid. (0.60 g, yield: 65%).^{1}H NMR (400 MHz, DMSO$_{d6}$) δ (ppm):10.30 (s, 1H), 7.72 (dd, *J* = 14.9, 7.7 Hz, 2H), 7.48 (dd, *J* = 28.4, 21.8 Hz, 7H), 7.31 – 7.15 (m, 2H), 7.08 (t, *J* = 7.1 Hz, 2H). ^{31}P NMR (162 MHz, DMSO$_{d6}$) δ (ppm): 16.92 (s).

Synthesis of BNCz-NPO. A mixture of NPO (1.45 g, 5 mmol), (*t*-Bu)$_3$P•BF_4(6.9 ml, 0.25 mmol), Sodium *tert*-butoxide (0.98 g, 10 mmol), BNCz-Br[7] (2.15 g, 3 mmol, CAS: 2755996-62-2), 40 mL toluene and $Pd_2(dba)_3$ (0.23 g, 0.50 mmol) were added and refluxed at 120 °C under nitrogen atmosphere for 12 hours. The mixture was poured into water to quench the reaction and then extracted with dichloromethane. The organic phase was collected and dried over anhydrous Na_2SO_4. After evaporation of the solvent, the residue was purified via column

chromatography using petroleum ether/ ethyl acetate (2:1, v/v) as eluent to afford yellow solid. (1.53 g, yield: 55%).^{1}H NMR (400 MHz, $CDCl_3$,) δ (ppm): 8 9.20 (d, *J* = 1.7 Hz, 2H), 8.52 (d, *J* = 1.6 Hz, 2H), 8.31 (s, 2H), 8.26 (d, *J* = 1.9 Hz, 2H), 8.18 (d, *J* = 8.8 Hz, 2H), 7.89 (dddd, *J* = 13.5, 9.1, 6.7, 1.6 Hz,4H), 7.66 – 7.51 (m, 5H), 7.31 (s, 2H), 7.10 (s, 2H), 6.88 (dd, *J* = 8.7, 6.2 Hz, 2H), 1.70 (s, 18H), 1.48 (s, 18H); ^{13}C NMR (101 MHz, $CDCl_3$) δ (ppm): 146.60, 146.19, 145.51, 145.13, 144.92, 144.87, 141.69, 138.07, 135.88, 134.73, 132.85, 132.27, 132.24, 132.22, 132.14, 131.53, 131.51, 130.00, 128.57, 128.44, 127.25, 124.87, 124.04, 121.61, 121.50, 121.25, 117.43, 117.31, 117.24, 114.47, 114.27, 113.44, 109.37, 35.31, 34.85, 32.22, 31.77; ^{31}P NMR (162 MHz, $CDCl_3$) δ (ppm): 5.13 (s). HRMS (ESI) *m*/*z*: $[M + H]^+$ calcd for $C_{64}H_{61}BN_3OP$, 930.4718; found, 930.4717.

Synthesis of BNCz-NPS. BNCz-NPS was synthesized according to the same procedure as for BNCz-NPO by using NPS (1.53 g, 5 mmol) instead of NPO. The residue was purified via column chromatography using petroleum ether/ ethyl acetate (10:1, v/v) as eluent to afford yellow solid. (1.41 g, yield: 50%). ^{1}H NMR (400 MHz, $CDCl_3$,) δ (ppm): 9.09 (d, *J* = 1.4 Hz, 2H), 8.88 (d, *J* = 1.5 Hz, 2H), 8.57 (d, *J* = 1.9 Hz, 2H), 8.34 (s, 2H), 8.16 (d, *J* = 8.9 Hz, 2H), 8.04 (ddd, *J* = 15.7, 7.7, 1.2 Hz, 2H), 7.71 (ddd, *J* = 14.0, 6.5, 3.0 Hz, 2H), 7.66 – 7.55 (m, 5H), 7.42 (t, *J* = 7.8 Hz, 2H), 7.26 (t, *J* = 7.3 Hz, 2H), 6.94 (dd, *J* = 8.4, 6.4 Hz, 2H), 1.66 (s, 18H), 1.45 (s, 18H); ^{13}C NMR (101 MHz, $CDCl_3$) δ (ppm): 146.52, 146.21, 145.57, 144.95, 144.21, 144.18, 141.74, 138.32, 138.12, 137.44, 132.74, 132.66, 130.93, 130.89, 130.82, 130.09, 128.76, 128.64, 127.36, 124.85, 124.07, 123.12, 122.50, 122.38, 121.74, 121.32, 117.54, 116.94, 116.88, 114.19, 113.94, 113.09, 109.55, 35.37, 34.90, 32.29, 31.85; ^{31}P NMR (162 MHz, $CDCl_3$). δ (ppm): 16.89 (s). HRMS (ESI) *m*/*z*: $[M + H]^+$ calcd for $C_{64}H_{61}BN_3PS$, 946.4489; found, 946.4487.

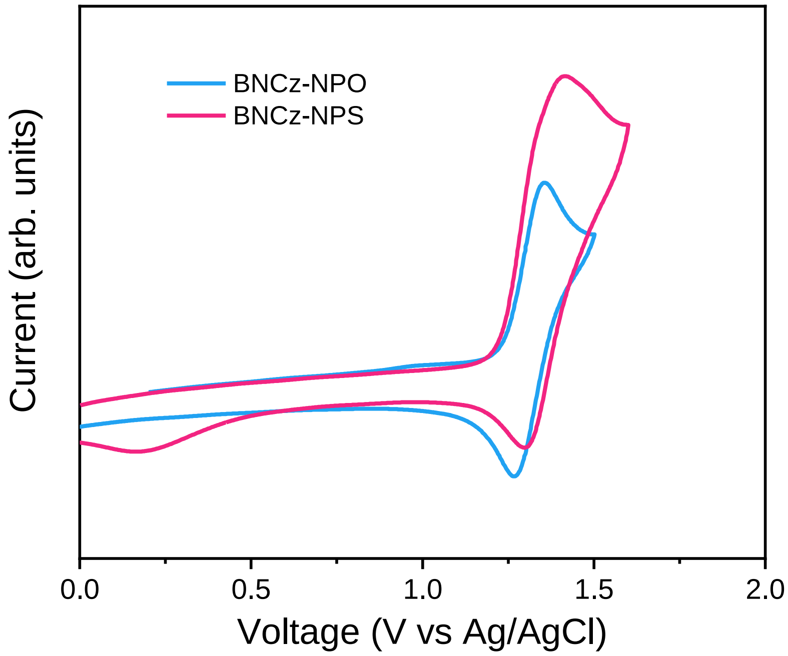

Supplementary Fig. 3 | Electrochemical properties characterization. Cyclic voltammogram of BNCz-NPO and BNCz-NPS.

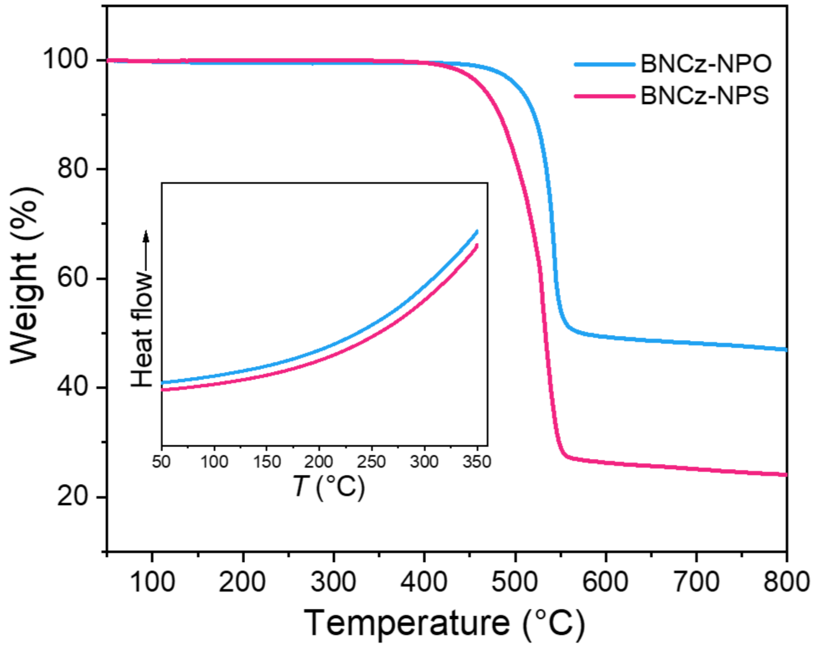

Supplementary Fig. 4 | Thermal properties characterization. TGA and DSC (inset) curves of BNCz-NPO and BNCz-NPS at a heating rate of 10 °C min^{-1}.

Supplementary Table 1 | Crystal data and structure refinement for BNCz-NPO-α and BNCz-NPO-β.

Parameter	BNCz-NPO-α	BNCz-NPO-β
CCDC no.	2253324	2282192
Empirical formula	$C_{64}H_{61}BN_3OP$	$C_{64}H_{61}BN_3OP$
Formula weight (g mol^{-1})	929.93	929.93
Temperature (K)	150.00(10)	150.00(10)
Crystal system	monoclinic	triclinic
Space group	P21/n	P-1
a (Å)	14.4394(2)	12.6376(4)
b (Å)	13.88700(10)	15.0173(3)
c (Å)	26.8466(3)	16.3976(3)
α (°)	90	79.740(2)
Β (°)	102.7370(10)	79.810(2)
γ (°)	90	72.857(2)
Volume (Å^3)	5250.81(10)	2900.44(13)
Z	4	2
Cal. density (g cm^{-3})	1.176	1.065
Absorption coefficient (mm^{-1})	0.803	0.727
F(000)	1976.0	988.0
Crystal size/mm^3	0.13 × 0.12 × 0.08	0.15 × 0.12 × 0.1
Radiation	Cu Kα (λ = 1.54184)	Cu Kα (λ = 1.54184)
2Θ range for data collection (°)	6.438 to 147.732	5.526 to 133.186
Index ranges	-12 ≤ h ≤ 17, -15 ≤ k ≤ 17, -31 ≤ l ≤ 33	-15 ≤ h ≤ 15, -17 ≤ k ≤ 13, -19 ≤ l ≤ 19
Reflections collected	20311	28045
Independent reflections	10387 [R_{int} = 0.0241, R_{sigma} = 0.0311]	10132 [Rint = 0.0375, Rsigma = 0.0453]
Data/restraints/parameters	10387/17/654	10132/57/687
Goodness-of-fit on F^2	1.025	1.108
Final R indexes [I>=2σ (I)]	R_1 = 0.0393, wR_2 = 0.0983	R1 = 0.1100, wR2 = 0.3087
Final R indexes [all data]	R_1 = 0.0439, wR_2 = 0.1020	R1 = 0.1263, wR2 = 0.3187
Largest diff. peak/hole (e Å^{-3})	0.34/-0.32	0.85/-0.47

Supplementary Table 2 | Crystal data and structure refinement for BNCz-NPS-α and BNCz-NPS-β.

Parameter	BNCz-NPS-α	BNCz-NPS-β
CCDC no.	2285218	2247937
Empirical formula	$C_{64}H_{61}BN_3PS$	$C_{64}H_{61}BN_3PS$
Formula weight (g mol^{-1})	945.99	945.99
Temperature (K)	99.98(13)	170.00(10)
Crystal system	monoclinic	monoclinic
Space group	$P2_1/n$	Cc
a (Å)	18.88520(10)	17.4998(9)
b (Å)	25.2745(2)	21.3090(11)
c (Å)	24.1828(2)	17.6514(10)
α (°)	90	90
Β (°)	105.4650(10)	97.258(5)
γ (°)	90	90
Volume (Å^3)	11124.86(15)	6529.5(6)
Z	8	4
Cal. density (g cm^{-3})	1.130	0.962
Absorption coefficient (mm^{-1})	1.093	0.931
F(000)	4016.0	2008.0
Crystal size/mm^3	0.16 × 0.13 × 0.11	0.15 × 0.13 × 0.11
Radiation	Cu Kα (λ = 1.54184)	Cu Kα (λ = 1.54184)
2Θ range for data collection (°)	5.158 to 133.198	7.884 to 133.188
Index ranges	-22 ≤ h ≤ 20, -30 ≤ k ≤ 18, -28 ≤ l ≤ 28	-18 ≤ h ≤ 20, -24 ≤ k ≤ 25, -17 ≤ l ≤ 21
Reflections collected	60705	20416
Independent reflections	19630 [R_{int} = 0.0329, R_{sigma} = 0.0380]	7819 [Rint = 0.0600, Rsigma = 0.0576]
Data/restraints/parameters	19630/1533/1364	7819/854/602
Goodness-of-fit on F^2	1.091	1.091
Final R indexes [I>=2σ (I)]	R_1 = 0.1004, wR_2 = 0.2430	R1 = 0.1062, wR2 = 0.2797
Final R indexes [all data]	R_1 = 0.1053, wR_2 = 0.2461	R1 = 0.1275, wR2 = 0.3026
Largest diff. peak/hole (e Å^{-3})	0.63/-0.68	1.00/-0.80

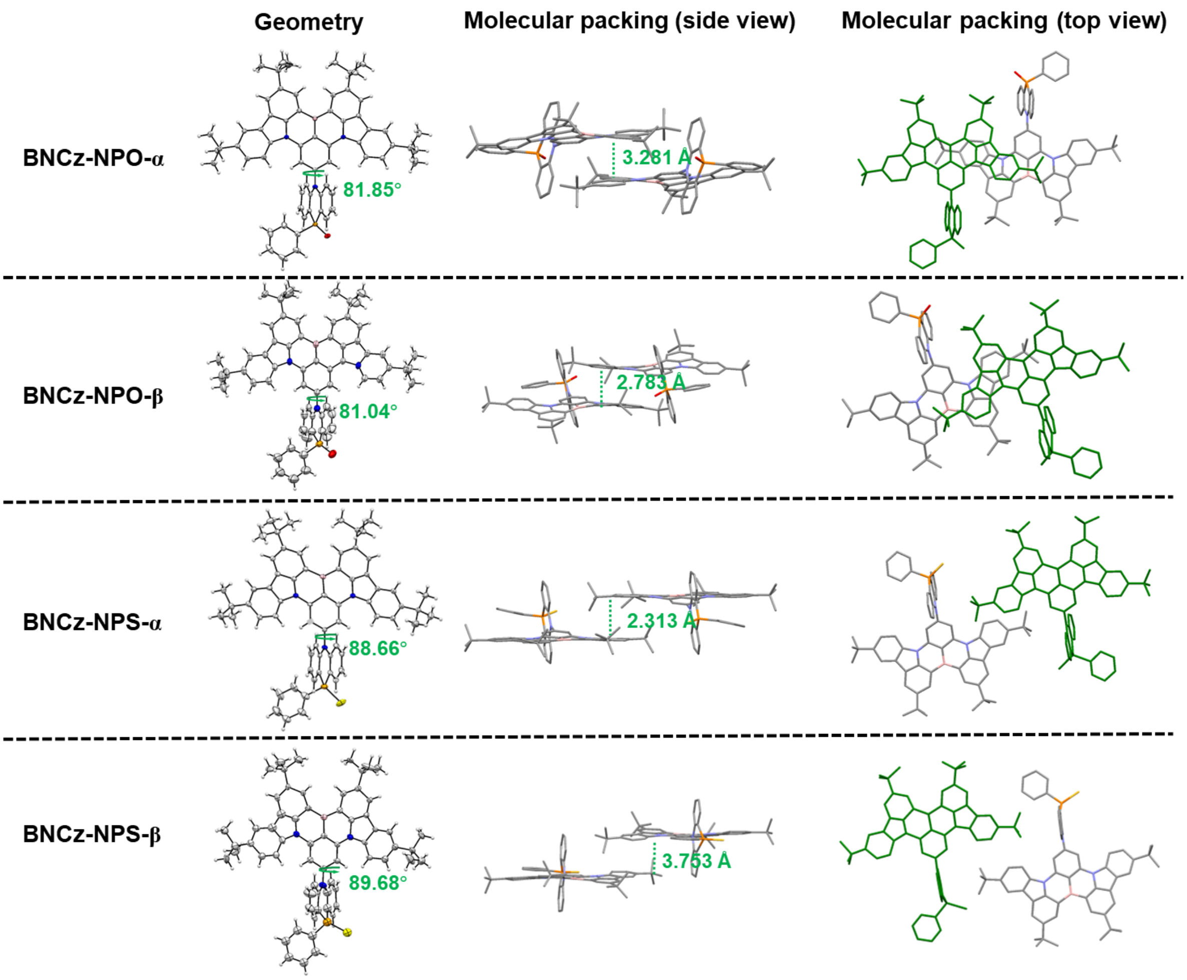

Supplementary Fig. 5 | Single crystal structure analysis. Molecular geometries and packing modes of BNCz-NPO-α (CCDC: 2253324), BNCz-NPO-β (CCDC: 2282192), BNCz-NPS-α (CCDC: 2285218), and BNCz-NPS-β (CCDC: 2247937). Ellipsoids are shown at the 50% probability level.

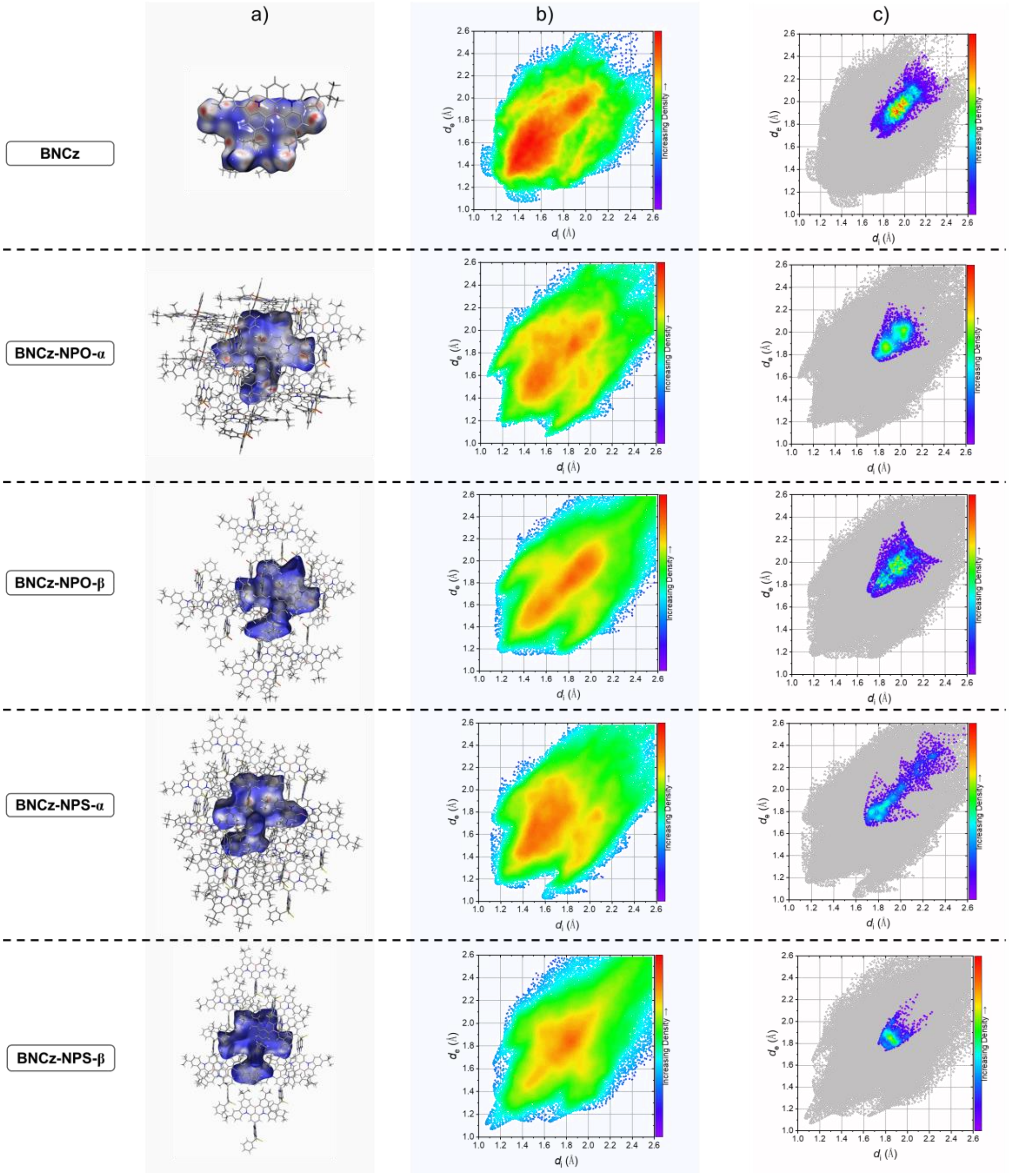

Supplementary Fig. 6 | Hirshfeld surface analysis. a) Hirshfeld surface. The red isosurface portion, indicative of intermolecular contacts. b) Single molecule full fingerprint plot. The full fingerprint plot reveals that BNCz-NPO and BNCz-NPS exhibit sparser contact densities and larger contact distances than BNCz, suggesting weaker intermolecular interactions. c) Decomposed fingerprint plots for specific pairs of the C-C contacts on the MR core. The involvement of planar stacking arrangements for BNCz is evident as a red region (increased density) near the center of the plot (1.8 Å < $d_{i,e}$ < 2.0 Å). In contrast, the interchromophore contact density is greatly reduced in BNCz-NPO and BNCz-NPS, indicating alleviated interchromophore interaction.

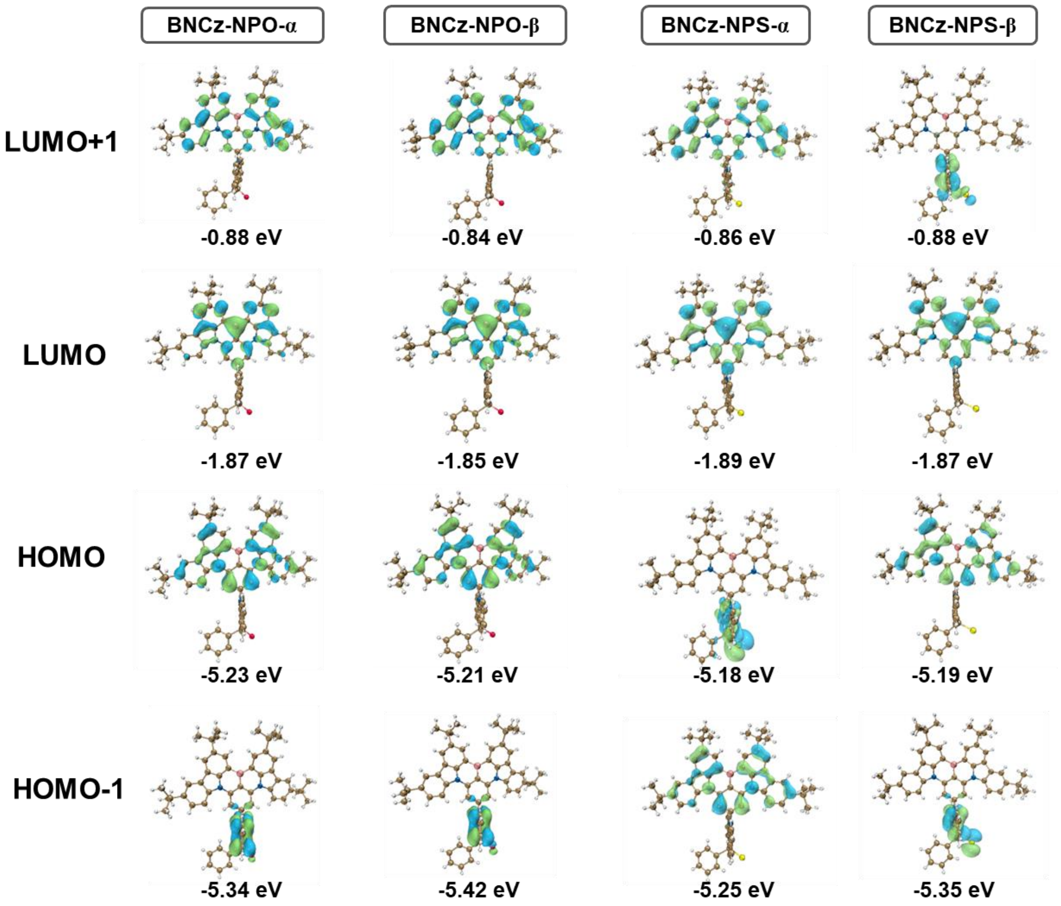

Supplementary Fig. 7 | Molecular orbital analysis. LUMO+1, LUMO, HOMO, HOMO-1 distributions and related energy levels of BNCz-NPO-α, BNCz-NPO-β, BNCz-NPS-α and BNCz-NPS-β.

Supplementary Table 3 | The distance of charge transfer (D_{CT}), and the amount of charge transferred (q_{CT}) in excited states

Compound	S_1		S_2		T_1		T_2		T_3		T_4	
	D_{CT}/Å	q_{CT}	D_{CT}/Å	q_{CT}	D_{CT}/Å	q_{CT}	D_{CT}/Å	q_{CT}	D_{CT}/Å	q_{CT}	D_{CT}/Å	q_{CT}
BNCz	1.04	0.57	0.78	0.54	0.62	0.59	1.15	0.42	1.02	0.42	0.58	0.49
BNCz-NPO	1.15	0.58	3.37	0.93	0.57	0.58	1.76	0.55	1.05	0.47	1.07	0.52
BNCz-NPS	1.18	0.61	3.65	0.95	0.58	0.60	1.44	0.64	1.24	0.53	3.64	0.92

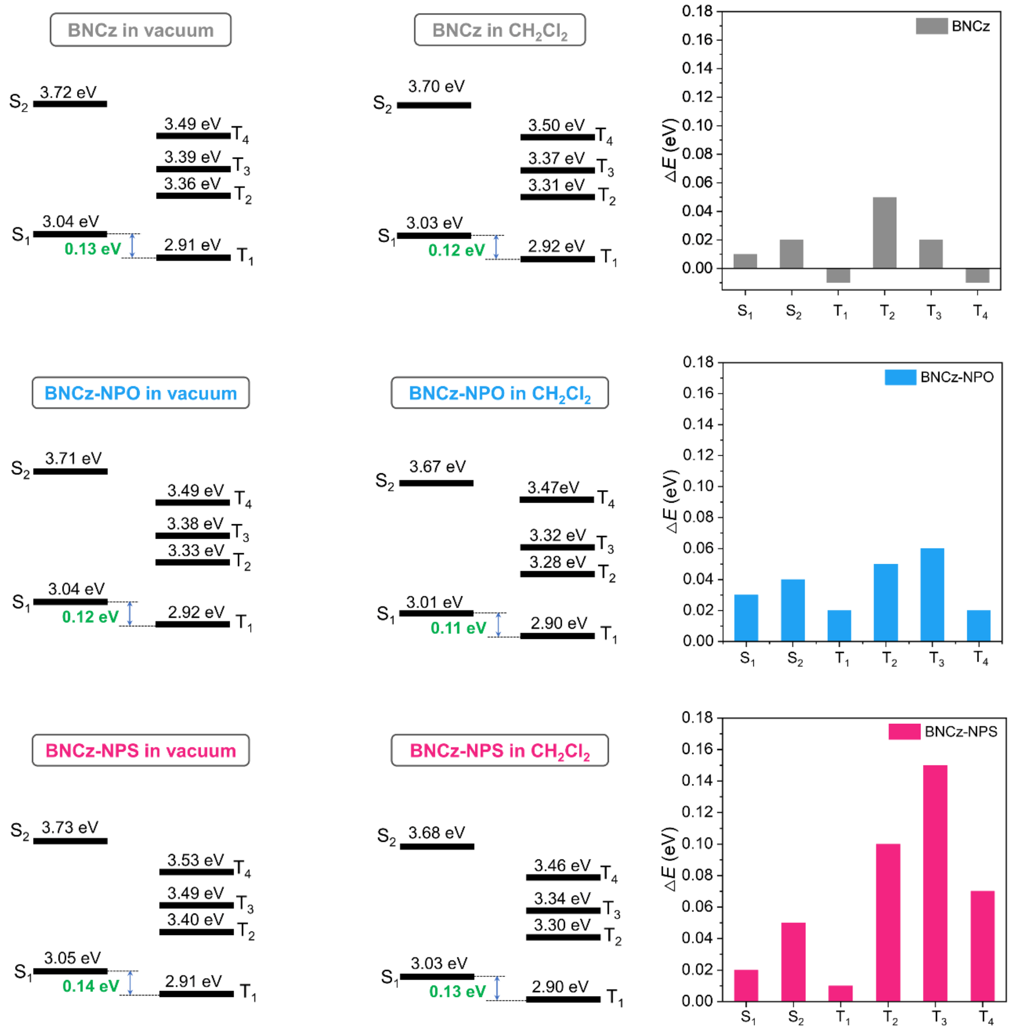

Supplementary Fig. 8 | Energy-level analyses of excited states. Energy-level diagrams for the excited states of BNCz, BNCz-NPO, and BNCz-NPS in both vacuum and CH_2Cl_2 environments. Bar charts are used to illustrate the energy differences between these environments.

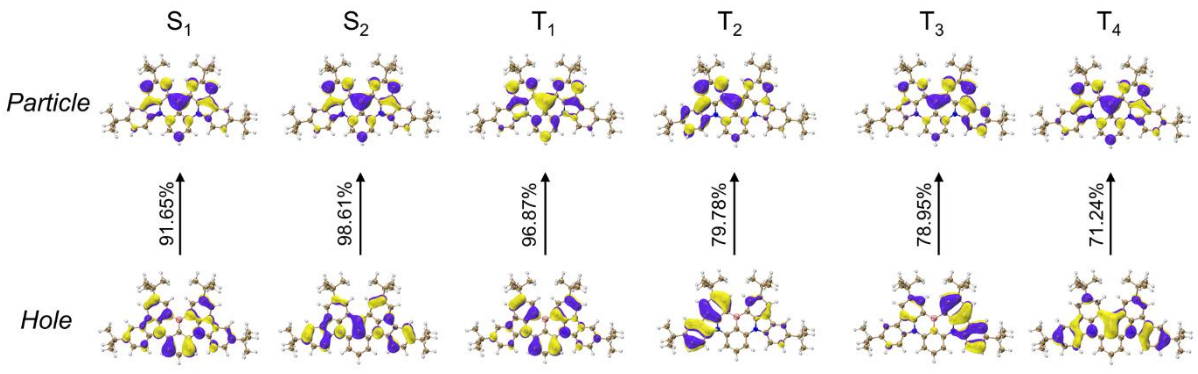

Supplementary Fig. 9 | NTO analysis. Hole-particle distribution for the excited singlet and triplet states of BNCz.

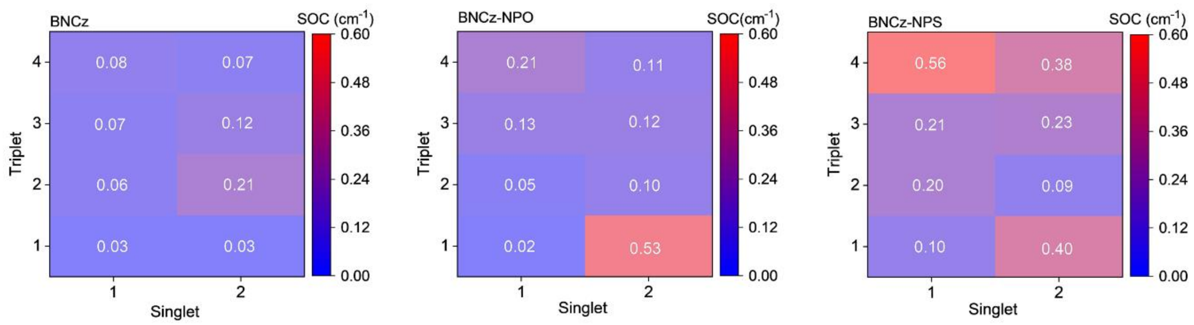

Supplementary Fig. 10 | Analyses of SOC value. SOC heatmaps of BNCz, BNCz-NPO, and BNCz-NPS.

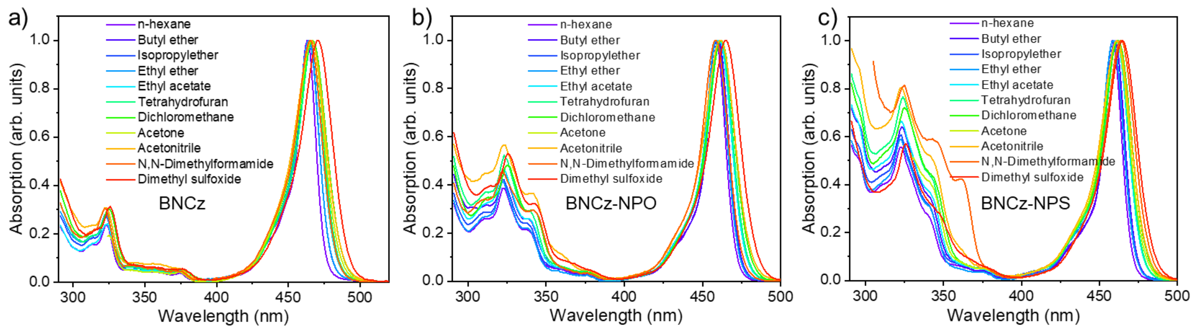

Supplementary Fig. 11 | Solvation effect of absorption. Absorption spectra of a) BNCz, b) BNCz-NPO and c) BNCz-NPS in different solvents (1×10^{-5} M).

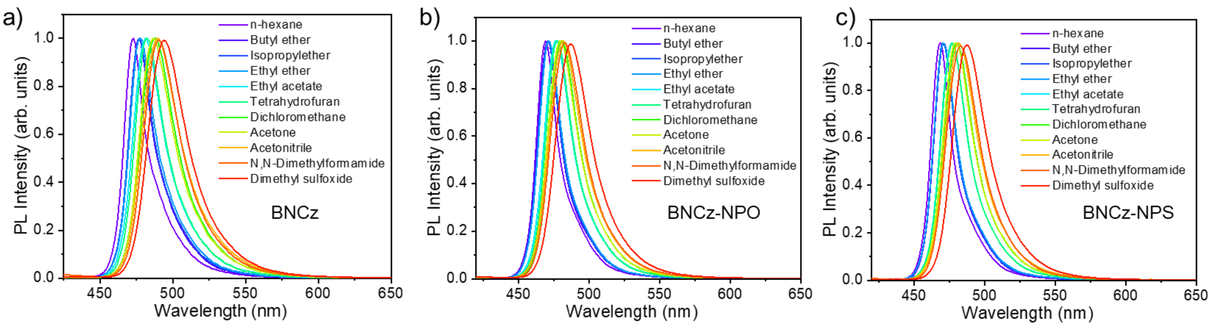

Supplementary Fig. 12 | Solvation effect of photoluminescence. PL spectra of a) BNCz, b) BNCz-NPO and c) BNCz-NPS in different solvents (1×10^{-5} M).

Supplementary Table 4 | Absorption and PL peaks of BNCz, BNCz-NPO and BNCz-NPS measured in different solvents.

	BNCz			BNCz-NPO			BNCz-NPS		
Solvent	λ_{abs}[a] (nm)	λ_{pl}[a] (nm)	FWHM[b] (nm)	λ_{abs}[a] (nm)	λ_{pl}[a] (nm)	FWHM[b] (nm)	λ_{abs}[a] (nm)	λ_{pl}[a] (nm)	FWHM[b] (nm)
n-hexane	463	473	19	458	469	18	458	468	18
Butyl ether	464	477	21	460	471	20	460	471	19
Isopropyl ether	464	477	21	459	471	19	459	470	19
Ethyl ether	464	478	22	459	471	20	459	471	20
Ethyl acetate	465	481	26	460	477	23	461	477	23
Tetrahydrofuran	466	482	25	461	477	23	461	477	23
Dichloromethane	467	488	29	462	481	25	462	480	25
Acetone	466	487	28	460	480	26	461	479	26
Acetonitrile	466	490	31	461	482	27	461	481	27
N,*N*-dimethylformamide	465	490	30	458	483	28	463	483	28
Dimethyl sulfoxide	470	494	32	465	487	30	465	487	29

[a] Peak wavelength of absorption and emission in different solvents; [b] Full-width at half-maximum.

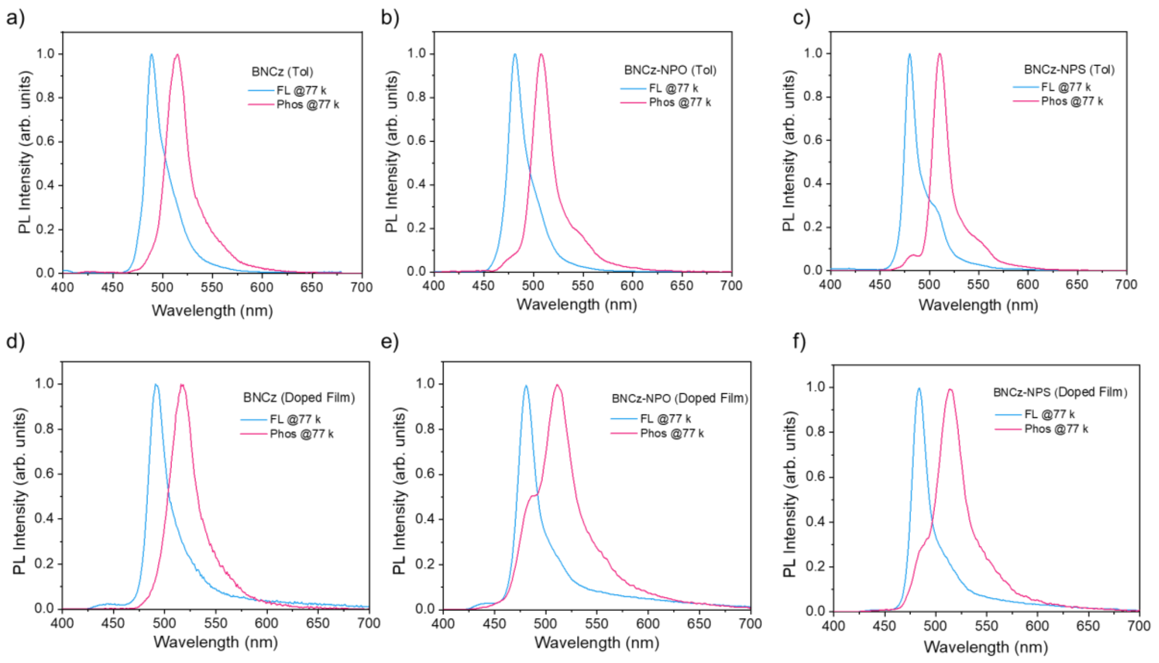

Supplementary Fig. 13 | Low-temperature fluorescence and phosphorescence spectra. Fluorescence (blue line) and phosphorescence (red line) spectra of a) BNCz, b) BNCz-NPO and c) BNCz-NPS in toluene solution (1×10^{-5} M, 77 K). Fluorescence (blue line) and phosphorescence (red line) spectra of d) BNCz, e) BNCz-NPO and f) BNCz-NPS in doped films (3 wt% in PhCzBCz).

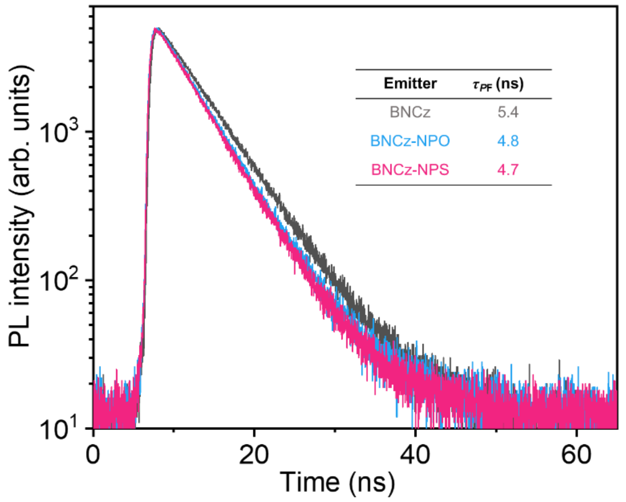

Supplementary Fig. 14 | Transient PL decay. Transient PL decay curves of BNCz, BNCz-NPO and BNCz-NPS in oxygen-free toluene solution using a picosecond pulsed diode laser (λ_{ex} = 375 nm).

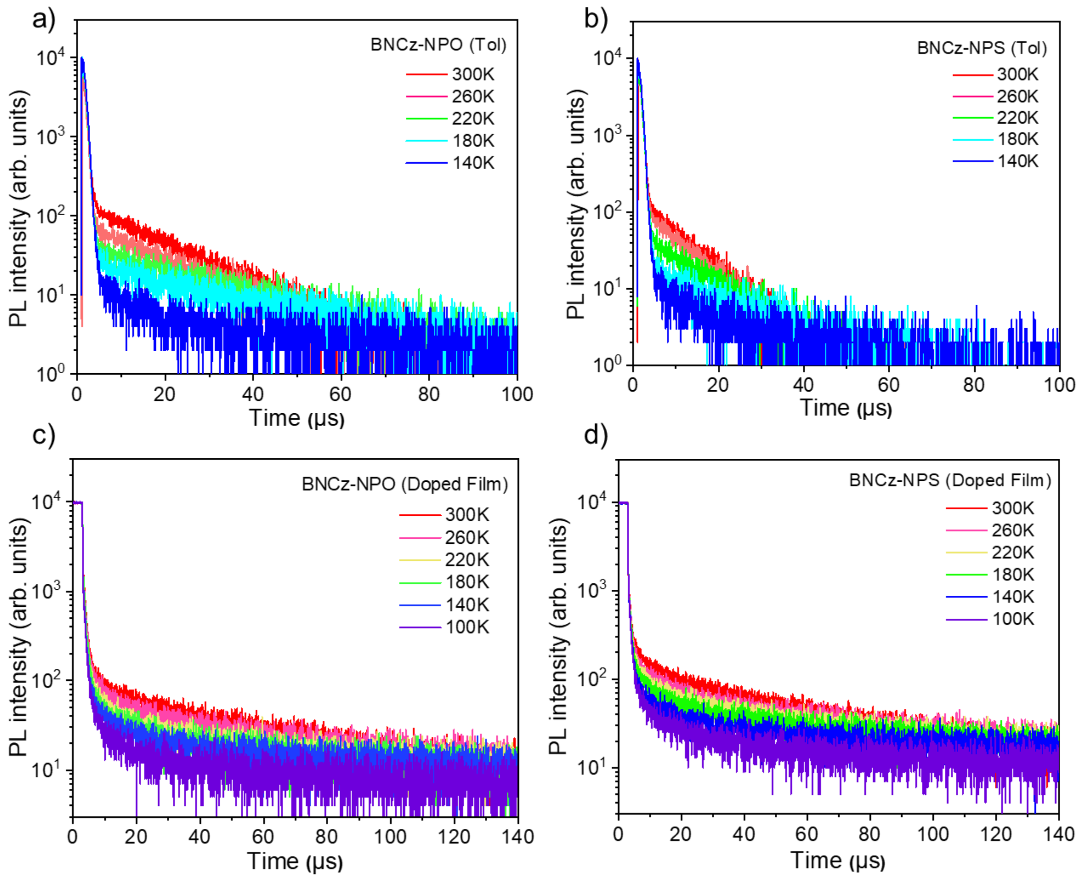

Supplementary Fig. 15 | Temperature-dependent transient PL decay. Temperature-dependent transient PL decays of a) BNCz-NPO and b) BNCz-NPS in oxygen-free toluene solution and Temperature-dependent transient PL decays of c) BNCz-NPO and d) BNCz-NPS in doped films (3 wt% in PhCzBCz).

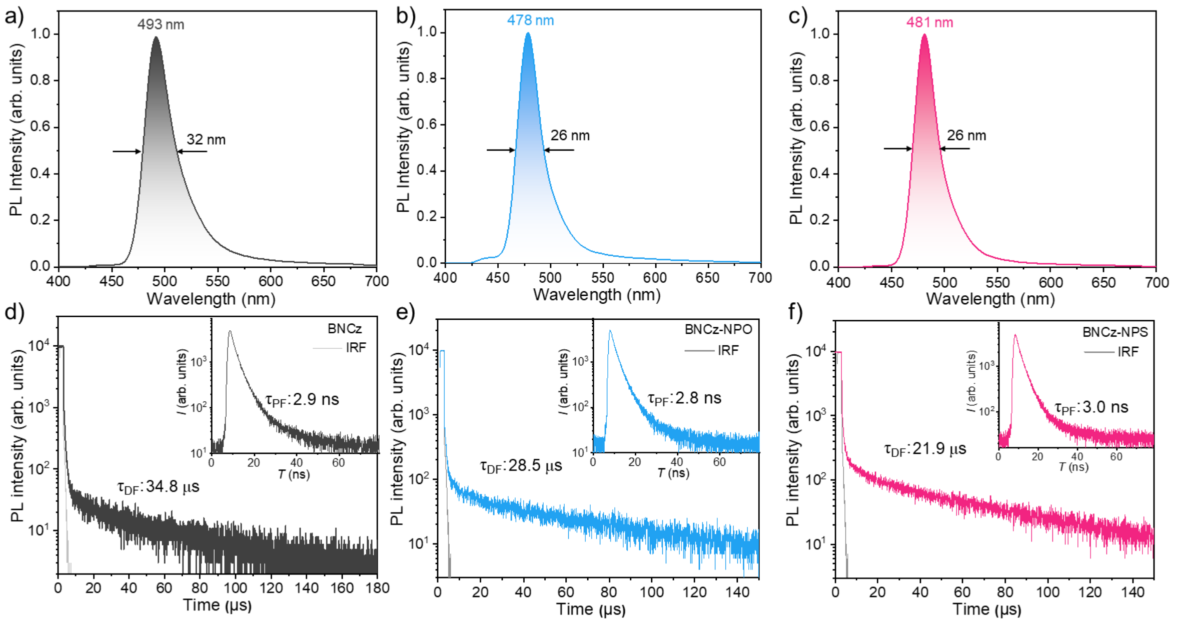

Supplementary Fig. 16 | PL spectra and transient PL decay. PL spectra of a) BNCz, b) BNCz-NPO and c) BNCz-NPS doped films (3 wt% in PhCzBCz) at 300 K. Transient PL decay curves of d) BNCz, e) BNCz-NPO and f) BNCz-NPS doped films (3 wt% in PhCzBCz) at 300 K.

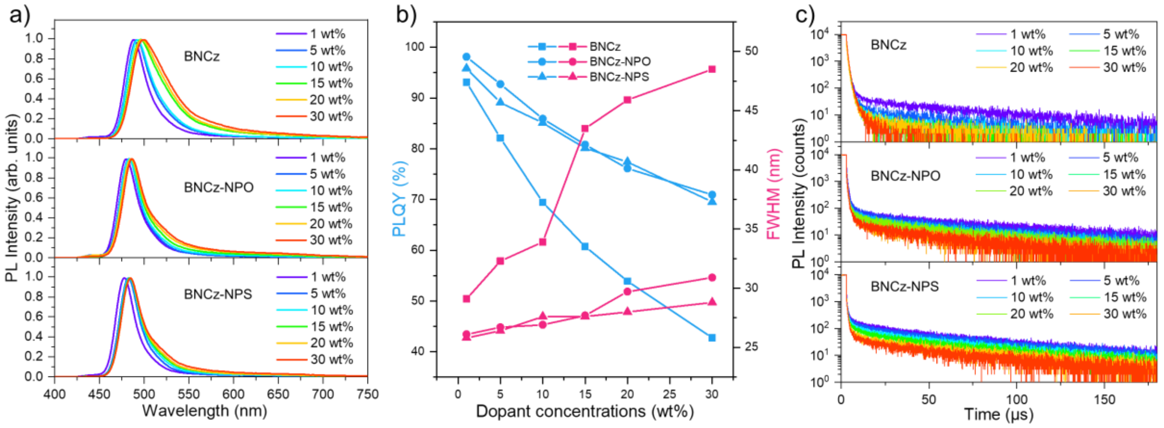

Supplementary Fig. 17 | Concentration-dependent luminescence characteristics. a) PL spectra. b) Plots for the FWHM and PLQY versus dopant concentration. c) Transient PL decay curves for BNCz, BNCz-NPO and BNCz-NPS as a dopant in PhCzBCz-hosted film with different doping levels

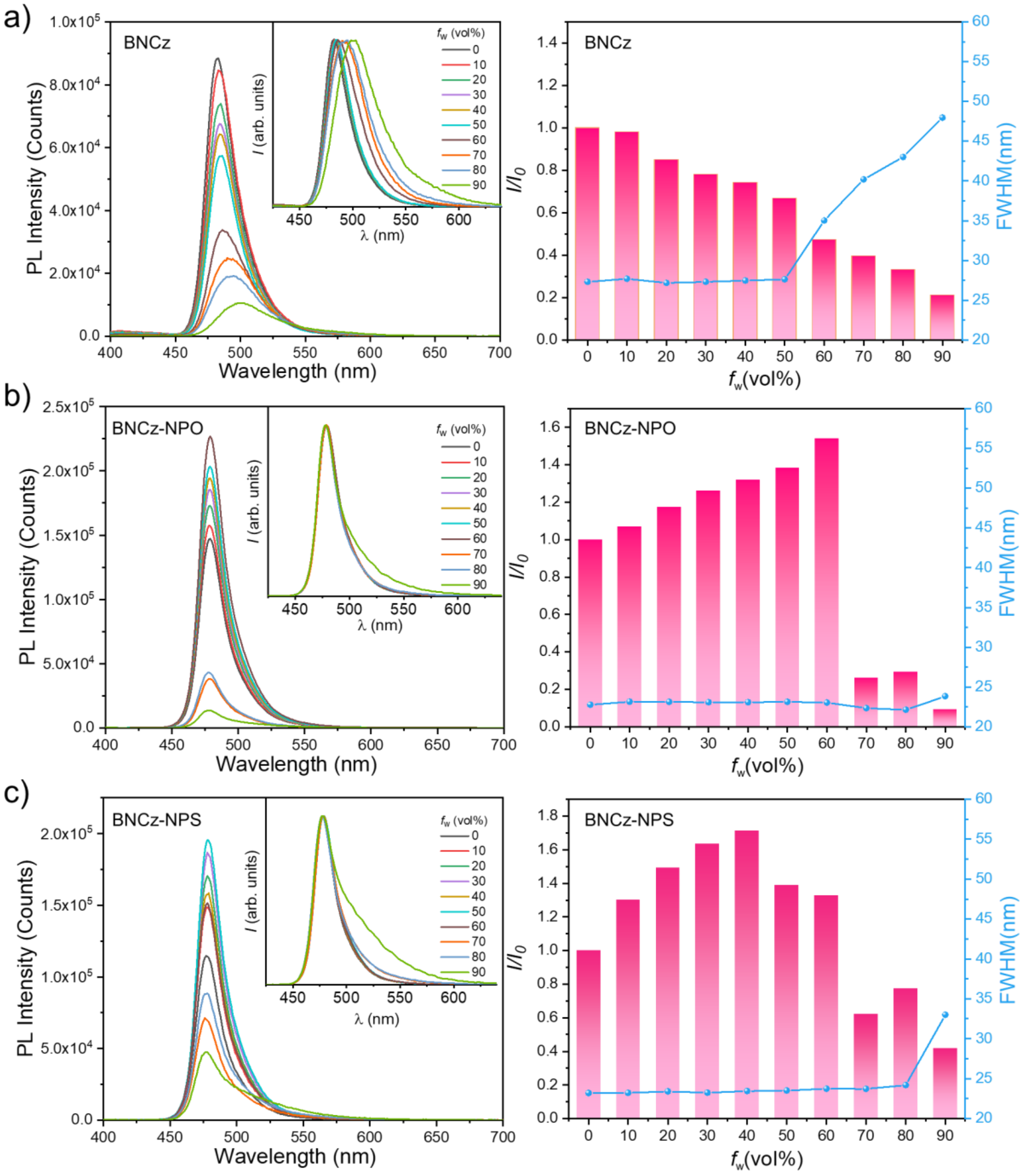

Supplementary Fig. 18 | PL spectra in THF/water mixtures with different water fractions (f_w). a) BNCz, b) BNCz-NPO and c) BNCz-NPS in THF/water mixtures with different water fractions (f_w). Left panel: PL spectra in THF/water mixtures with different f_w; right panel: plots of the relative PL intensity (I/I_0) and full-width at half-maximum (FWHM) of PL spectra versus f_w in a THF/water mixtures. Concentration: 10 μM.

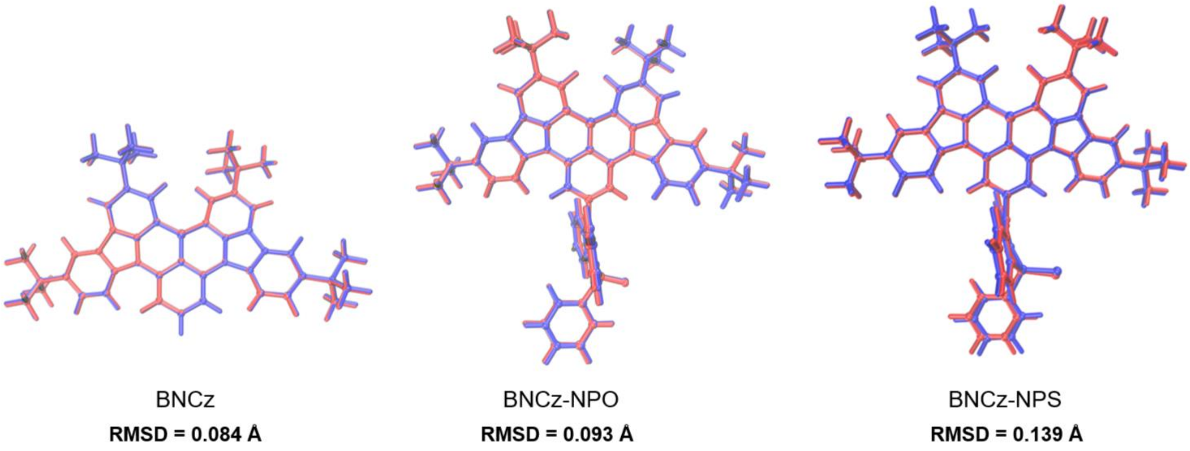

Supplementary Fig. 19 | RMSD value analysis. The RMSD values of BNCz, BNCz-NPO, and BNCz-NPS between the optimized S_0 (red) and S_1 (blue) geometries.

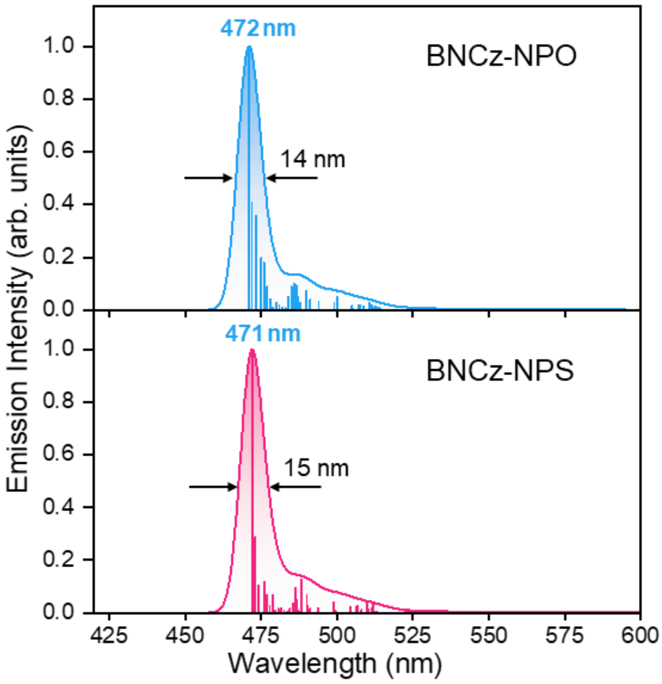

Supplementary Fig. 20 | Simulated emission spectra. Emission spectra simulated by Franck–Condon analysis of S_1–S_0 transition, the vertical lines are the vibrational transition dipoles with strength > 0.001.

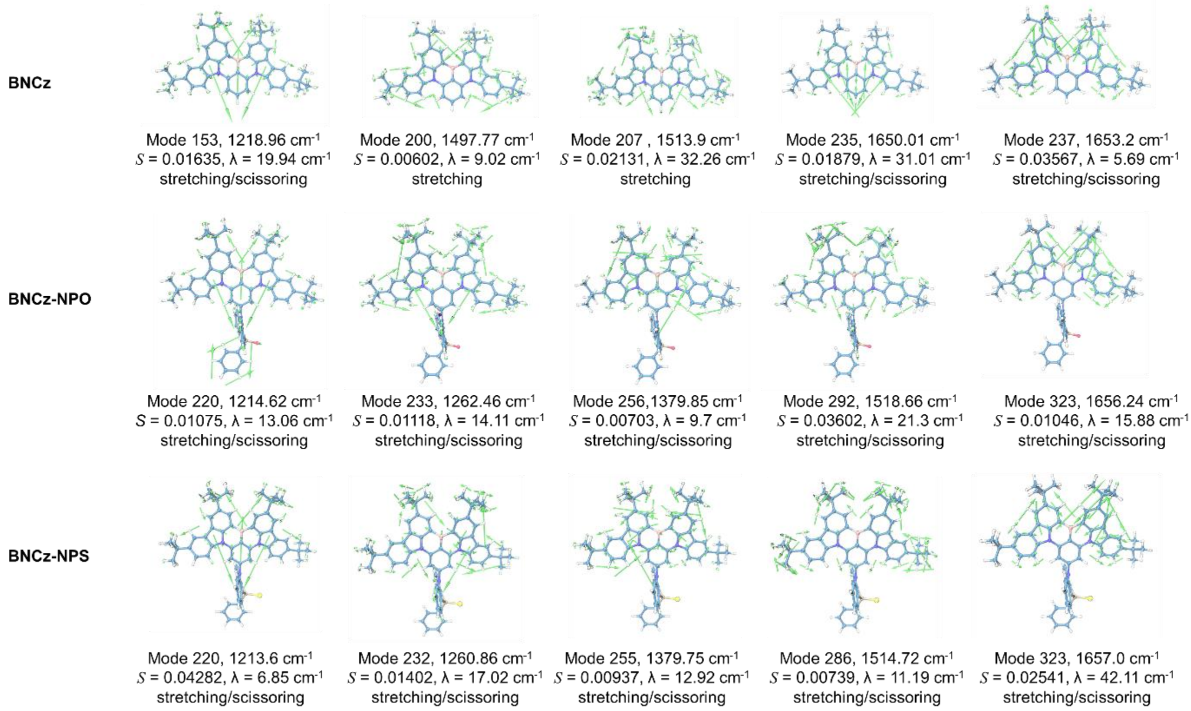

Supplementary Fig. 21 | The dominant vibration mode analysis. Transition frequencies, *S*, and reorganization energies of the vibrational modes contribute to the S_1-S_0 transition of the parent molecule BNCz, BNCz-NPO, and BNCz-NPS in the region of frequency > 1000 cm^{-1}.

Supplementary Table 5 | Summary of Franck-Condon analysis on the S_1–S_0 transition of BNCz, BNCz-NPO, and BNCz-NPS at the (TD) B3LYP/6-31G(d) level.

Compd.	Transition (S_1→S_0)	Frequency (cm^{-1})	Relative frequency (cm^{-1})	Line density	Dipole strength (arb. units)
BNCz	0→0	21649.5367	0	666.1	0.5347
	$0→2^1$	21632.2176	-17.3191	193.6	0.1562
	$0→3^1$	21617.3416	-32.1951	114.5	0.09271
	$0→7^1$	21609.3069	-40.2298	84.81	0.06875
	$0→9^1$	21591.5786	-57.9581	544.0	0.4420
	$0→9^1,2^1$	21574.2594	-75.2773	152.5	0.1242
	$0→9^1,3^1$	21559,3835	-90.1532	93.15	0.07614
	$0→9^1,7^1$	21551.3487	-98.1880	75.69	0.06196
	$0→9^2$	21533.6204	-115.9163	224.4	0.1841
BNCz-NPO	0→0	21901.2363	0	717.2	0.5498
	$0→3^1$	21889.7477	-11.4886	395.9	0.3044
	$0→3^2$	21878.2591	-22.9772	114.2	0.08792
	$0→11^1$	21862.5103	-38.7260	112.7	0.08697
	$0→14^1$	21844.5658	-56.6705	126.2	0.09795
	$0→15^1$	21842.9812	-58.2551	101.9	0.07905

BNCz-NPS	$0\rightarrow0$	21860.4119	0	426.5	0.3293
	$0\rightarrow1^1$	21857.0276	-3.3843	408.5	0.3159
	$0\rightarrow1^2$	21853.6433	-6.7686	101.3	0.07834
	$0\rightarrow3^1$	21849.7412	-10.6707	97.82	0.07577
	$0\rightarrow3^1,1^1$	21846.3569	-14.0550	76.24	0.05903
	$0\rightarrow14^1$	21804.5658	-55.8461	93.18	0.07270
	$0\rightarrow15^1$	21802.4181	-57.9938	76.94	0.06013
	$0\rightarrow14^1,1^1$	21801.1815	-59.2304	93.55	0.07310
	$0\rightarrow15^1,1^1$	21799.0338	-61.3781	74.94	0.05854

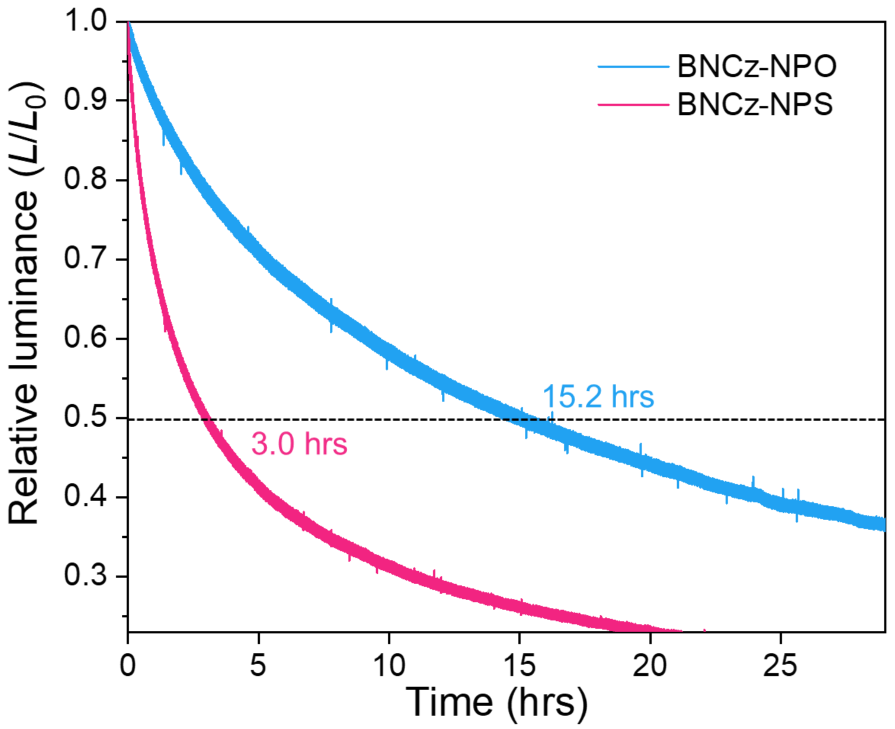

Supplementary Fig. 22 | EL stabilities of OLEDs. The lifetime of the OLEDs based on BNCz-NPO and BNCz-NPS as an emitter with initial luminance at 100 cd m^{-2}.

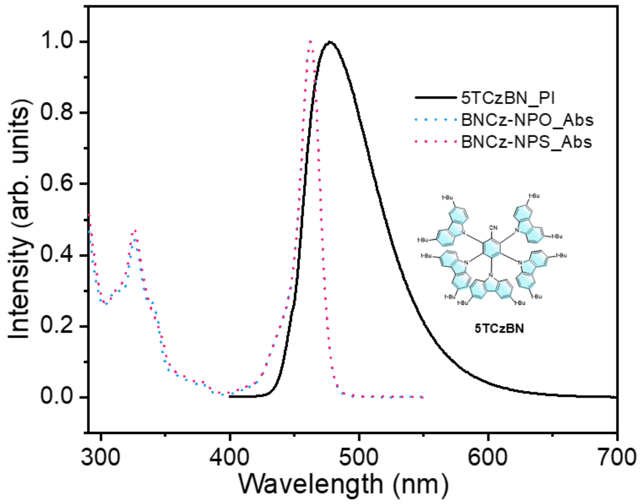

Supplementary Fig. 23 | The overlap between absorption and PL spectra. Absorption spectra of BNCz-NPO and BNCz-NPS, and PL spectrum of 5TCzBN in toluene solution (1 × 10^{-5} M, 298 K).

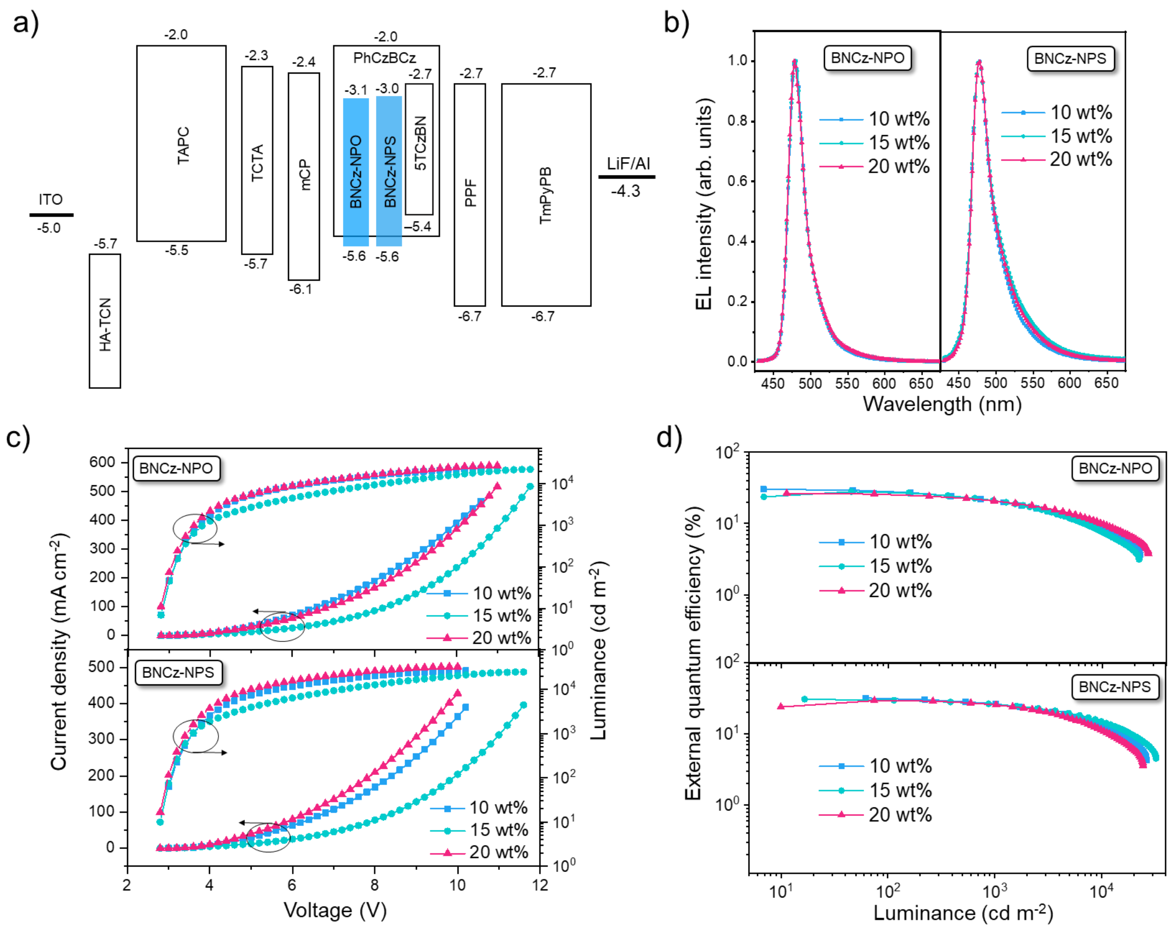

Supplementary Fig. 24 | Device structure and EL performances of HF-I type OLEDs. a) Device structure of HF-I type OLEDs based on BNCz-NPO and BNCz-NPS, with different doping ratio (10 wt%, 15 wt%, and 20 wt%) of 5TCzBN. b) EL spectra measured at 100 cd m^{-2}. c) Current density-voltage-luminance characteristics. d) EQE-luminance curves.

Supplementary Table 6 | Summary of the performances for HF- I type OLEDs based on BNCz-NPO and BNCz-NPS.

Emitter	Conc. [wt%]	V_{on} [a)] [V]	L_{max} [b)] [cd m^{-2}]	CE [c)] [cd A^{-1}]	PE [d)] [lm W^{-1}]	EQE [e)] [%]	λ_{EL} [f)] [nm]	FWHM [g)] [nm]	CIE [h)] [x, y]
BNCz-NPO	10 wt%	2.8	22660	40.6/37.5/27.6	45.5/37.7/23.3	30.2/28.0/20.7	480	26	(0.12, 0.22)
BNCz-NPO	15 wt%	2.8	22230	38.2/37.4/28.6	40.0/37.6/23.4	27.9/27.3/20.8	480	26	(0.12, 0.22)
BNCz-NPO	20 wt%	2.8	27220	36.6/35.5/28.7	41.1/36.8/25.0	26.4/25.5/20.6	478	26	(0.12, 0.22)
BNCz-NPS	10 wt%	3.0	26330	50.1/49.4/41.5	52.4/50.3/36.1	31.9/31.6/26.4	480	30	(0.13, 0.24)
BNCz-NPS	15 wt%	2.8	24580	50.4/49.5/44.1	52.7/51.8/38.2	29.4/29.2/25.6	478	32	(0.14, 0.26)
BNCz-NPS	20 wt%	2.8	32410	51.4/50.1/43.8	57.7/53.0/40.0	30.7/29.7/25.9	478	32	(0.13, 0.24)

[a] Voltage measured at 1 cd m^{-2}; [b] maximum luminance; [c] maximum current efficiency at maximum, 100, 1000 cd m^{-2}, respectively; [d] maximum power efficiency at maximum, 100, 1000 cd m^{-2}, respectively; [e] external quantum efficiency at maximum, 100, 1000 cd m^{-2}, respectively; [f] EL peak; [g] full width at half maximum of EL spectrum; [h] recorded at 4 V.

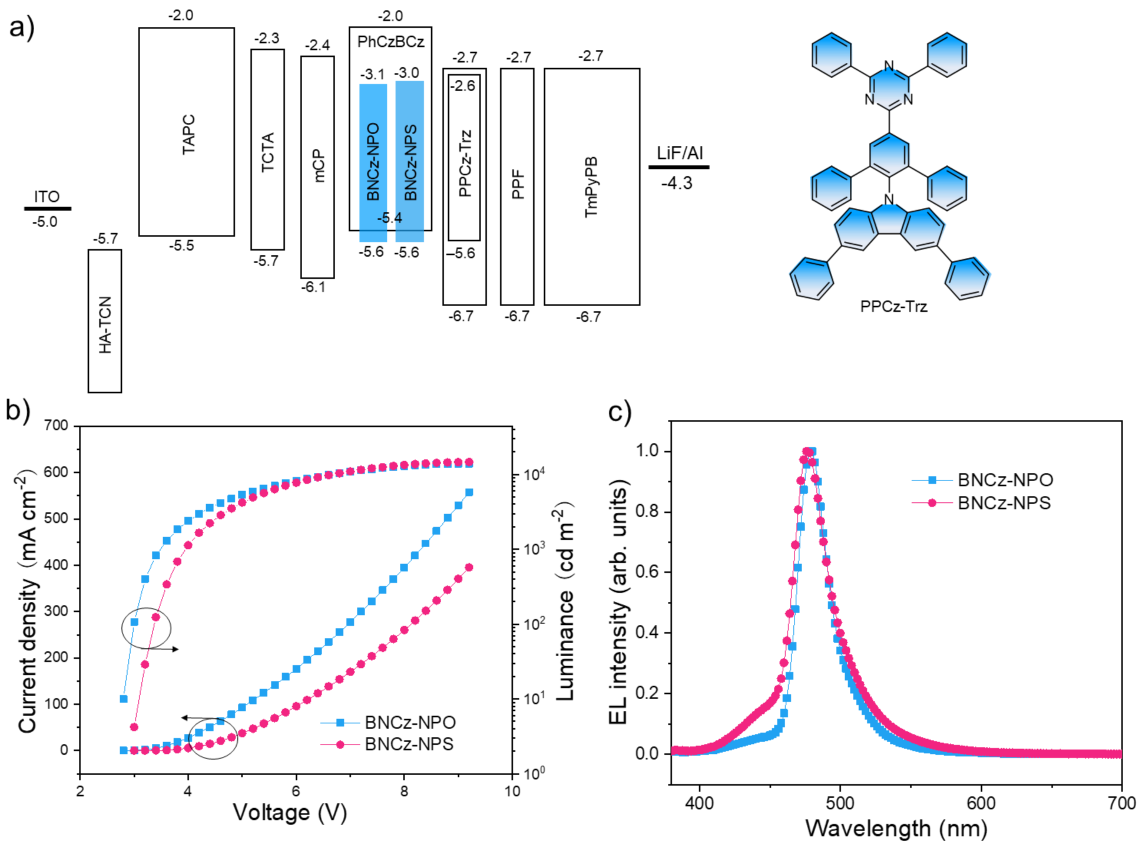

Supplementary Fig. 25 | Device structure and EL performances of HF-II type OLEDs. a) Device structure of HF-II type OLEDs and the chemical structure of PPCz-Trz. b) Current density-voltage-luminance characteristics of HF-II type OLEDs. c) EL spectra measured at 4 V.

Supplementary Table 7 | EL performance of the reported high-performance blue MR-TADF emitters. The plotted data for Fig. 6c in main text are highlighted with color.

Dopant type	Emitter	λ_{EL} [nm]	FWHM [nm]	EQE_{max} [%]	EQE_{100} [%]	EQE_{1000} [%]	CIE (x, y)	Ref.
Mono-boron	**BNCz-NPO**	**480**	**26**	**32.1**	**21.7**	**9.7**	**(0.11, 0.22)**	**This work**
	**BNCz -NPS**	**476**	**26**	**29.6**	**23.5**	**13.5**	**(0.11, 0.16)**	**This work**
	**BNCz-NPO (HF-I)**	**476**	**26**	**30.2**	**28.0**	**20.7**	**(0.12, 0.22)**	**This work**
	**BNCz-NPO (HF-II)**	**480**	**26**	**37.6**	**30.2**	**12.0**	**(0.11, 0.18)**	**This work**
	**BNCz-NPS (HF-I)**	**480**	**30**	**31.9**	**31.6**	**26.4**	**(0.14, 0.24)**	**This work**
	**BNCz-NPs (HF-II)**	**476**	**30**	**32.2**	**29.2**	**17.3**	**(0.13, 0.18)**	**This work**
	TBA-BCz-BN (HF)	470	28	30.8	19.0	11.7	(0.12, 0.15)	7
	DCzBNO (HF)	469	45	31.7	-	-	(0.13, 0.15)	8
	TCzBNO(HF)	482	52	36.5	-	-	(0.13, 0.25)	
	γ-Cb-B	461	28	19.0	16.2	7.7	(0.13, 0.13)	9
	Cz-B	482	30	22.6	16.6	6.9	(0.11, 0.31)	
	Tip-DtCzB (HF)	484	25	29.0	23.2	11.0	(0.10, 0.29)	10
	tDPA-DtCzB (HF)	480	27	30.2	27.5	18.0	(0.11, 0.24)	
	BNO-DPAC (HF)	448	36	27.7	-	14.5	(0.151, 0.083)	11
	BNO-DMAC (HF)	449	36	28.7	-	18.2	(0.149, 0.090)	
	BNO-tBuDMAC	447	59	27.7	-	18.8	(0.155, 0.144)	
	t-DAB-DPA	459	26	27.9	21.8	8.1	(0.13, 0.08)	12
	t-DABNA	464	26	28.4	14.8	4.4	(0.13, 0.10)	
	PAB	456	31	14.7	-	-	(0.145, 0.076)	13
	2tPAB	456	27	16.8	-	-	(0.145, 0.076)	
	3tPAB	460	26	19.3	-	-	(0.141, 0.076)	
	tDPAC-BN (HF)	460	28	21.6	15.3	5.4	(0.135, 0.094)	14
	tDMAC-BN (HF)	472	34	22.3	19.0	10.4	(0.116, 0.186)	
	DPACzBN1	475	34	23.6	19.3	9.6	(0.14, 0.30)	15
	DPACzBN2	469	28	24.0	23.5	14.3	(0.13, 0.16)	
	DPACzBN3	472	24	27.7	19.3	6.7	(0.12, 0.18)	
	B-dpa-Cz	475	27	20.1	15.3	4.7	(0.11, 0.19)	16
	B-dpa-dmAc	478	32	24.3	23.3	14.9	(0.11, 0.22)	
	B-dpa-SpiroAc	478	32	24.3	23.6	15.6	(0.11, 0.22)	
	B-O-dpa	443	32	16.3	6.5	2.2	(0.15, 0.05)	17
	B-O-Cz	481	63	13.4	9.5	5.9	(0.13, 0.22)	
	B-O-dmAc	475	44	16.2	13.5	8.4	(0.12, 0.21)	
	B-O-dpAc	473	42	17.0	14.3	9.6	(0.12, 0.20)	
	mono-mx-CzDABNA (HF)	481	48	21.4	14.0	7.8	(0.13, 0.24)	18
	tri-mx-CzDABNA (HF)	472	34	26.9	26.1	24.8	(0.13, 0.19)	
	DABNA-2	467	28	20.2	-	-	(0.12, 0.13)	19
	DABNA-1	459	28	13.5	-	-	(0.13, 0.09)	
	DABNA-NP-TB	457	33	19.5	17.5	12	(0.14, 0.11)	20
	Cz2DABNA-NP-M/TB	477	27	21.8	20.4	14.9	(0.11, 0.23)	
	mBP -DABNA-Me	468	28	24.3	19.5	16.0	(0.12, 0.14)	21
	tCBN-DADPO	472	28	30.8	23.3	16.2	(0.14, 0.22)	22
	CzBO	448	30	16.3	10.7	4.7	(0.15, 0.05)	23
	CzBS	474	31	29.1	27.4	20.0	(0.11, 0.16)	
	CzBSe	481	33	30.1	29.7	26.1	(0.10, 0.24)	
	DCz-BSN	473	29	22.0	18.6	10.0	(0.11, 0.17)	24
	Cz-BSN	482	32	18.9	13.4	6.8	(0.11, 0.28)	

	PTZBN3 (HF)	468	46	32.0	30.3	25.0	(0.15, 0.24)	
	PTZBN2 (HF)	478	48	34.8	28.8	19.5	(0.15, 0.29	25
	PTZBN1 (HF)	489	47	32.7	25.9	16.5	(0.14, 0.41	
	TBN-TPA	474	27	32.1	27.4	13.9	(0.12, 0.19)	26
	TCz-BN	474	34	18.88	15.19	10.52	(0.13, 0.20)	27
	ADBNA -Me-Mes	481	32	16.2	11.1	-	(0.10, 0.27)	28
	ADBNA -Me-Tip	480	33	21.4	15.4	-	(0.11, 0.29)	
	BN-TC	474	37	21.2	8.7	4.2	(0.13, 0.20)	29
	BN-AC	484	27	23.4	15.9	5.5	(0.10, 0.31)	
	Tp-DABNA (HF)	462	29	27.5	18.6	-	(0.14, 0.13)	30
	t-DABNA (HF)	464	34	23.3	14.7	-	(0.13, 0.14)	
	p-TBNCz	488	26	27.0	11.7	5.8	(0.09, 0.35)	31
	OBN	437	44	15.7	-	-	0.15, 0.09)	32
	NBN	452	40	23.02	-	-	(0.14, 0.09)	
	BIC-pCz (HF)	466	48	39.8			(0.14, 0.16)	33
	BN1 (HF)	457	28	31.2	18.3	9.3	(0.14, 0.06)	34
	BN2 (HF)	467	23	33.2	25.5	15.5	(0.13, 0.11)	
	ODBN	446	54	24.15	-	-	(0.15, 0.10)	32
	BIC-mCz (HF)	432	42	19.4	-	-	(0.16, 0.05)	33
	mDBIC (HF)	431	42	13.5	-	-	(0.16, 0.05)	
	BN3 (HF)	458	23	37.6	34.0	26.2	(0.14, 0.08)	34
	V-DABNA -Mes	480	27	22.9	20.3	10.9	(0.09, 0.21)	35
	m[B-N]N1 (HF)	479	27	36.0	-	32.3	(0.12, 0.27)	36
	m[B-N]N2 (HF)	485	33	33.4	-	29.7	(0.14, 0.46)	
	α-3BNMes (HF)	443	49	14.6	10.2	-	(0.15, 0.10)	37
	B2	460	37	18.3	12.6	-	(0.13, 0.11)	38
	v-DABNA	469	18	34.4	32.8	26.0	(0.14, 0.11)	39
Multiple-boron	v-DABNA-OMe	465	23	29.5	28.8	26.9	(0.13, 0.10)	40
	m-v-DABNA	471	18	36.2	-	-	(0.12, 0.12)	
	4F-v-DABNA	464	18	35.8	-	-	(0.13, 0.08)	41
	4F-mv-DABNA	461	18	33.7	-	-	(0.13, 0.06)	
	BOBO-Z	445	18	16.6	12.4	5.3	(0.15, 0.04)	
	BOBS-Z	455	23	33.1	30.2	20.2	(0.14, 0.06)	42
	BSBS-Z	463	23	32.2	30.2	20.2	(0.13, 0.08)	
	m-DiNBO	466	21	24.2	17.3	-	(0.13, 0.10)	43
	BSBS-N1	478	21	25	-	-	(0.11, 0.22)	44
	NO-DBMR	469	26	33.7	-	-	(0.12,0.12)	45
	Cz-DBMR	491	22	29.8	-	-	(0.09,0.34)	
	BBCz-DB	469	27	29.3	-	-	(0.12, 0.18)	46

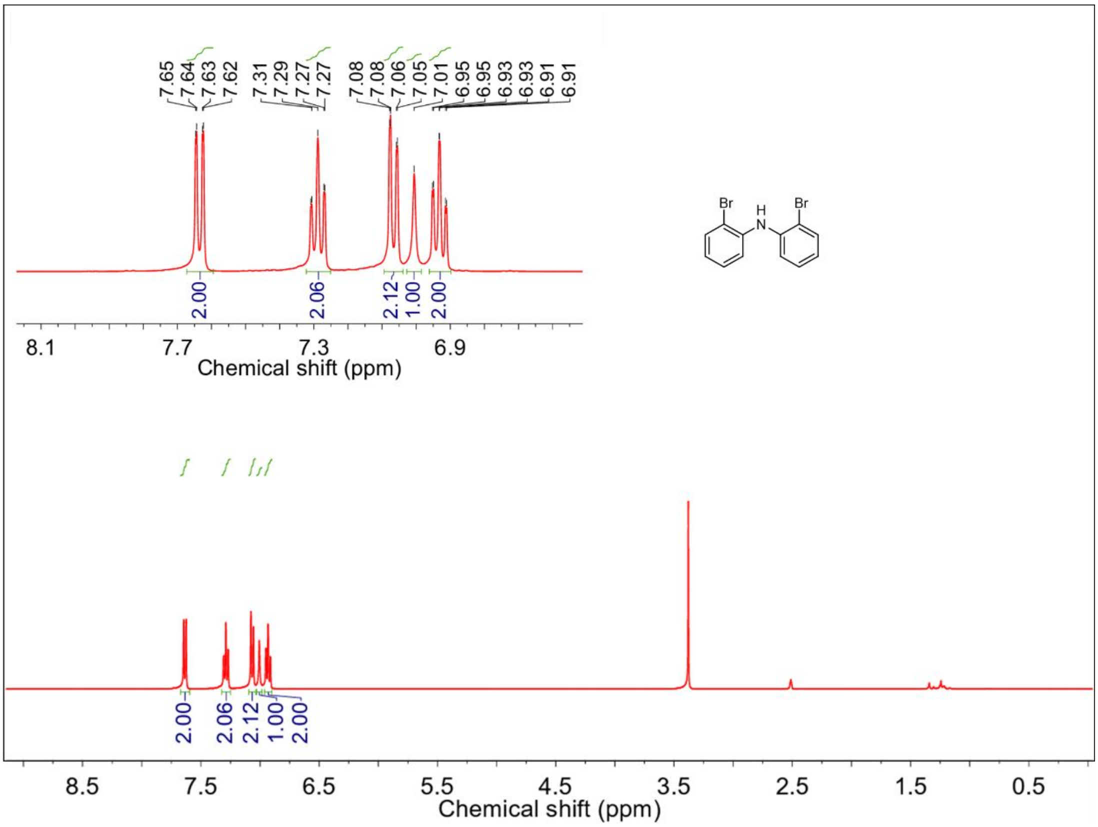

Supplementary Fig. 26 | NMR data. ^{1}H NMR spectrum of 1 in $CDCl_3$ at 25 °C.

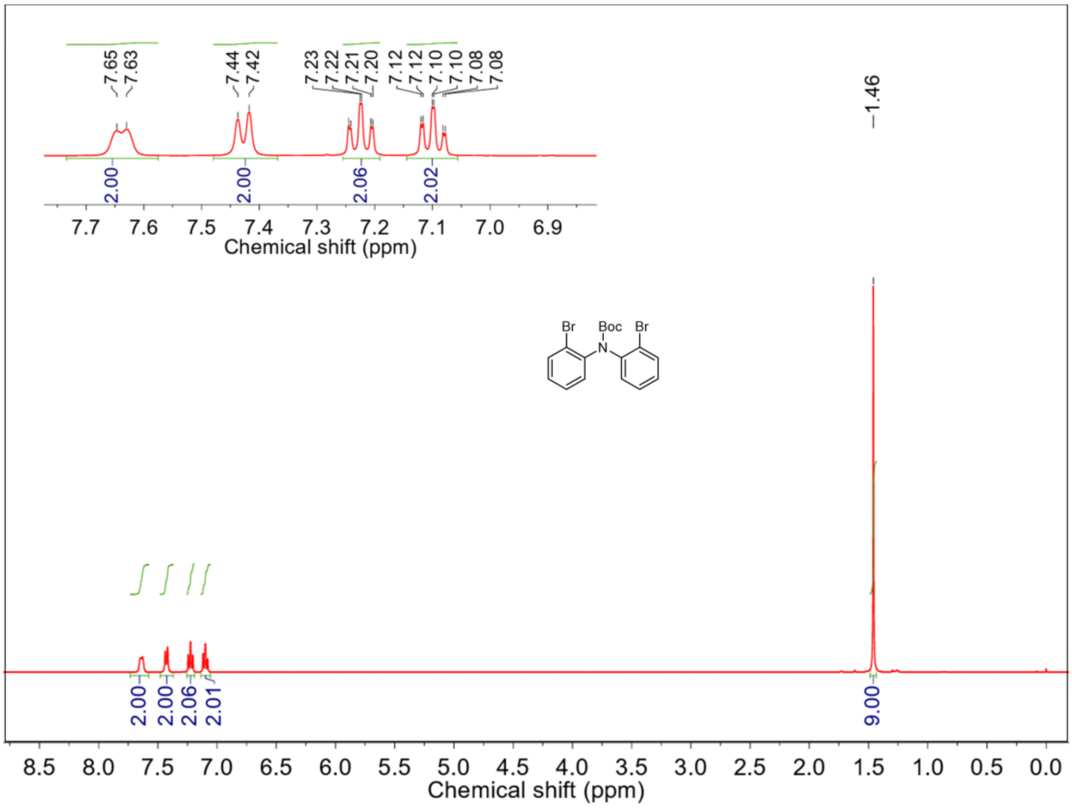

Supplementary Fig. 27 | NMR data. ^{1}H NMR spectrum of 2 in $CDCl_3$ at 25 °C.

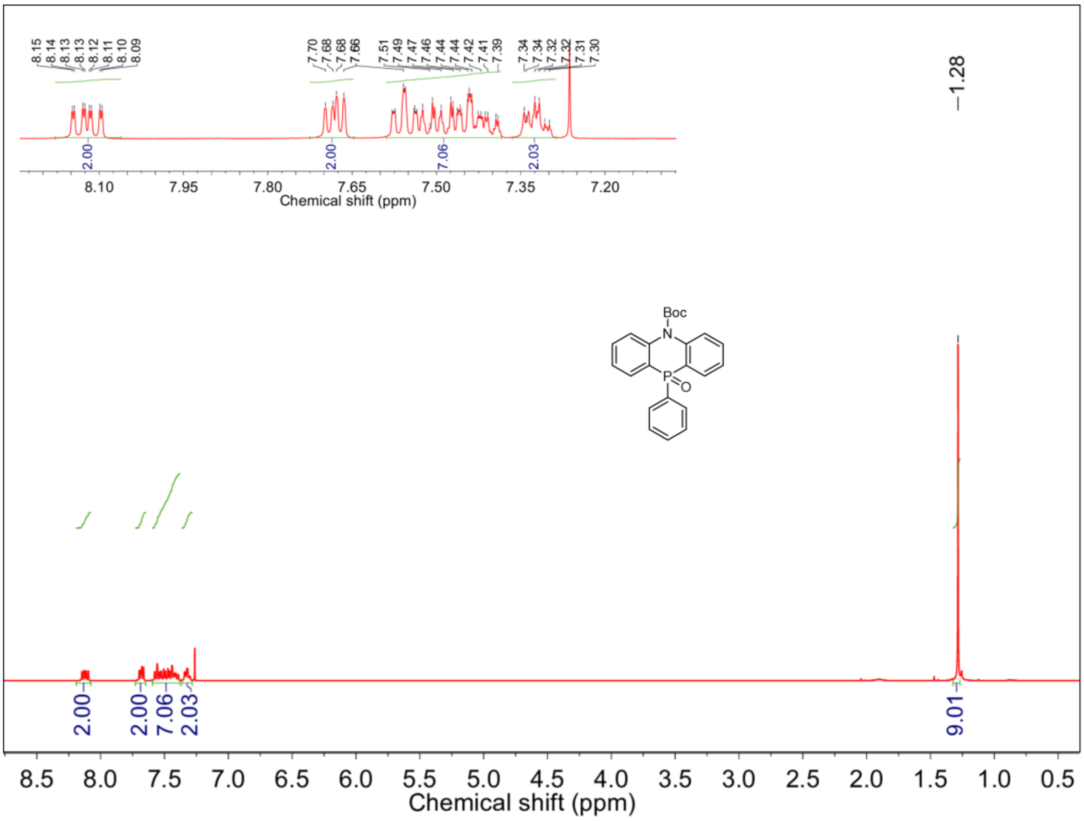

Supplementary Fig. 28 | NMR data. ^{1}H NMR spectrum of 3a in $CDCl_3$ at 25 °C.

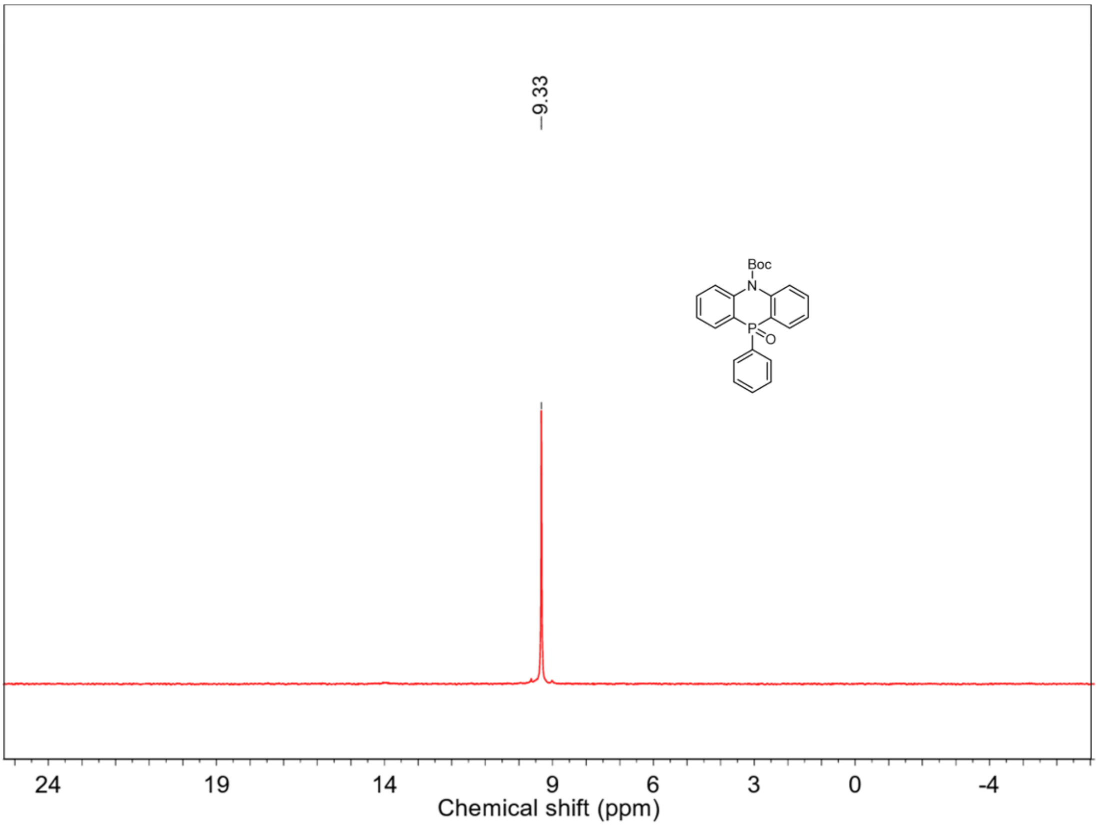

Supplementary Fig. 29 | NMR data. ^{31}P NMR spectrum of 3a in $CDCl_3$ at 25 °C.

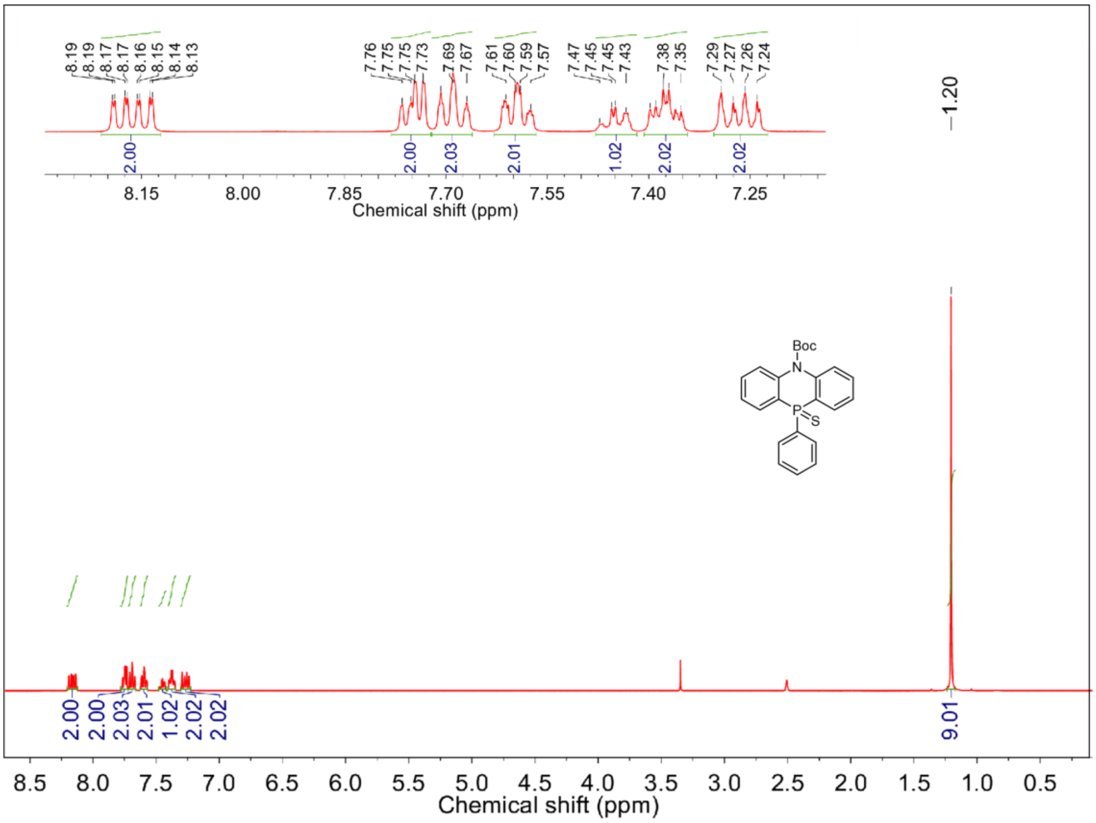

Supplementary Fig. 30 | NMR data. ^{1}H NMR spectrum of 3b in DMSO$_{d6}$ at 25 °C.

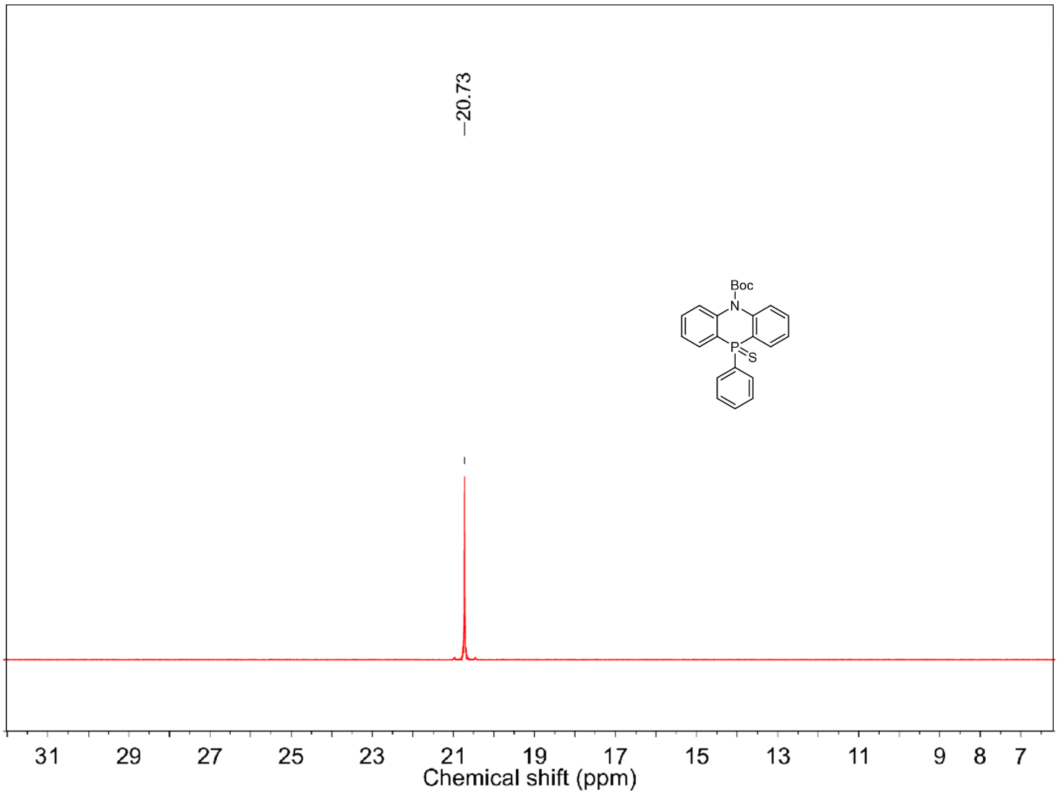

Supplementary Fig. 31 | NMR data. ^{31}P NMR spectrum of 3b in DMSO$_{d6}$ at 25 °C.

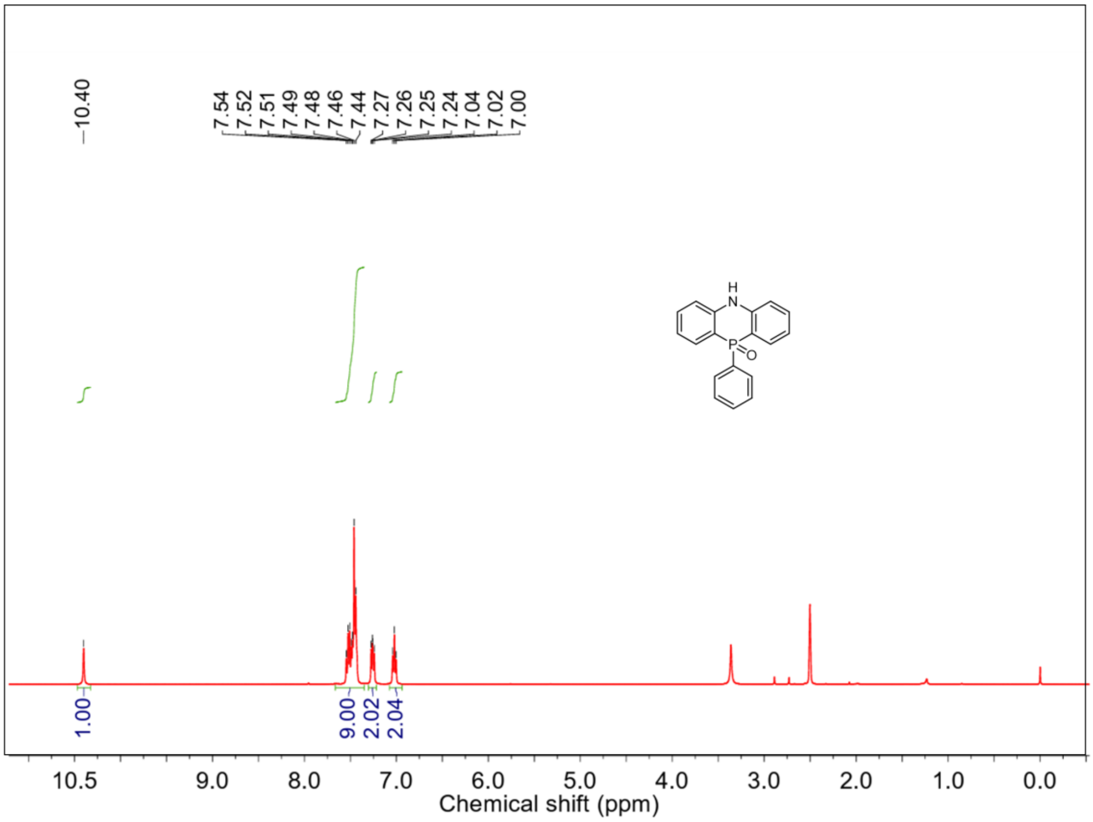

Supplementary Fig. 32 | NMR data. ^{1}H NMR spectrum of NPO in DMSO$_{d6}$ at 25 °C.

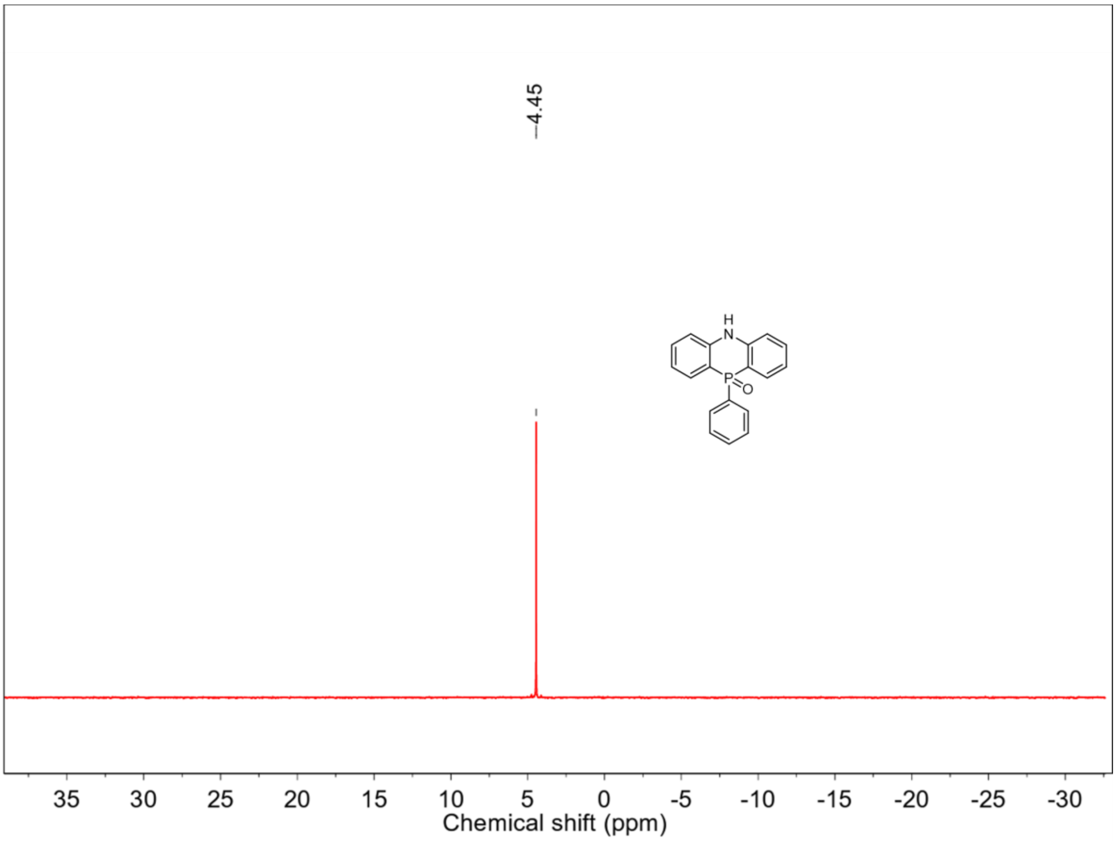

Supplementary Fig. 33 | NMR data. ^{31}P NMR spectrum of NPO in DMSO$_{d6}$ at 25 °C.

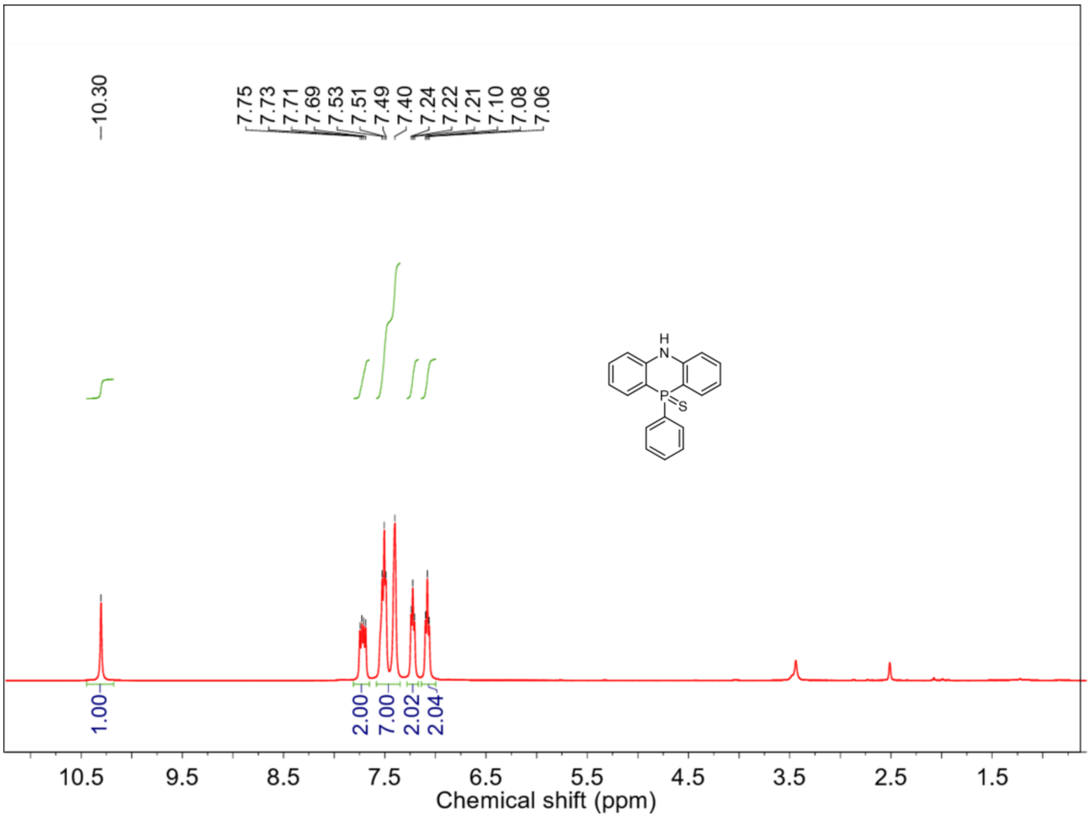

Supplementary Fig. 34 | NMR data. ^{1}H NMR spectrum of NPS in DMSO$_{d6}$ at 25 °C.

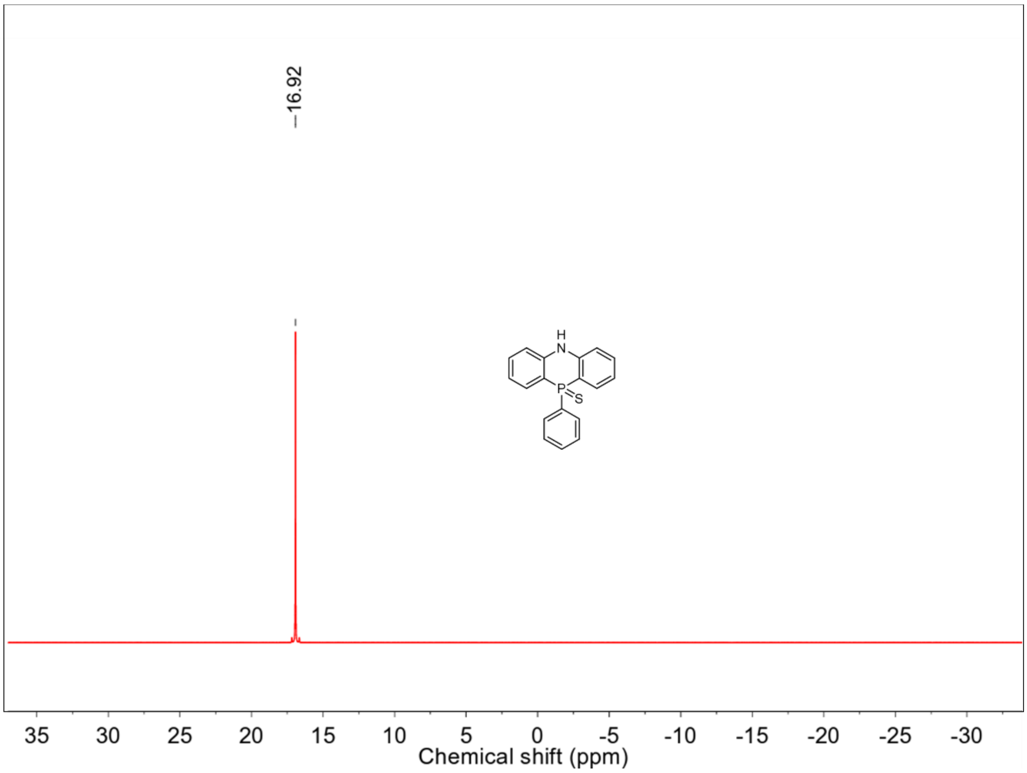

Supplementary Fig. 35 | NMR data. ^{31}P NMR spectrum of NPS in DMSO$_{d6}$ at 25 °C.

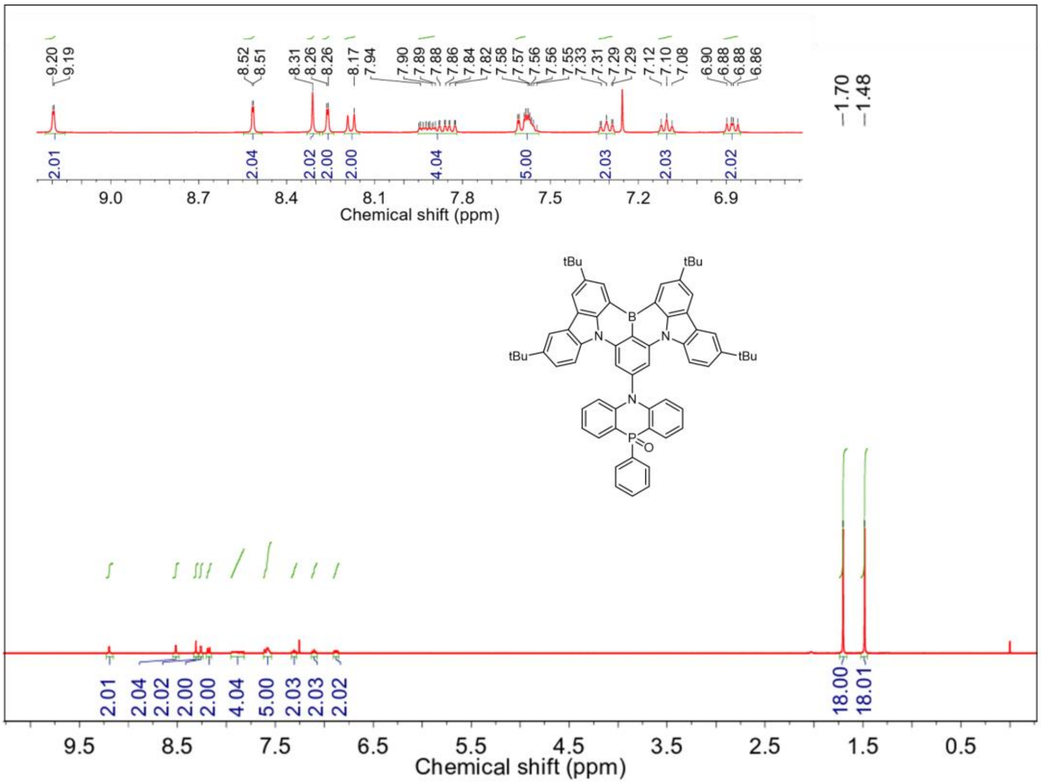

Supplementary Fig. 36 | NMR data. ^{1}H NMR spectrum of BNCz-NPO in $CDCl_3$ at 25 °C.

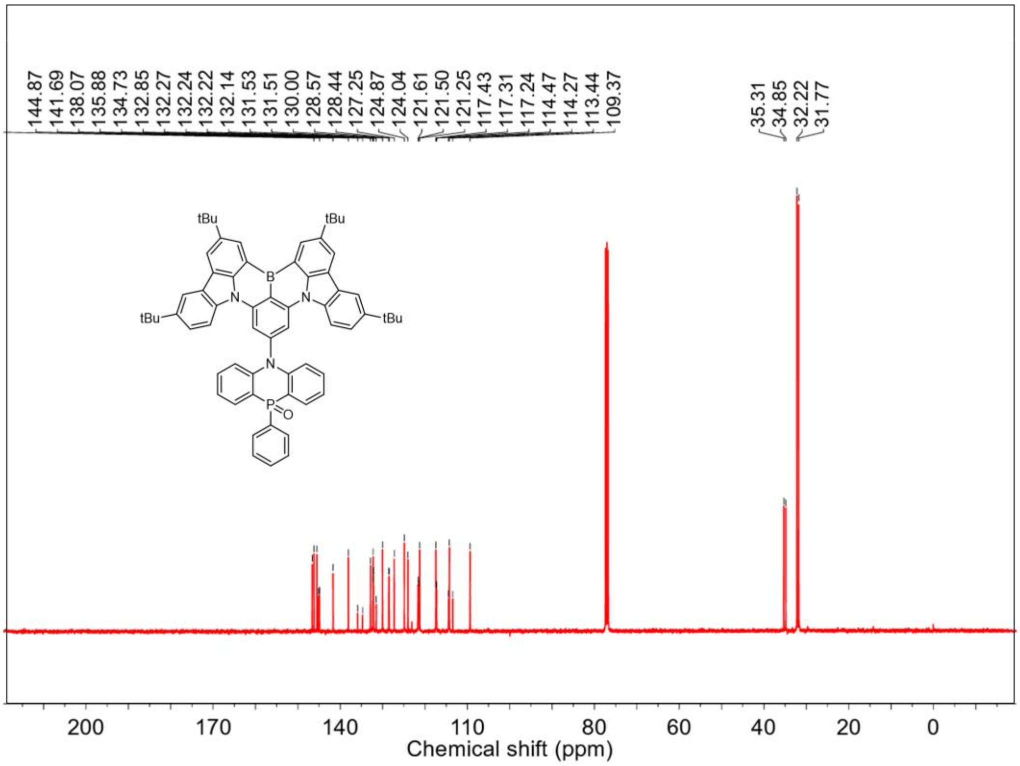

Supplementary Fig. 37 | NMR data. ^{13}C NMR spectrum of BNCz-NPO in $CDCl_3$ at 25 °C.

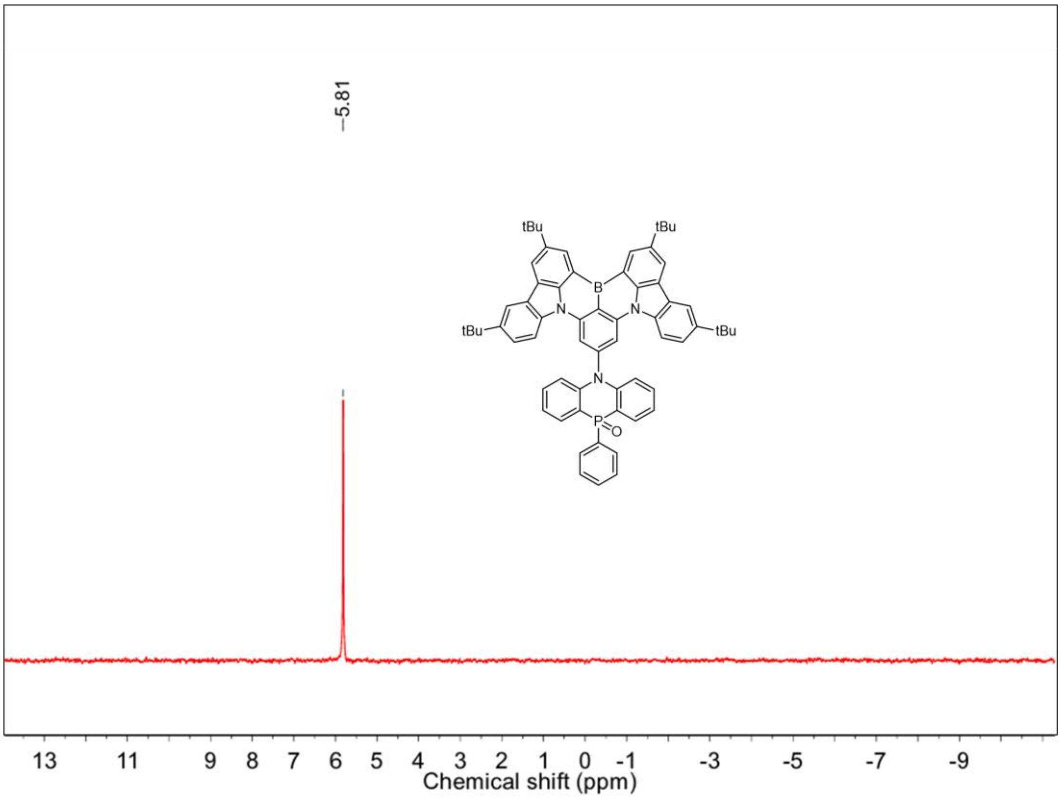

Supplementary Fig. 38 | NMR data. ^{31}P NMR spectrum of BNCz-NPO in $CDCl_3$ at 25 °C.

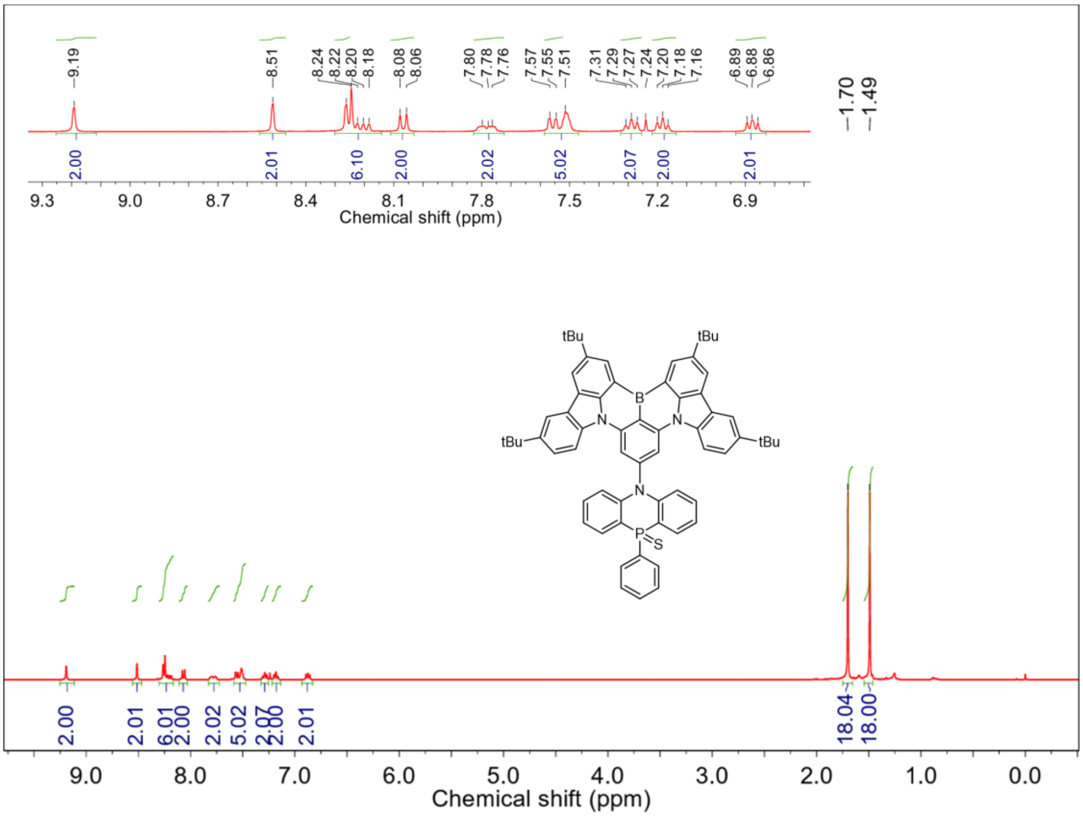

Supplementary Fig. 39 | NMR data. ^{1}H NMR spectrum of BNCz-NPS in $CDCl_3$ at 25 °C.

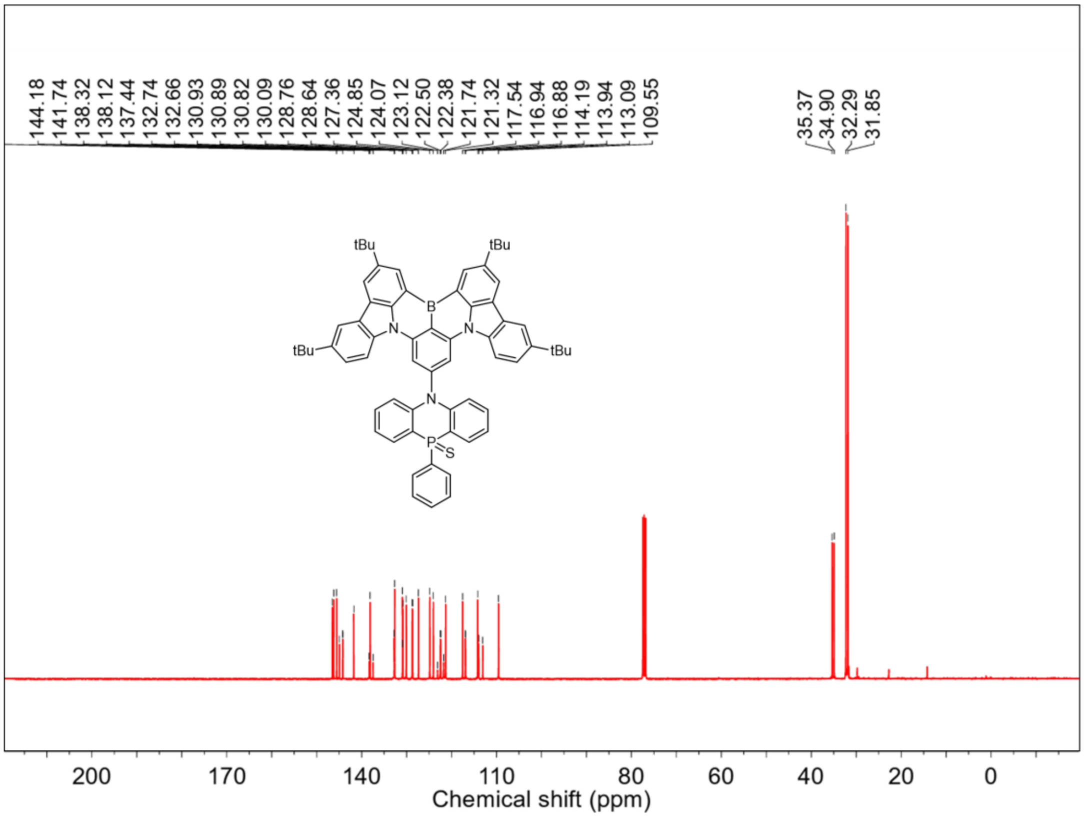

Supplementary Fig. 40 | NMR data. ^{13}C NMR spectrum of BNCz-NPS in $CDCl_3$ at 25 °C.

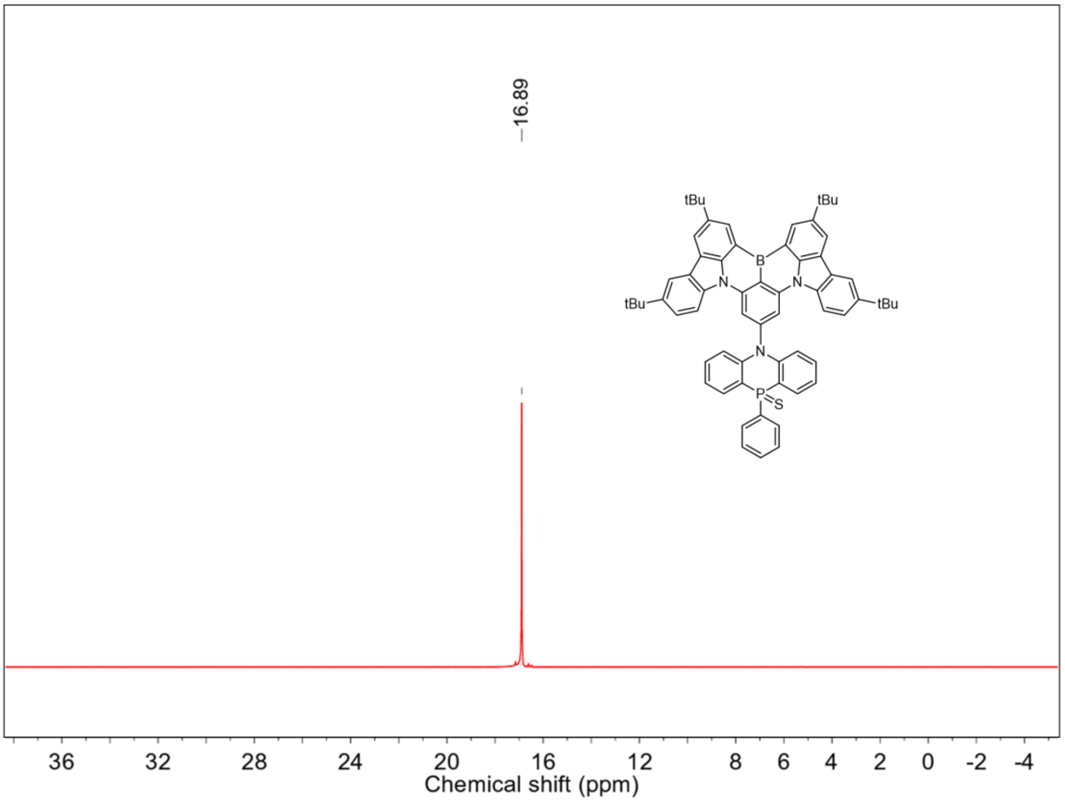

Supplementary Fig. 41 | NMR data. ^{31}P NMR spectrum of BNCz-NPS in $CDCl_3$ at 25 °C.

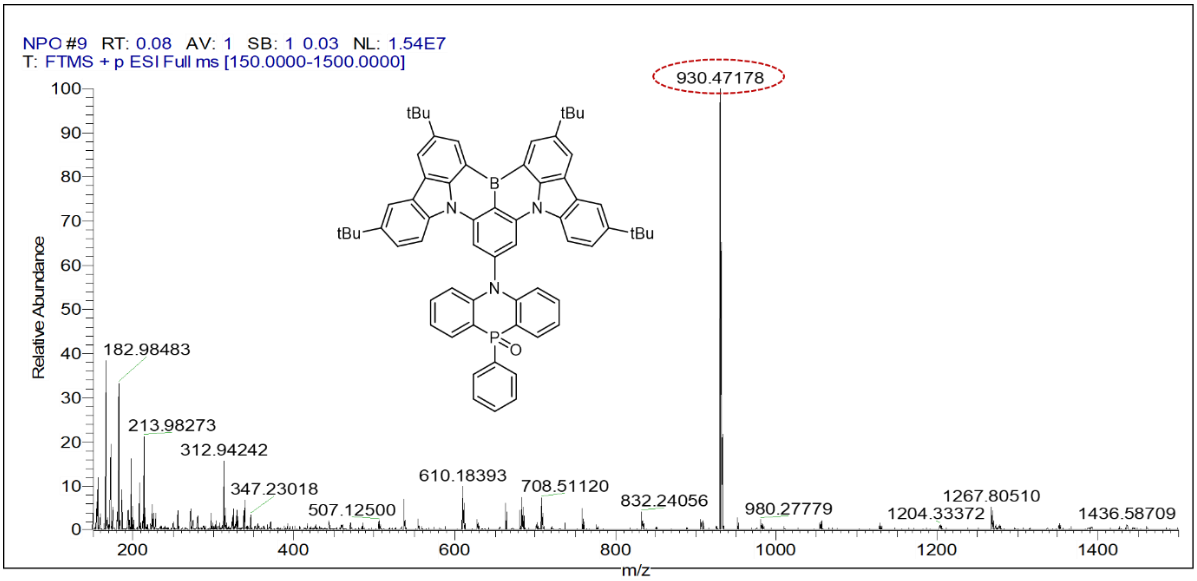

Supplementary Fig. 42 | HRMS data. HRMS spectrum of BNCz-NPO.

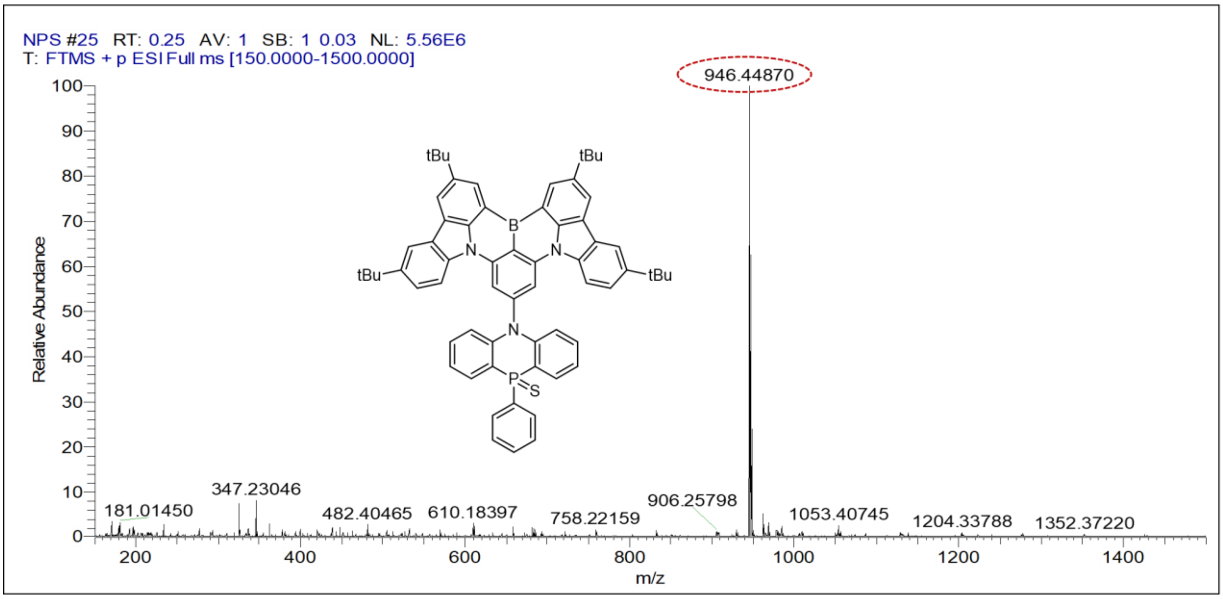

Supplementary Fig. 43 | HRMS data. HRMS spectrum of BNCz-NPS.

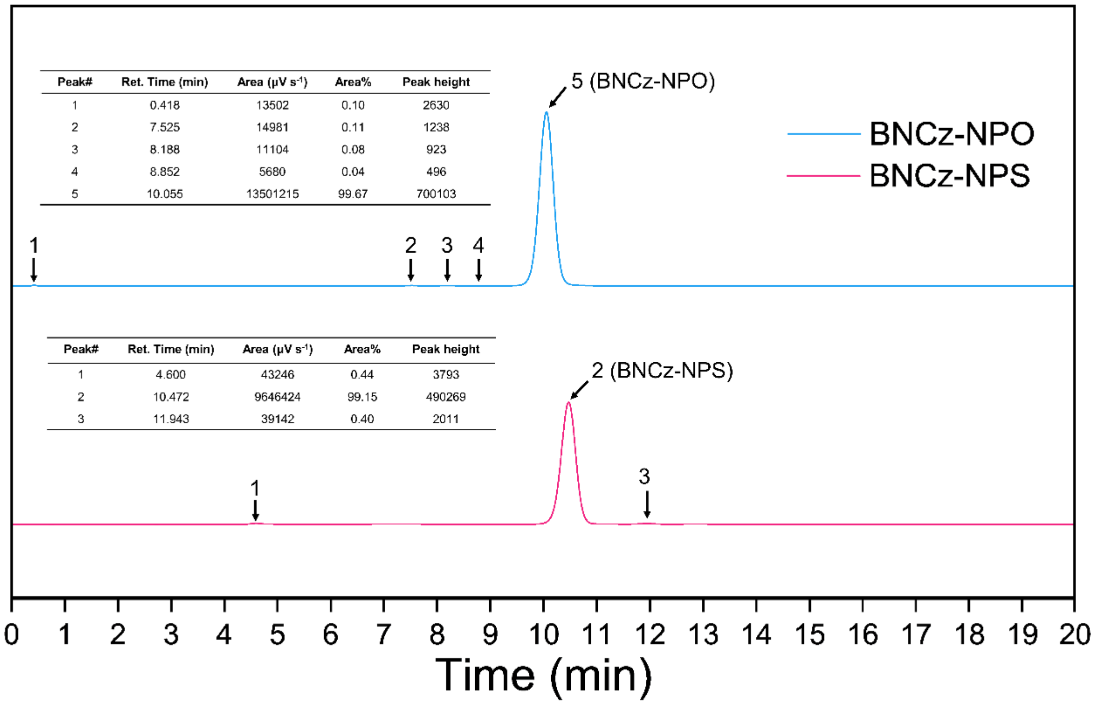

Supplementary Fig. 44 | HPLC data. HPLC spectra monitored at 254 nm with THF-water ratio of 65/35 (v/v) for the sources of BNCz-NPO and BNCz-NPS.

Supplementary References

1. Gaussian 16, Revision A.03, Frisch, M. J., et al. Gaussian, Inc., Wallingford CT, (2016).
2. Kállay, M. et al. The MRCC program system: accurate quantum chemistry from water to proteins. *J. Chem. Phys.* **152**, 074107−074124 (2020).
3. Kállay, M. et al. MRCC, a quantum chemical program suite. See www. mrcc.hu.
4. Humphrey, W., Dalke, A. & Schulten, K. VMD: Visual molecular dynamics. *J. Mol. Graphics* **14**, 33–38 (1996).
5. Lu, T. & Chen, F. Multiwfn: A multifunctional wavefunction analyzer. *J. Comput. Chem.* **33**, 580–592 (2012).
6. Masui, K., Nakanotani, H. & Adachi, C. Analysis of exciton annihilation in high-efficiency sky-blue organic light-emitting diodes with thermally activated delayed fluorescence. *Org. Electron.* **14**, 2721–2726 (2013).
7. Xiao, X., Lei, B., Wu, D. & Bin, Z. "Medium-ring" strategy enables high-performance narrowband pure-blue multi-resonance emitters: boost provided by a unique perpendicular geometry. *Chem. Commun.* **59**, 6556–6559 (2023).
8. Xu, Y. et al. B/N/O-participated multi-resonance TADF emitters by a simple peripheral decoration strategy enable high-efficiency electroluminescence with EQEs up to 36.5%. *J. Mater. Chem. C* **11**, 13733–13739 (2023).
9. Yang, M. et al. Wide-Range Color Tuning of Narrowband Emission in Multi-resonance Organoboron Delayed Fluorescence Materials through Rational Imine/Amine Functionalization. *Angew. Chem. Int. Ed.* **60**, 23142–23147 (2021).
10. Yan, X. et al. Achieving highly efficient narrowband sky-blue electroluminescence with alleviated efficiency roll-off by molecular-structure regulation and device-configuration optimization. *J. Mater. Chem. C* **10**, 15408–15415 (2022).
11. Huang, X. et al. Donor-modified multiple resonance emitters with accelerated reverse intersystem crossing towards high-efficiency and narrowband deep-blue OLEDs. *J. Mater. Chem. C* **11**, 11885–11894 (2023).
12. Kim, J. H., Chung, W. J., Kim, J. & Lee, J. Y. Concentration quenching–resistant multiresonance thermally activated delayed fluorescence emitters. *Mater. Today Energy* **21**, 100792 (2021).
13. Wang, Y. et al. A periphery cladding strategy to improve the performance of narrowband emitters, achieving deep-blue OLEDs with CIEy < 0.08 and external quantum efficiency approaching 20%. *Org. Electron.* **97**, 106275 (2021).

14. Wang, Y. et al. The selective regulation of borylation site based on one-shot electrophilic C–H borylation reaction, achieving highly efficient narrowband organic light-emitting diodes. *Chem. Eng. J.* **431**, 133221 (2022).
15. Qiu, Y. et al. Narrowing the Electroluminescence Spectra of Multiresonance Emitters for High-Performance Blue OLEDs by a Peripheral Decoration Strategy. *ACS Appl. Mater. Interfaces* **13**, 59035–59042 (2021).
16. Park, J. et al. Fine-tuned asymmetric blue multiple resonance thermally activated delayed fluorescence emitters with high efficiency and narrow emission band. *J. Mater. Chem. C* **10**, 12300–12306 (2022).
17. Park, J. et al. Asymmetric Blue Multiresonance TADF Emitters with a Narrow Emission Band. *ACS Appl. Mater. Interfaces* **13**, 45798–45805 (2021).
18. Ravindran, E. et al. Steric Rooted Multi-Resonant Thermally Activated Delayed Fluorescent Emitters for Pure Blue Organic Light Emitting Diodes with Ultralow Efficiency Roll-Off. *Adv. Funct. Mater.* **33**, 2213461 (2023).
19. Hatakeyama, T. et al. Ultrapure Blue Thermally Activated Delayed Fluorescence Molecules: Efficient HOMO–LUMO Separation by the Multiple Resonance Effect. *Adv. Mater.* **28**, 2777–2781 (2016).
20. Oda, S. et al. Carbazole-Based DABNA Analogues as Highly Efficient Thermally Activated Delayed Fluorescence Materials for Narrowband Organic Light-Emitting Diodes. *Angew. Chem. Int. Ed.* **60**, 2882–2886 (2021).
21. Cheon, H. J., Shin, Y., Park, N., Lee, J. & Kim, Y. Boron-Based Multi-Resonance TADF Emitter with Suppressed Intermolecular Interaction and Isomer Formation for Efficient Pure Blue OLEDs. *Small* **18**, 2107574 (2022).
22. Bian, J. et al. Ambipolar Self-Host Functionalization Accelerates Blue Multi-Resonance Thermally Activated Delayed Fluorescence with Internal Quantum Efficiency of 100%. *Adv. Mater.* **34**, 2110547 (2022).
23. Park, I. S., Min, H. & Yasuda, T. Ultrafast Triplet–Singlet Exciton Interconversion in Narrowband Blue Organoboron Emitters Doped with Heavy Chalcogens. *Angew. Chem. Int. Ed.* **61**, e202205684 (2022).
24. Li, Q. et al. Boron-, Sulfur- and Nitrogen-Doped Polycyclic Aromatic Hydrocarbon Multiple Resonance Emitters for Narrow-Band Blue Emission. Chem. Eur. J. 28, e202104214 (2022).

25. Hua, T. et al. Sulfone-Incorporated Multi-Resonance TADF Emitter for High-Performance Narrowband Blue OLEDs with EQE of 32%. *Adv. Funct. Mater.* **32**, 2201032 (2022).
26. Liang, X. et al. Peripheral Amplification of Multi-Resonance Induced Thermally Activated Delayed Fluorescence for Highly Efficient OLEDs. *Angew. Chem. Int. Ed.* **57**, 11316–11320 (2018).
27. Zhang, Y. et al. Multi-Resonance Induced Thermally Activated Delayed Fluorophores for Narrowband Green OLEDs. *Angew. Chem. Int. Ed.* **58**, 16912–16917 (2019).
28. Oda, S., Kawakami, B., Kawasumi, R., Okita, R. & Hatakeyama, T. Multiple Resonance Effect-Induced Sky-Blue Thermally Activated Delayed Fluorescence with a Narrow Emission Band. *Org. Lett.* **21**, 9311–9314 (2019).
29. He, Y.-H. et al. Red-shift emission and rapid up-conversion of B,N-containing electroluminescent materials *via* tuning intramolecular charge transfer. *Mater. Chem. Front.* **7**, 2454–2463 (2023).
30. Mubarok, H. et al. Triptycene-Fused Sterically Shielded Multi-Resonance TADF Emitter Enables High-Efficiency Deep Blue OLEDs with Reduced Dexter Energy Transfer. *Angew. Chem. Int. Ed.* **62**, e202306879 (2023).
31. Cai, X., Pu, Y., Li, C., Wang, Z. & Wang, Y. Multi-Resonance Building-Block-Based Electroluminescent Material: Lengthening Emission Maximum and Shortening Delayed Fluorescence Lifetime. *Angew. Chem. Int. Ed.* **62**, e202304104 (2023).
32. Jin, J. et al. Integrating Asymmetric O−B−N Unit in Multi-Resonance Thermally Activated Delayed Fluorescence Emitters towards High-Performance Deep-Blue Organic Light-Emitting Diodes. *Angew. Chem. Int. Ed.* **62**, e202218947 (2023).
33. Wang, X. et al. Mesityl-Functionalized Multi-Resonance Organoboron Delayed Fluorescent Frameworks with Wide-Range Color Tunability for Narrowband OLEDs. *Angew. Chem. Int. Ed.* **61**, e202206916 (2022).
34. Lv, X. et al. Extending the π-Skeleton of Multi-Resonance TADF Materials towards High-Efficiency Narrowband Deep-Blue Emission. *Angew. Chem. Int. Ed.* **61**, e202201588 (2022).
35. Oda, S. et al. One-Shot Synthesis of Expanded Heterohelicene Exhibiting Narrowband Thermally Activated Delayed Fluorescence. *J. Am. Chem. Soc.* **144**, 106–112 (2022).
36. Meng, G. et al. Amine-Directed Formation of B−N Bonds for BN-Fused Polycyclic Aromatic Multiple Resonance Emitters with Narrowband Emission. *Angew. Chem. Int. Ed.* **61**, e202207293 (2022).

37. Stavrou, K. et al. Emission and Absorption Tuning in TADF B,N-Doped Heptacenes: Toward Ideal-Blue Hyperfluorescent OLEDs. *Adv. Opt. Mater.* **10**, 2200688 (2022).
38. Matsui, K. et al. One-Shot Multiple Borylation toward BN-Doped Nanographenes. *J. Am. Chem. Soc.* **140**, 1195–1198 (2018).
39. Kondo, Y. et al. Narrowband deep-blue organic light-emitting diode featuring an organoboron-based emitter. *Nat. Photon.* **13**, 678–682 (2019).
40. Tanaka, H. et al. Hypsochromic Shift of Multiple-Resonance-Induced Thermally Activated Delayed Fluorescence by Oxygen Atom Incorporation. *Angew. Chem. Int. Ed.* **60**, 17910–17914 (2021).
41. Rayappa Naveen, K. *et al.* Deep blue diboron embedded multi-resonance thermally activated delayed fluorescence emitters for narrowband organic light emitting diodes. *Chem. Eng. J.* **432**, 134381 (2022).
42. Park, I. S., Yang, M., Shibata, H., Amanokura, N. & Yasuda, T. Achieving Ultimate Narrowband and Ultrapure Blue Organic Light-Emitting Diodes Based on Polycyclo-Heteraborin Multi-Resonance Delayed-Fluorescence Emitters. *Adv. Mater.* **34**, 2107951 (2022).
43. Liu, G., Sasabe, H., Kumada, K., Arai, H. & Kido, J. Nonbonding/Bonding Molecular Orbital Regulation of Nitrogen-Boron-Oxygen-embedded Blue/Green Multiresonant TADF Emitters with High Efficiency and Color Purity. *Chem. Eur. J.* **28**, e202201605 (2022).
44. Nagata, M. et al. Fused-Nonacyclic Multi-Resonance Delayed Fluorescence Emitter Based on Ladder-Thiaborin Exhibiting Narrowband Sky-Blue Emission with Accelerated Reverse Intersystem Crossing. *Angew. Chem. Int. Ed.* **60**, 20280–20285 (2021).
45. Naveen, K. R., Oh, J. H., Lee, H. S. & Kwon, J. H. Tailoring Extremely Narrow FWHM in Hypsochromic and Bathochromic Shift of Polycyclo-Heteraborin MR-TADF Materials for High-Performance OLEDs. *Angew. Chem. Int. Ed.* **62**, e202306768 (2023).
46. Yang, M., Park, I. S. & Yasuda, T. Full-Color, Narrowband, and High-Efficiency Electroluminescence from Boron and Carbazole Embedded Polycyclic Heteroaromatics. *J. Am. Chem. Soc.* **142**, 19468–19472 (2020).
