# Supplementary material for: Highly efficient pure-blue organic light-emitting diodes based on rationally designed heterocyclic phenophosphazinine-containing emitters
Source: Nat Commun. 2024 Jul 22;15:6175. doi: 10.1038/s41467-024-50370-5 (PMC11263564; doi:10.1038/s41467-024-50370-5)

checkCIF/PLATON report

Structure factors have been supplied for datablock(s) xlj

THIS REPORT IS FOR GUIDANCE ONLY. IF USED AS PART OF A REVIEW PROCEDURE FOR PUBLICATION, IT SHOULD NOT REPLACE THE EXPERTISE OF AN EXPERIENCED CRYSTALLOGRAPHIC REFEREE.

No syntax errors found. CIF dictionary Interpreting this report

Datablock: xlj

Bond precision:	C-C = 0.0020 A		Wavelength=1.54184
Cell:	a=14.4394(2)	b=13.8870(1)	c=26.8466(3)
	alpha=90	beta=102.737(1)	gamma=90
Temperature:	150 K		

	Calculated	Reported
Volume	5250.81(10)	5250.81(10)
Space group	P 21/n	P 1 21/n 1
Hall group	-P 2yn	-P 2yn
Moiety formula	C64 H61 B N3 O P	C64 H61 B N3 O P
Sum formula	C64 H61 B N3 O P	C64 H61 B N3 O P
Mr	929.94	929.93
Dx,g cm-3	1.176	1.176
Z	4	4
Mu (mm-1)	0.803	0.803
F000	1976.0	1976.0
F000’	1982.13	
h,k,lmax	17,17,33	17,17,33
Nref	10623	10387
Tmin,Tmax	0.901,0.938	0.872,1.000
Tmin’	0.901	

Correction method= # Reported T Limits: Tmin=0.872 Tmax=1.000
AbsCorr = MULTI-SCAN

Data completeness= 0.978 Theta(max)= 73.866

R(reflections)= 0.0393(9333) wR2(reflections)= 0.1020(10387)

S = 1.025 Npar= 654

The following ALERTS were generated. Each ALERT has the format
test-name_ALERT_alert-type_alert-level.
Click on the hyperlinks for more details of the test.

Alert level C

```
PLAT220_ALERT_2_C NonSolvent   Resd 1  C   Ueq(max)/Ueq(min) Range        3.5 Ratio
PLAT410_ALERT_2_C Short Intra H...H Contact  H17      ..H27      .       1.93 Ang.
                                                       x,y,z  =      1_555 Check
PLAT601_ALERT_2_C Unit Cell Contains Solvent Accessible VOIDS of .         79 Ang**3
PLAT911_ALERT_3_C Missing FCF Refl Between Thmin & STh/L=    0.600          2 Report
```

Alert level G

```
PLAT002_ALERT_2_G Number of Distance or Angle Restraints on AtSite          4 Note
PLAT142_ALERT_4_G s.u. on b - Axis Small or Missing .............     0.00010 Ang.
PLAT172_ALERT_4_G The CIF-Embedded .res File Contains DFIX Records          1 Report
PLAT173_ALERT_4_G The CIF-Embedded .res File Contains DANG Records          1 Report
PLAT187_ALERT_4_G The CIF-Embedded .res File Contains RIGU Records          2 Report
PLAT190_ALERT_3_G A Non-default RIGU Restraint Value for First Par     0.0010 Report
PLAT190_ALERT_3_G A Non-default RIGU Restraint Value for SecondPar     0.0030 Report
PLAT190_ALERT_3_G A Non-default RIGU Restraint Value for First Par     0.0010 Report
PLAT190_ALERT_3_G A Non-default RIGU Restraint Value for SecondPar     0.0030 Report
PLAT301_ALERT_3_G Main Residue  Disorder ..............(Resd  1  )          1% Note
PLAT410_ALERT_2_G Short Intra H...H Contact  H9       ..H15      .       1.93 Ang.
                                                       x,y,z  =      1_555 Check
PLAT410_ALERT_2_G Short Intra H...H Contact  H15      ..H9A      .       1.76 Ang.
                                                       x,y,z  =      1_555 Check
PLAT412_ALERT_2_G Short Intra XH3 .. XHn     H10      ..H52C     .       2.10 Ang.
                                                       x,y,z  =      1_555 Check
PLAT412_ALERT_2_G Short Intra XH3 .. XHn     H52C     ..H10A     .       2.13 Ang.
                                                       x,y,z  =      1_555 Check
PLAT860_ALERT_3_G Number of Least-Squares Restraints ............          17 Note
PLAT912_ALERT_4_G Missing # of FCF Reflections Above STh/L=  0.600        234 Note
PLAT941_ALERT_3_G Average HKL Measurement Multiplicity ..........         2.0 Low
PLAT978_ALERT_2_G Number C-C Bonds with Positive Residual Density.         20 Info
```

0 **ALERT level A** = Most likely a serious problem - resolve or explain
0 **ALERT level B** = A potentially serious problem, consider carefully
4 **ALERT level C** = Check. Ensure it is not caused by an omission or oversight
18 **ALERT level G** = General information/check it is not something unexpected

0 ALERT type 1 CIF construction/syntax error, inconsistent or missing data
9 ALERT type 2 Indicator that the structure model may be wrong or deficient
8 ALERT type 3 Indicator that the structure quality may be low
5 ALERT type 4 Improvement, methodology, query or suggestion
0 ALERT type 5 Informative message, check

It is advisable to attempt to resolve as many as possible of the alerts in all categories. Often the minor alerts point to easily fixed oversights, errors and omissions in your CIF or refinement strategy, so attention to these fine details can be worthwhile. In order to resolve some of the more serious problems it may be necessary to carry out additional measurements or structure refinements. However, the purpose of your study may justify the reported deviations and the more serious of these should normally be commented upon in the discussion or experimental section of a paper or in the "special_details" fields of the CIF. checkCIF was carefully designed to identify outliers and unusual parameters, but every test has its limitations and alerts that are not important in a particular case may appear. Conversely, the absence of alerts does not guarantee there are no aspects of the results needing attention. It is up to the individual to critically assess their own results and, if necessary, seek expert advice.

Publication of your CIF in IUCr journals

A basic structural check has been run on your CIF. These basic checks will be run on all CIFs submitted for publication in IUCr journals (*Acta Crystallographica*, *Journal of Applied Crystallography*, *Journal of Synchrotron Radiation*); however, if you intend to submit to *Acta Crystallographica Section C* or *E* or *IUCrData*, you should make sure that full publication checks are run on the final version of your CIF prior to submission.

Publication of your CIF in other journals

Please refer to the *Notes for Authors* of the relevant journal for any special instructions relating to CIF submission.

PLATON version of 28/11/2022; check.def file version of 28/11/2022

Datablock xlj - ellipsoid plot

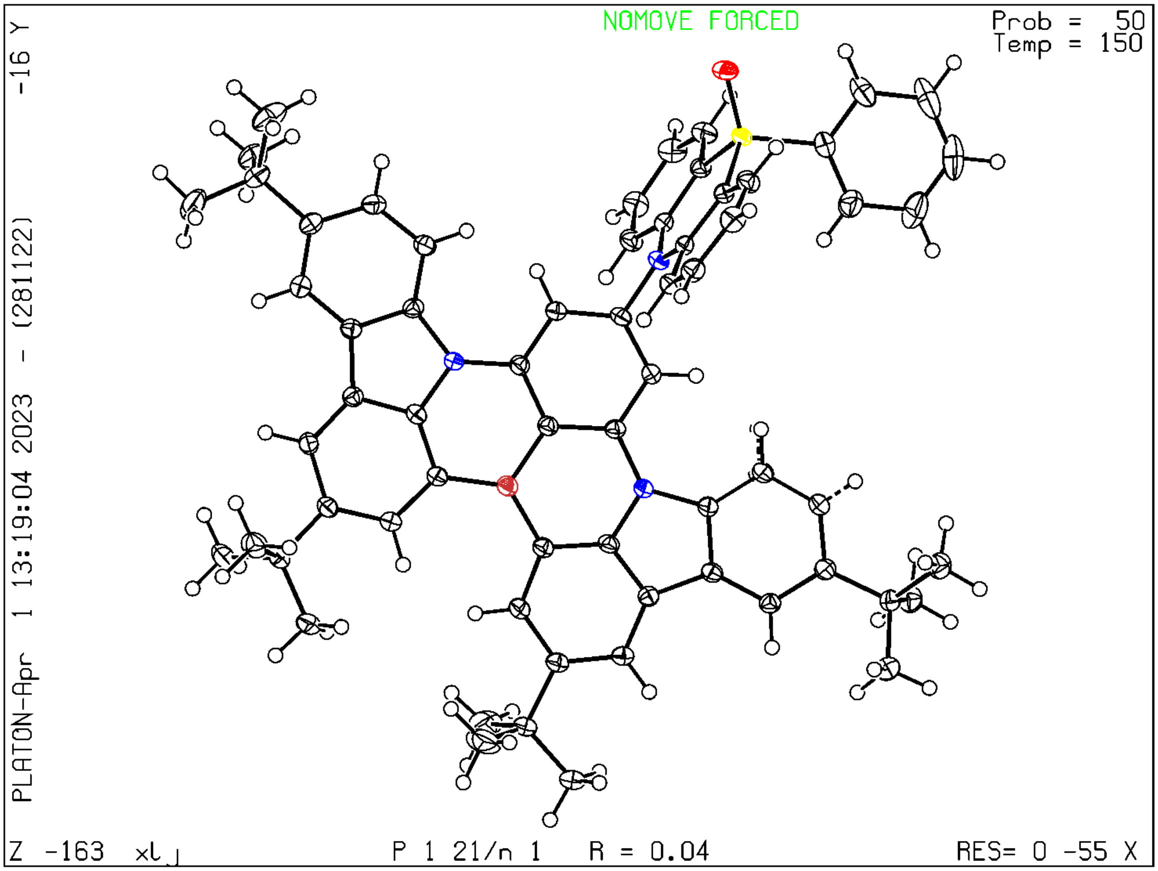

Supplement: Supplementary file 3 — Source Data [file 41467_2024_50370_MOESM3_ESM.zip › Source Data/BNCz-NPO-α_CCDC2253324.pdf]
