# Supplementary material for: Highly efficient pure-blue organic light-emitting diodes based on rationally designed heterocyclic phenophosphazinine-containing emitters
Source: Nat Commun. 2024 Jul 22;15:6175. doi: 10.1038/s41467-024-50370-5 (PMC11263564; doi:10.1038/s41467-024-50370-5)

checkCIF/PLATON report

Structure factors have been supplied for datablock(s) mpo

THIS REPORT IS FOR GUIDANCE ONLY. IF USED AS PART OF A REVIEW PROCEDURE FOR PUBLICATION, IT SHOULD NOT REPLACE THE EXPERTISE OF AN EXPERIENCED CRYSTALLOGRAPHIC REFEREE.

No syntax errors found. CIF dictionary Interpreting this report

Datablock: mpo

Bond precision: C-C = 0.0073 A Wavelength=1.54184

Cell:	a=12.6376(4)	b=15.0173(3)	c=16.3976(3)
	alpha=79.740(2)	beta=79.810(2)	gamma=72.857(2)
Temperature:	150 K		

	Calculated	Reported
Volume	2900.44(13)	2900.44(13)
Space group	P -1	P -1
Hall group	-P 1	-P 1
Moiety formula	C64 H61 B N3 O0.67 P, 0.33(O) [+ solvent]	C64 H61 B N3 O P
Sum formula	C64 H61 B N3 O P [+ solvent]	C64 H61 B N3 O P
Mr	929.94	929.93
Dx,g cm-3	1.065	1.065
Z	2	2
Mu (mm-1)	0.727	0.727
F000	988.0	988.0
F000'	991.07	
h,k,lmax	15,17,19	15,17,19
Nref	10250	10132
Tmin,Tmax	0.901,0.930	0.801,1.000
Tmin'	0.897	

Correction method= # Reported T Limits: Tmin=0.801 Tmax=1.000
AbsCorr = MULTI-SCAN

Data completeness= 0.988 Theta(max)= 66.593

R(reflections)= 0.1100(8125) wR2(reflections)= 0.3187(10132)

S = 1.108 Npar= 687

The following ALERTS were generated. Each ALERT has the format
test-name_ALERT_alert-type_alert-level.
Click on the hyperlinks for more details of the test.

Alert level C

```
PLAT082_ALERT_2_C High R1 Value ................................      0.11 Report
PLAT084_ALERT_3_C High wR2 Value (i.e. > 0.25) ..................      0.32 Report
PLAT213_ALERT_2_C Atom O1             has ADP max/min Ratio .....       3.3 prolat
PLAT220_ALERT_2_C NonSolvent   Resd 1  C   Ueq(max)/Ueq(min) Range       4.4 Ratio
PLAT222_ALERT_3_C NonSolvent Resd 1  H   Uiso(max)/Uiso(min) Range       4.3 Ratio
PLAT241_ALERT_2_C High   'MainMol' Ueq as Compared to Neighbors of       C62 Check
PLAT241_ALERT_2_C High   'MainMol' Ueq as Compared to Neighbors of       C63 Check
PLAT242_ALERT_2_C Low    'MainMol' Ueq as Compared to Neighbors of       C18 Check
PLAT242_ALERT_2_C Low    'MainMol' Ueq as Compared to Neighbors of       C28 Check
PLAT242_ALERT_2_C Low    'MainMol' Ueq as Compared to Neighbors of       C59 Check
PLAT250_ALERT_2_C Large U3/U1 Ratio for <U(i,j)> Tensor(Resd    1)       2.2 Note
PLAT303_ALERT_2_C Full Occupancy Atom H59       with # Connections      1.33 Check
PLAT340_ALERT_3_C Low Bond Precision on  C-C Bonds ..............   0.00729 Ang.
PLAT410_ALERT_2_C Short Intra H...H Contact  H33      ..H42      .      1.90 Ang.
                                                  x,y,z  =      1_555 Check
PLAT906_ALERT_3_C Large K Value in the Analysis of Variance ......     6.477 Check
PLAT906_ALERT_3_C Large K Value in the Analysis of Variance ......     2.925 Check
PLAT911_ALERT_3_C Missing FCF Refl Between Thmin & STh/L=    0.595       118 Report
               -9  9  0,  -8 10  0,   6-10  1, -12 -6  1,   1 -1  1,   3-14  3,
                2-13  3,   3-13  3,  -9  3  3,  -8 11  4,  -3 15  5,  -2 16  5,
               12 -3  6,  13 -3  6,  -1 10  6,  -2 11  6,  -1 11  6,  -2 12  6,
               -1 12  6,  -3 13  6,  -2 13  6,  -1 13  6,  -4 14  6,  -3 14  6,
               -2 14  6,  -1 14  6,  -3 15  6,  -2 15  6,  -1 15  6,  -1 16  6,
                1-14  7,   2-14  7,  -5-13  7,   0-13  7,   1-13  7, -10  4  7,
               -2 11  7,  -1 11  7,  -3 12  7,  -2 12  7,  -1 12  7,  -5 13  7,
               -4 13  7,  -3 13  7,  -2 13  7,  -1 13  7,  -4 14  7,  -3 14  7,
               -2 14  7,  -1 14  7,  -3 15  7,  -2 15  7,  -1 15  7,   0 15  7,
               -1 16  7,   0 16  7,  -5-13  8,   2-13  8,   3-13  8, -10  4  8,
               -3 12  8,  -2 12  8,  -1 12  8,  -5 13  8,  -4 13  8,  -3 13  8,
               -2 13  8,  -1 13  8,  -3 14  8,  -2 14  8,  -1 14  8,   0 14  8,
               -2 15  8,  -1 15  8,   0 15  8,   0 16  8,   1 16  8,  -5 12  9,
               -2 12  9,  -4 13  9,  -3 13  9,  -2 13  9,  -1 13  9,  -3 14  9,
               -2 14  9,  -1 14  9,   0 14  9,  -1 15  9,   0 15  9,   0 16  9,
                1 16  9,  -5 12 10,  -4 13 10,  -3 13 10,  -2 13 10,  -1 13 10,
PLAT918_ALERT_3_C Reflection(s) with I(obs) much Smaller I(calc) .         1 Check
PLAT934_ALERT_3_C Number of (Iobs-Icalc)/Sigma(W) > 10 Outliers ..         1 Check
                1-12  4,
```

Alert level G

```
PLAT007_ALERT_5_G Number of Unrefined Donor-H Atoms .............          1 Report
              H59
PLAT012_ALERT_1_G N.O.K.   _shelx_res_checksum Found in CIF ......    Please Check
PLAT042_ALERT_1_G Calc. and Reported MoietyFormula Strings  Differ    Please Check
              Calc: C64 H61 B N3 O0.67 P, 0.33(O)
```

```
              Rep.: C64 H61 B N3 O P
PLAT072_ALERT_2_G SHELXL First  Parameter in WGHT  Unusually Large       0.14 Report
PLAT083_ALERT_2_G SHELXL Second Parameter in WGHT  Unusually Large       5.08 Why ?
PLAT154_ALERT_1_G The s.u.'s on the Cell Angles are Equal ..(Note)      0.002 Degree
PLAT171_ALERT_4_G The CIF-Embedded .res File Contains EADP Records          1 Report
PLAT187_ALERT_4_G The CIF-Embedded .res File Contains RIGU Records          2 Report
PLAT190_ALERT_3_G A Non-default RIGU Restraint Value for First Par     0.0010 Report
PLAT190_ALERT_3_G A Non-default RIGU Restraint Value for First Par     0.0010 Report
PLAT190_ALERT_3_G A Non-default RIGU Restraint Value for SecondPar     0.0050 Report
PLAT230_ALERT_2_G Hirshfeld Test Diff for    P1       --O1       .        5.6 s.u.
PLAT230_ALERT_2_G Hirshfeld Test Diff for    P1       --C58      .        9.8 s.u.
PLAT301_ALERT_3_G Main Residue  Disorder .............(Resd    1)          7% Note
PLAT302_ALERT_4_G Anion/Solvent/Minor-Residue Disorder (Resd    2)        100% Note
PLAT304_ALERT_4_G Non-Integer Number of Atoms in ..... (Resd    1)     130.67 Check
PLAT304_ALERT_4_G Non-Integer Number of Atoms in ..... (Resd    2)       0.33 Check
PLAT311_ALERT_2_G Isolated Disordered Oxygen Atom (No H's ?) .....         O2 Check
PLAT412_ALERT_2_G Short Intra XH3 .. XHn     H22      ..H29C     .       2.08 Ang.
                                              x,y,z  =     1_555 Check
PLAT412_ALERT_2_G Short Intra XH3 .. XHn     H22      ..H29F     .       2.14 Ang.
                                              x,y,z  =     1_555 Check
PLAT415_ALERT_2_G Short Inter D-H..H-X       H59      ..H27E     .       2.10 Ang.
                                          -x,1-y,1-z  =     2_566 Check
PLAT432_ALERT_2_G Short Inter X...Y Contact  O2       ..C58      .       2.35 Ang.
                                              x,y,z  =     1_555 Check
PLAT432_ALERT_2_G Short Inter X...Y Contact  O2       ..C63      .       2.54 Ang.
                                              x,y,z  =     1_555 Check
PLAT432_ALERT_2_G Short Inter X...Y Contact  O2       ..C51      .       2.83 Ang.
                                              x,y,z  =     1_555 Check
PLAT432_ALERT_2_G Short Inter X...Y Contact  O2       ..C57      .       2.87 Ang.
                                              x,y,z  =     1_555 Check
PLAT606_ALERT_4_G Solvent Accessible VOID(S) in Structure ........          ! Info
PLAT720_ALERT_4_G Number of Unusual/Non-Standard Labels ..........          1 Note
              B01D
PLAT793_ALERT_4_G Model has Chirality at C59         (Centro SpGr)          R Verify
PLAT860_ALERT_3_G Number of Least-Squares Restraints .............         57 Note
PLAT868_ALERT_4_G ALERTS Due to the Use of _smtbx_masks Suppressed          ! Info
PLAT909_ALERT_3_G Percentage of I>2sig(I) Data at Theta(Max) Still        61% Note
PLAT933_ALERT_2_G Number of HKL-OMIT Records in Embedded .res File          1 Note
                3-14  3,
PLAT941_ALERT_3_G Average HKL Measurement Multiplicity ...........        2.8 Low
PLAT969_ALERT_5_G The 'Henn et al.' R-Factor-gap value ...........      6.986 Note
              Predicted wR2: Based on SigI**2  4.56 or SHELX Weight 28.77
PLAT978_ALERT_2_G Number C-C Bonds with Positive Residual Density.          1 Info
```

---

0 **ALERT level A** = Most likely a serious problem - resolve or explain
0 **ALERT level B** = A potentially serious problem, consider carefully
19 **ALERT level C** = Check. Ensure it is not caused by an omission or oversight
35 **ALERT level G** = General information/check it is not something unexpected

3 ALERT type 1 CIF construction/syntax error, inconsistent or missing data
25 ALERT type 2 Indicator that the structure model may be wrong or deficient
15 ALERT type 3 Indicator that the structure quality may be low
9 ALERT type 4 Improvement, methodology, query or suggestion
2 ALERT type 5 Informative message, check

It is advisable to attempt to resolve as many as possible of the alerts in all categories. Often the minor alerts point to easily fixed oversights, errors and omissions in your CIF or refinement strategy, so attention to these fine details can be worthwhile. In order to resolve some of the more serious problems it may be necessary to carry out additional measurements or structure refinements. However, the purpose of your study may justify the reported deviations and the more serious of these should normally be commented upon in the discussion or experimental section of a paper or in the "special_details" fields of the CIF. checkCIF was carefully designed to identify outliers and unusual parameters, but every test has its limitations and alerts that are not important in a particular case may appear. Conversely, the absence of alerts does not guarantee there are no aspects of the results needing attention. It is up to the individual to critically assess their own results and, if necessary, seek expert advice.

Publication of your CIF in IUCr journals

A basic structural check has been run on your CIF. These basic checks will be run on all CIFs submitted for publication in IUCr journals (*Acta Crystallographica*, *Journal of Applied Crystallography*, *Journal of Synchrotron Radiation*); however, if you intend to submit to *Acta Crystallographica Section C* or *E* or *IUCrData*, you should make sure that full publication checks are run on the final version of your CIF prior to submission.

Publication of your CIF in other journals

Please refer to the *Notes for Authors* of the relevant journal for any special instructions relating to CIF submission.

PLATON version of 13/05/2024; check.def file version of 04/05/2024

Datablock mpo - ellipsoid plot

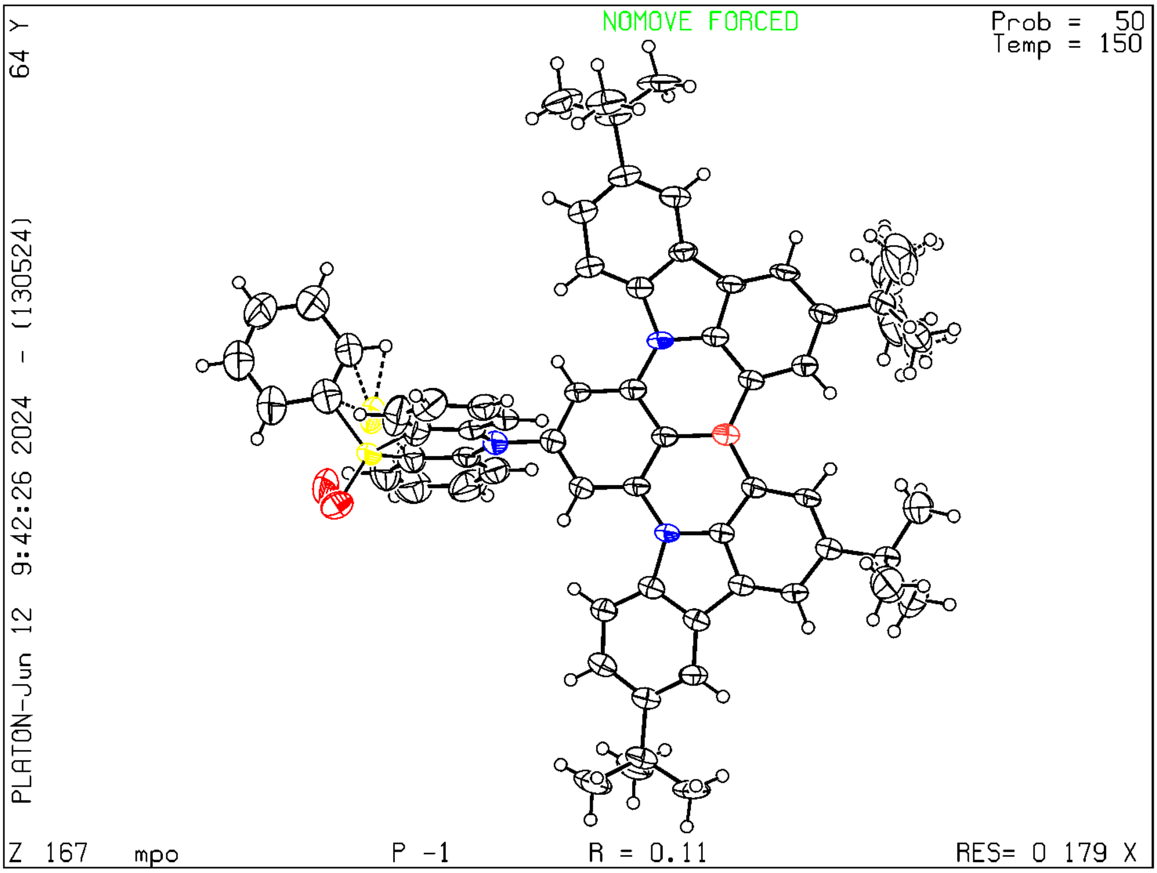

Supplement: Supplementary file 3 — Source Data [file 41467_2024_50370_MOESM3_ESM.zip › Source Data/BNCz-NPO-β_CCDC2282192.pdf]
