# Supplementary material for: Highly efficient pure-blue organic light-emitting diodes based on rationally designed heterocyclic phenophosphazinine-containing emitters
Source: Nat Commun. 2024 Jul 22;15:6175. doi: 10.1038/s41467-024-50370-5 (PMC11263564; doi:10.1038/s41467-024-50370-5)

# checkCIF/PLATON report

Structure factors have been supplied for datablock(s) nps-2

THIS REPORT IS FOR GUIDANCE ONLY. IF USED AS PART OF A REVIEW PROCEDURE FOR PUBLICATION, IT SHOULD NOT REPLACE THE EXPERTISE OF AN EXPERIENCED CRYSTALLOGRAPHIC REFEREE.

No syntax errors found. CIF dictionary Interpreting this report

# Datablock: nps-2

---

| | | | |
|---|---|---|---|
| Bond precision: | C-C = 0.0063 A | | Wavelength=1.54184 |
| Cell: | a=18.8852(1) | b=25.2745(2) | c=24.1828(2) |
| | alpha=90 | beta=105.465(1) | gamma=90 |
| Temperature: | 100 K | | |

| | Calculated | Reported |
|---|---|---|
| Volume | 11124.87(15) | 11124.86(15) |
| Space group | P 21/n | P 1 21/n 1 |
| Hall group | -P 2yn | -P 2yn |
| Moiety formula | C64 H61 B N3 P S [+ solvent] | C64 H61 B N3 P S |
| Sum formula | C64 H61 B N3 P S [+ solvent] | C64 H61 B N3 P S |
| Mr | 946.00 | 945.99 |
| Dx,g cm-3 | 1.130 | 1.130 |
| Z | 8 | 8 |
| Mu (mm-1) | 1.093 | 1.093 |
| F000 | 4016.0 | 4016.0 |
| F000' | 4030.55 | |
| h,k,lmax | 22,30,28 | 22,30,28 |
| Nref | 19657 | 19630 |
| Tmin,Tmax | 0.843,0.887 | 0.901,1.000 |
| Tmin' | 0.840 | |

Correction method= # Reported T Limits: Tmin=0.901 Tmax=1.000
AbsCorr = MULTI-SCAN

Data completeness= 0.999 Theta(max)= 66.599

```
R(reflections)= 0.1004( 18003)                               wR2(reflections)=
                                                             0.2461( 19630)
S = 1.091                 Npar= 1364
```

---

The following ALERTS were generated. Each ALERT has the format
**test-name_ALERT_alert-type_alert-level**.
Click on the hyperlinks for more details of the test.

---

## Alert level C

```
PLAT213_ALERT_2_C Atom C102            has ADP max/min Ratio .....        3.4 prolat
PLAT220_ALERT_2_C NonSolvent   Resd 1  C   Ueq(max)/Ueq(min) Range        4.6 Ratio
PLAT220_ALERT_2_C NonSolvent   Resd 2  C   Ueq(max)/Ueq(min) Range        3.7 Ratio
PLAT222_ALERT_3_C NonSolvent Resd 1  H   Uiso(max)/Uiso(min) Range        5.7 Ratio
PLAT242_ALERT_2_C Low    'MainMol' Ueq as Compared to Neighbors of        C81 Check
PLAT332_ALERT_2_C Large Phenyl C-C Range     C122      -C127     .       0.18 Ang.
PLAT340_ALERT_3_C Low Bond Precision on  C-C Bonds ..............     0.00627 Ang.
PLAT410_ALERT_2_C Short Intra H...H Contact  H75      ..H108     .       1.90 Ang.
                                                  x,y,z  =     1_555 Check
PLAT410_ALERT_2_C Short Intra H...H Contact  H28      ..H40      .       1.91 Ang.
                                                  x,y,z  =     1_555 Check
PLAT906_ALERT_3_C Large K Value in the Analysis of Variance ......      6.358 Check
PLAT906_ALERT_3_C Large K Value in the Analysis of Variance ......      2.231 Check
PLAT911_ALERT_3_C Missing FCF Refl Between Thmin & STh/L=    0.595         27 Report
                0  2  0,   1  1  1,   0  2  1,  -1  2  2,  -1  3  3, -16 21  6,
                1  8  7, -16 21  7, -20  5  8, -21  0 13,  -6 24 14, -21  0 15,
              -21  1 15,  -6 24 15, -21  1 16, -21  0 17,   3  0 17, -21  1 17,
                2 23 17,   2 21 18,   2 22 18,   2 21 19,   6 10 22, -13  0 23,
              -12  0 26,  -5  5 28,  -5  6 28,
PLAT918_ALERT_3_C Reflection(s) with I(obs) much Smaller I(calc) .          1 Check
PLAT971_ALERT_2_C Check Calcd Resid. Dens.  0.37Ang From C128            1.60 eA-3
PLAT972_ALERT_2_C Check Calcd Resid. Dens.  0.06Ang From C60            -2.12 eA-3
PLAT972_ALERT_2_C Check Calcd Resid. Dens.  0.12Ang From C1B            -1.52 eA-3
PLAT977_ALERT_2_C Check Negative Difference Density on H60       .      -0.33 eA-3
PLAT977_ALERT_2_C Check Negative Difference Density on H1A       .      -0.39 eA-3
```

---

## Alert level G

```
PLAT002_ALERT_2_G Number of Distance or Angle Restraints on AtSite         29 Note
PLAT003_ALERT_2_G Number of Uiso or U(i,j) Restrained non-H Atoms          15 Report
PLAT012_ALERT_1_G N.O.K.   _shelx_res_checksum Found in CIF ......     Please Check
PLAT083_ALERT_2_G SHELXL Second Parameter in WGHT  Unusually Large      48.94 Why ?
PLAT142_ALERT_4_G s.u. on b - Axis Small or Missing .............     0.00020 Ang.
PLAT143_ALERT_4_G s.u. on c - Axis Small or Missing .............     0.00020 Ang.
PLAT171_ALERT_4_G The CIF-Embedded .res File Contains EADP Records          6 Report
PLAT172_ALERT_4_G The CIF-Embedded .res File Contains DFIX Records          1 Report
PLAT176_ALERT_4_G The CIF-Embedded .res File Contains SADI Records         22 Report
PLAT177_ALERT_4_G The CIF-Embedded .res File Contains DELU Records          2 Report
PLAT178_ALERT_4_G The CIF-Embedded .res File Contains SIMU Records          5 Report
PLAT186_ALERT_4_G The CIF-Embedded .res File Contains ISOR Records          4 Report
PLAT187_ALERT_4_G The CIF-Embedded .res File Contains RIGU Records         12 Report
PLAT188_ALERT_3_G A Non-default SIMU Restraint Value has been used     0.0006 Report
PLAT188_ALERT_3_G A Non-default SIMU Restraint Value has been used     0.0006 Report
PLAT188_ALERT_3_G A Non-default SIMU Restraint Value has been used     0.0005 Report
PLAT188_ALERT_3_G A Non-default SIMU Restraint Value has been used     0.0030 Report
```

```
PLAT188_ALERT_3_G A Non-default SIMU Restraint Value has been used     0.0006 Report
PLAT190_ALERT_3_G A Non-default RIGU Restraint Value for First Par     0.0010 Report
PLAT190_ALERT_3_G A Non-default RIGU Restraint Value for SecondPar     0.0050 Report
PLAT190_ALERT_3_G A Non-default RIGU Restraint Value for First Par     0.0010 Report
PLAT190_ALERT_3_G A Non-default RIGU Restraint Value for First Par     0.0010 Report
PLAT190_ALERT_3_G A Non-default RIGU Restraint Value for SecondPar     0.0050 Report
PLAT190_ALERT_3_G A Non-default RIGU Restraint Value for First Par     0.0010 Report
PLAT190_ALERT_3_G A Non-default RIGU Restraint Value for SecondPar     0.0050 Report
PLAT190_ALERT_3_G A Non-default RIGU Restraint Value for First Par     0.0010 Report
PLAT190_ALERT_3_G A Non-default RIGU Restraint Value for SecondPar     0.0020 Report
PLAT190_ALERT_3_G A Non-default RIGU Restraint Value for First Par     0.0010 Report
PLAT190_ALERT_3_G A Non-default RIGU Restraint Value for First Par     0.0010 Report
PLAT190_ALERT_3_G A Non-default RIGU Restraint Value for SecondPar     0.0050 Report
PLAT190_ALERT_3_G A Non-default RIGU Restraint Value for First Par     0.0010 Report
PLAT190_ALERT_3_G A Non-default RIGU Restraint Value for SecondPar     0.0050 Report
PLAT190_ALERT_3_G A Non-default RIGU Restraint Value for First Par     0.0010 Report
PLAT190_ALERT_3_G A Non-default RIGU Restraint Value for First Par     0.0010 Report
PLAT190_ALERT_3_G A Non-default RIGU Restraint Value for SecondPar     0.0050 Report
PLAT190_ALERT_3_G A Non-default RIGU Restraint Value for First Par     0.0010 Report
PLAT190_ALERT_3_G A Non-default RIGU Restraint Value for SecondPar     0.0050 Report
PLAT190_ALERT_3_G A Non-default RIGU Restraint Value for First Par     0.0010 Report
PLAT190_ALERT_3_G A Non-default RIGU Restraint Value for SecondPar     0.0060 Report
PLAT191_ALERT_3_G A Non-default SADI Restraint Value has been used     0.0300 Report
PLAT191_ALERT_3_G A Non-default SADI Restraint Value has been used     0.0400 Report
PLAT191_ALERT_3_G A Non-default SADI Restraint Value has been used     0.0400 Report
PLAT191_ALERT_3_G A Non-default SADI Restraint Value has been used     0.0400 Report
PLAT191_ALERT_3_G A Non-default SADI Restraint Value has been used     0.0400 Report
PLAT191_ALERT_3_G A Non-default SADI Restraint Value has been used     0.0400 Report
PLAT191_ALERT_3_G A Non-default SADI Restraint Value has been used     0.0400 Report
PLAT191_ALERT_3_G A Non-default SADI Restraint Value has been used     0.0400 Report
PLAT191_ALERT_3_G A Non-default SADI Restraint Value has been used     0.0400 Report
PLAT192_ALERT_3_G A Non-default DELU Restraint Value for First Par     0.0006 Report
PLAT192_ALERT_3_G A Non-default DELU Restraint Value for SecondPar     0.0006 Report
PLAT300_ALERT_4_G Atom Site Occupancy of C83       Constrained at        0.5 Check
PLAT300_ALERT_4_G Atom Site Occupancy of C106      Constrained at        0.5 Check
PLAT300_ALERT_4_G Atom Site Occupancy of H83       Constrained at        0.5 Check
PLAT300_ALERT_4_G Atom Site Occupancy of H106      Constrained at        0.5 Check
PLAT300_ALERT_4_G Atom Site Occupancy of C38       Constrained at        0.5 Check
PLAT300_ALERT_4_G Atom Site Occupancy of C38A      Constrained at        0.5 Check
PLAT300_ALERT_4_G Atom Site Occupancy of H38       Constrained at        0.5 Check
PLAT300_ALERT_4_G Atom Site Occupancy of H38A      Constrained at        0.5 Check
PLAT301_ALERT_3_G Main Residue  Disorder ..............(Resd    1)        20% Note
PLAT301_ALERT_3_G Main Residue  Disorder ..............(Resd    2)         1% Note
PLAT410_ALERT_2_G Short Intra H...H Contact  H83      ..H96      .       1.77 Ang.
                                                    x,y,z  =     1_555 Check
PLAT410_ALERT_2_G Short Intra H...H Contact  H96      ..H106     .       1.89 Ang.
                                                    x,y,z  =     1_555 Check
PLAT410_ALERT_2_G Short Intra H...H Contact  H12      ..H38      .       1.88 Ang.
                                                    x,y,z  =     1_555 Check
PLAT410_ALERT_2_G Short Intra H...H Contact  H12      ..H38A     .       1.85 Ang.
                                                    x,y,z  =     1_555 Check
PLAT411_ALERT_2_G Short Inter H...H Contact  H60      ..H1E      .       2.14 Ang.
                                      1/2+x,3/2-y,1/2+z  =     4_676 Check
PLAT412_ALERT_2_G Short Intra XH3 .. XHn     H10D     ..H97      .       2.12 Ang.
                                                    x,y,z  =     1_555 Check
PLAT412_ALERT_2_G Short Intra XH3 .. XHn     H93      ..H86F     .       2.10 Ang.
                                                    x,y,z  =     1_555 Check
```

```
PLAT412_ALERT_2_G Short Intra XH3 .. XHn     H97       ..H1FB      .       2.03 Ang.
                                                    x,y,z  =      1_555 Check
PLAT413_ALERT_2_G Short Inter XH3 .. XHn     H119      ..H84F      .       1.89 Ang.
                                       3/2-x,1/2+y,1/2-z  =      2_655 Check
PLAT432_ALERT_2_G Short Inter X...Y Contact  S3        ..C78       .       3.25 Ang.
                                              1-x,1-y,1-z  =      3_666 Check
PLAT432_ALERT_2_G Short Inter X...Y Contact  C62       ..C129      .       3.12 Ang.
                                      -1/2+x,3/2-y,1/2+z  =      4_576 Check
PLAT432_ALERT_2_G Short Inter X...Y Contact  C63       ..C129      .       2.95 Ang.
                                      -1/2+x,3/2-y,1/2+z  =      4_576 Check
PLAT606_ALERT_4_G Solvent Accessible VOID(S) in Structure ........          ! Info
PLAT720_ALERT_4_G Number of Unusual/Non-Standard Labels ..........         11 Note
              C02R    H02A    H02B    H02C    C00M    H1FA    H1FB    H1FC
              H1GA    H1GB    H1GC
PLAT721_ALERT_1_G Bond    Calc     0.97000, Rep     0.98920 Dev...       0.02 Ang.
              C1F      -H1FA           1_555   1_555 ........     #  315 Check
PLAT721_ALERT_1_G Bond    Calc     0.98000, Rep     0.99030 Dev...       0.01 Ang.
              C1F      -H1FB           1_555   1_555 ........     #  316 Check
PLAT721_ALERT_1_G Bond    Calc     0.99000, Rep     1.05110 Dev...       0.06 Ang.
              C1G      -H1GA           1_555   1_555 ........     #  318 Check
PLAT721_ALERT_1_G Bond    Calc     0.98000, Rep     1.03340 Dev...       0.05 Ang.
              C1G      -H1GB           1_555   1_555 ........     #  319 Check
PLAT721_ALERT_1_G Bond    Calc     0.97000, Rep     1.05350 Dev...       0.08 Ang.
              C1G      -H1GC           1_555   1_555 ........     #  320 Check
PLAT722_ALERT_1_G Angle   Calc      120.00, Rep      118.80 Dev...       1.20 Degree
              C37     -C38A    -H38A       1_555  1_555  1_555     #  546 Check
PLAT722_ALERT_1_G Angle   Calc      110.00, Rep      108.50 Dev...       1.50 Degree
              C101    -C129    -H12A       1_555  1_555  1_555     #  566 Check
PLAT722_ALERT_1_G Angle   Calc      109.00, Rep      110.60 Dev...       1.60 Degree
              C101    -C129    -H12B       1_555  1_555  1_555     #  567 Check
PLAT722_ALERT_1_G Angle   Calc      109.00, Rep      111.20 Dev...       2.20 Degree
              C101    -C1F     -H1FB       1_555  1_555  1_555     #  573 Check
PLAT722_ALERT_1_G Angle   Calc      109.00, Rep      110.10 Dev...       1.10 Degree
              C101    -C1F     -H1FC       1_555  1_555  1_555     #  574 Check
PLAT722_ALERT_1_G Angle   Calc      110.00, Rep      108.60 Dev...       1.40 Degree
              H1FA    -C1F     -H1FB       1_555  1_555  1_555     #  575 Check
PLAT722_ALERT_1_G Angle   Calc      110.00, Rep      108.50 Dev...       1.50 Degree
              H1FA    -C1F     -H1FC       1_555  1_555  1_555     #  576 Check
PLAT722_ALERT_1_G Angle   Calc      109.00, Rep      116.90 Dev...       7.90 Degree
              C101    -C1G     -H1GA       1_555  1_555  1_555     #  578 Check
PLAT722_ALERT_1_G Angle   Calc      109.00, Rep      113.50 Dev...       4.50 Degree
              C101    -C1G     -H1GB       1_555  1_555  1_555     #  579 Check
PLAT722_ALERT_1_G Angle   Calc      110.00, Rep      117.30 Dev...       7.30 Degree
              C101    -C1G     -H1GC       1_555  1_555  1_555     #  580 Check
PLAT722_ALERT_1_G Angle   Calc      109.00, Rep      103.40 Dev...       5.60 Degree
              H1GA    -C1G     -H1GB       1_555  1_555  1_555     #  581 Check
PLAT722_ALERT_1_G Angle   Calc      109.00, Rep      101.90 Dev...       7.10 Degree
              H1GA    -C1G     -H1GC       1_555  1_555  1_555     #  582 Check
PLAT722_ALERT_1_G Angle   Calc      110.00, Rep      102.00 Dev...       8.00 Degree
              H1GB    -C1G     -H1GC       1_555  1_555  1_555     #  583 Check
PLAT860_ALERT_3_G Number of Least-Squares Restraints ............       1533 Note
PLAT868_ALERT_4_G ALERTS Due to the Use of _smtbx_masks Suppressed          ! Info
PLAT909_ALERT_3_G Percentage of I>2sig(I) Data at Theta(Max) Still        84% Note
PLAT933_ALERT_2_G Number of HKL-OMIT Records in Embedded .res File          1 Note
                0  2  1,
PLAT941_ALERT_3_G Average HKL Measurement Multiplicity ..........        3.1 Low
PLAT969_ALERT_5_G The 'Henn et al.' R-Factor-gap value ..........      5.416 Note
```

```
             Predicted wR2: Based on SigI**2  4.54 or SHELX Weight 22.56
PLAT978_ALERT_2_G Number C-C Bonds with Positive Residual Density.         3 Info
```

---

```
  0 ALERT level A = Most likely a serious problem - resolve or explain
  0 ALERT level B = A potentially serious problem, consider carefully
 18 ALERT level C = Check. Ensure it is not caused by an omission or oversight
 99 ALERT level G = General information/check it is not something unexpected

 19 ALERT type 1 CIF construction/syntax error, inconsistent or missing data
 29 ALERT type 2 Indicator that the structure model may be wrong or deficient
 48 ALERT type 3 Indicator that the structure quality may be low
 20 ALERT type 4 Improvement, methodology, query or suggestion
  1 ALERT type 5 Informative message, check
```

---

It is advisable to attempt to resolve as many as possible of the alerts in all categories. Often the minor alerts point to easily fixed oversights, errors and omissions in your CIF or refinement strategy, so attention to these fine details can be worthwhile. In order to resolve some of the more serious problems it may be necessary to carry out additional measurements or structure refinements. However, the purpose of your study may justify the reported deviations and the more serious of these should normally be commented upon in the discussion or experimental section of a paper or in the "special_details" fields of the CIF. checkCIF was carefully designed to identify outliers and unusual parameters, but every test has its limitations and alerts that are not important in a particular case may appear. Conversely, the absence of alerts does not guarantee there are no aspects of the results needing attention. It is up to the individual to critically assess their own results and, if necessary, seek expert advice.

**Publication of your CIF in IUCr journals**

A basic structural check has been run on your CIF. These basic checks will be run on all CIFs submitted for publication in IUCr journals (*Acta Crystallographica*, *Journal of Applied Crystallography*, *Journal of Synchrotron Radiation*); however, if you intend to submit to *Acta Crystallographica Section C* or *E* or *IUCrData*, you should make sure that full publication checks are run on the final version of your CIF prior to submission.

**Publication of your CIF in other journals**

Please refer to the *Notes for Authors* of the relevant journal for any special instructions relating to CIF submission.

---

**PLATON version of 13/05/2024; check.def file version of 04/05/2024**

**Datablock nps-2** - ellipsoid plot

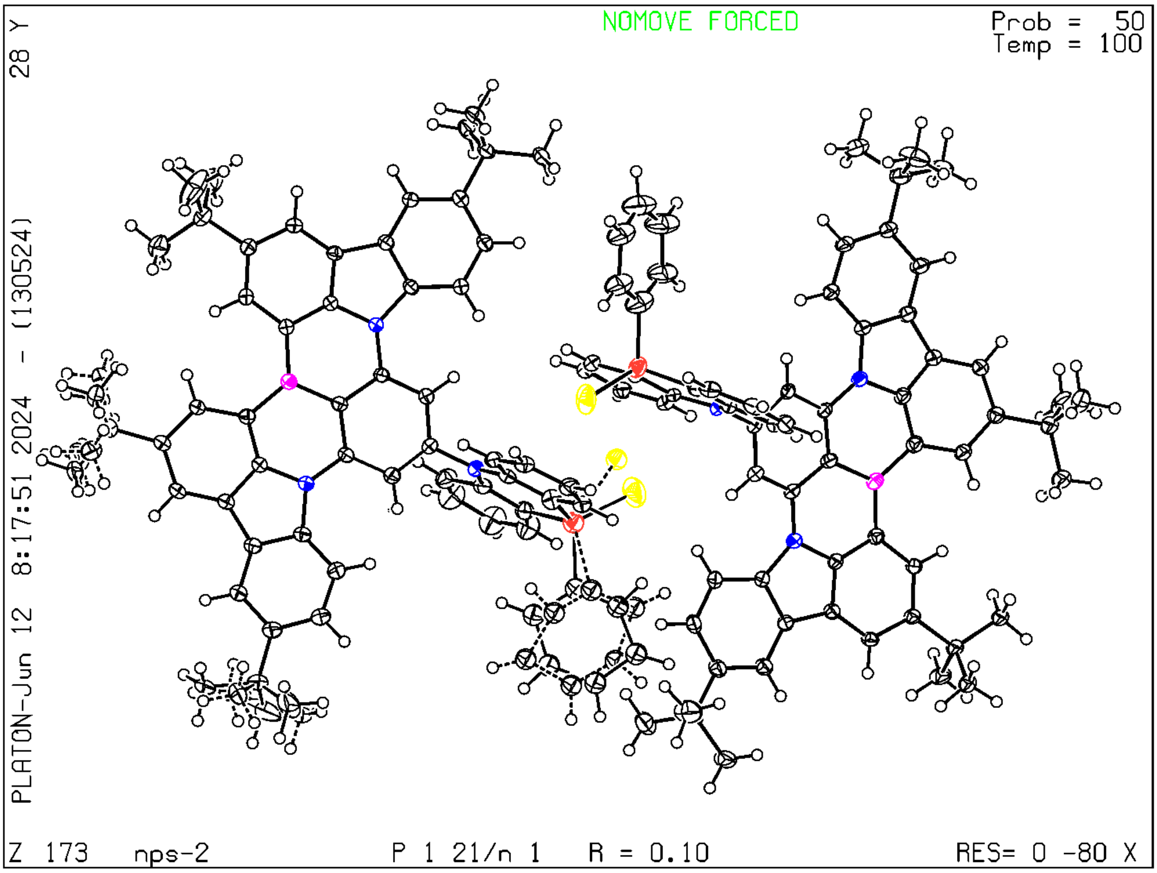

Supplement: Supplementary file 3 — Source Data [file 41467_2024_50370_MOESM3_ESM.zip › Source Data/BNCz-NPS-α_CCDC2285218.pdf]
