# Supplementary material for: Highly efficient pure-blue organic light-emitting diodes based on rationally designed heterocyclic phenophosphazinine-containing emitters
Source: Nat Commun. 2024 Jul 22;15:6175. doi: 10.1038/s41467-024-50370-5 (PMC11263564; doi:10.1038/s41467-024-50370-5)

checkCIF/PLATON report

Structure factors have been supplied for datablock(s) xlj

THIS REPORT IS FOR GUIDANCE ONLY. IF USED AS PART OF A REVIEW PROCEDURE FOR PUBLICATION, IT SHOULD NOT REPLACE THE EXPERTISE OF AN EXPERIENCED CRYSTALLOGRAPHIC REFEREE.

No syntax errors found. CIF dictionary Interpreting this report

Datablock: xlj

```
Bond precision:   C-C = 0.0065 A          Wavelength=1.54184

Cell:             a=17.4998(9)     b=21.3090(11)      c=17.6514(10)
                  alpha=90         beta=97.258(5)     gamma=90
Temperature:      170 K
```

	Calculated	Reported
Volume	6529.5(6)	6529.5(6)
Space group	C c	C 1 c 1
Hall group	C -2yc	C -2yc
Moiety formula	C64 H61 B N3 P S [+ solvent]	C64 H61 B N3 P S
Sum formula	C64 H61 B N3 P S [+ solvent]	C64 H61 B N3 P S
Mr	946.00	945.99
Dx,g cm-3	0.962	0.962
Z	4	4
Mu (mm-1)	0.931	0.931
F000	2008.0	2008.0
F000'	2015.28	
h,k,lmax	20,25,21	20,25,21
Nref	11540[5776]	7819
Tmin,Tmax	0.870,0.903	0.868,1.000
Tmin'	0.870	

```
Correction method= # Reported T Limits: Tmin=0.868 Tmax=1.000
AbsCorr = MULTI-SCAN

Data completeness= 1.35/0.68        Theta(max)= 66.594
```

R(reflections)= 0.1062(5976) wR2(reflections)= 0.3026(7819)

S = 1.091 Npar= 602

The following ALERTS were generated. Each ALERT has the format
test-name_ALERT_alert-type_alert-level.
Click on the hyperlinks for more details of the test.

Alert level C

```
STRVA01_ALERT_4_C           Flack test results are ambiguous.
           From the CIF: _refine_ls_abs_structure_Flack    0.460
           From the CIF: _refine_ls_abs_structure_Flack_su    0.020
PLAT082_ALERT_2_C High R1 Value ................................        0.11 Report
PLAT084_ALERT_3_C High wR2 Value (i.e. > 0.25) ..................        0.30 Report
PLAT213_ALERT_2_C Atom C33             has ADP max/min Ratio .....         3.2 prolat
PLAT213_ALERT_2_C Atom C35             has ADP max/min Ratio .....         3.1 prolat
PLAT213_ALERT_2_C Atom C36             has ADP max/min Ratio .....         3.2 prolat
PLAT220_ALERT_2_C NonSolvent   Resd 1  C   Ueq(max)/Ueq(min) Range         3.9 Ratio
PLAT220_ALERT_2_C NonSolvent   Resd 1  P   Ueq(max)/Ueq(min) Range         3.7 Ratio
PLAT241_ALERT_2_C High   'MainMol' Ueq as Compared to Neighbors of         C12 Check
PLAT241_ALERT_2_C High   'MainMol' Ueq as Compared to Neighbors of         C57 Check
PLAT242_ALERT_2_C Low    'MainMol' Ueq as Compared to Neighbors of         C13 Check
PLAT242_ALERT_2_C Low    'MainMol' Ueq as Compared to Neighbors of         C34 Check
PLAT242_ALERT_2_C Low    'MainMol' Ueq as Compared to Neighbors of         C55 Check
PLAT303_ALERT_2_C Full Occupancy Atom H61       with # Connections        1.12 Check
PLAT334_ALERT_2_C Small <C-C> Benzene Dist.   C24      -C29       .       1.37 Ang.
PLAT340_ALERT_3_C Low Bond Precision on  C-C Bonds ..............    0.00653 Ang.
PLAT360_ALERT_2_C Short  C(sp3)-C(sp3) Bond  C13      - C17       .       1.37 Ang.
PLAT360_ALERT_2_C Short  C(sp3)-C(sp3) Bond  C34      - C36       .       1.43 Ang.
PLAT361_ALERT_2_C Long   C(sp3)-C(sp3) Bond  C34      - C35       .       1.68 Ang.
PLAT420_ALERT_2_C D-H Bond Without Acceptor  S1       --H61       .     Please Check
PLAT911_ALERT_3_C Missing FCF Refl Between Thmin & STh/L=    0.595           2 Report
                5  1 19,  -1   7 20,
PLAT987_ALERT_1_C The Flack x is >> 0 -  Do a BASF/TWIN Refinement     Please Check
```

Alert level G

```
PLAT002_ALERT_2_G Number of Distance or Angle Restraints on AtSite           2 Note
PLAT003_ALERT_2_G Number of Uiso or U(i,j) Restrained non-H Atoms           21 Report
PLAT007_ALERT_5_G Number of Unrefined Donor-H Atoms .............            1 Report
              H61
PLAT012_ALERT_1_G N.O.K.   _shelx_res_checksum Found in CIF ......     Please Check
PLAT072_ALERT_2_G SHELXL First  Parameter in WGHT  Unusually Large        0.16 Report
PLAT083_ALERT_2_G SHELXL Second Parameter in WGHT  Unusually Large       19.26 Why ?
PLAT111_ALERT_2_G ADDSYM Detects New (Pseudo) Centre of Symmetry .          89 %Fit
PLAT113_ALERT_2_G ADDSYM Suggests Possible Pseudo/New  Space Group        C2/c Check
              WARNING: Disordered Atoms Excluded from Analysis
              Check Model Parameter Symmetry for Reflection Data Support
PLAT171_ALERT_4_G The CIF-Embedded .res File Contains EADP Records           6 Report
PLAT173_ALERT_4_G The CIF-Embedded .res File Contains DANG Records           1 Report
PLAT177_ALERT_4_G The CIF-Embedded .res File Contains DELU Records           8 Report
PLAT178_ALERT_4_G The CIF-Embedded .res File Contains SIMU Records           8 Report
PLAT186_ALERT_4_G The CIF-Embedded .res File Contains ISOR Records           8 Report
PLAT187_ALERT_4_G The CIF-Embedded .res File Contains RIGU Records           7 Report
```

```
PLAT188_ALERT_3_G A Non-default SIMU Restraint Value has been used     0.0020 Report
PLAT188_ALERT_3_G A Non-default SIMU Restraint Value has been used     0.0030 Report
PLAT188_ALERT_3_G A Non-default SIMU Restraint Value has been used     0.0020 Report
PLAT188_ALERT_3_G A Non-default SIMU Restraint Value has been used     0.0020 Report
PLAT188_ALERT_3_G A Non-default SIMU Restraint Value has been used     0.0000 Report
PLAT188_ALERT_3_G A Non-default SIMU Restraint Value has been used     0.0020 Report
PLAT188_ALERT_3_G A Non-default SIMU Restraint Value has been used     0.0020 Report
PLAT188_ALERT_3_G A Non-default SIMU Restraint Value has been used     0.0004 Report
PLAT190_ALERT_3_G A Non-default RIGU Restraint Value for First Par     0.0010 Report
PLAT190_ALERT_3_G A Non-default RIGU Restraint Value for SecondPar     0.0030 Report
PLAT190_ALERT_3_G A Non-default RIGU Restraint Value for First Par     0.0010 Report
PLAT190_ALERT_3_G A Non-default RIGU Restraint Value for SecondPar     0.0020 Report
PLAT190_ALERT_3_G A Non-default RIGU Restraint Value for First Par     0.0010 Report
PLAT190_ALERT_3_G A Non-default RIGU Restraint Value for SecondPar     0.0030 Report
PLAT190_ALERT_3_G A Non-default RIGU Restraint Value for First Par     0.0010 Report
PLAT190_ALERT_3_G A Non-default RIGU Restraint Value for SecondPar     0.0020 Report
PLAT190_ALERT_3_G A Non-default RIGU Restraint Value for First Par     0.0010 Report
PLAT190_ALERT_3_G A Non-default RIGU Restraint Value for SecondPar     0.0030 Report
PLAT190_ALERT_3_G A Non-default RIGU Restraint Value for First Par     0.0010 Report
PLAT190_ALERT_3_G A Non-default RIGU Restraint Value for SecondPar     0.0060 Report
PLAT190_ALERT_3_G A Non-default RIGU Restraint Value for First Par     0.0010 Report
PLAT190_ALERT_3_G A Non-default RIGU Restraint Value for SecondPar     0.0060 Report
PLAT192_ALERT_3_G A Non-default DELU Restraint Value for First Par     0.0005 Report
PLAT192_ALERT_3_G A Non-default DELU Restraint Value for SecondPar     0.0005 Report
PLAT192_ALERT_3_G A Non-default DELU Restraint Value for First Par     0.0004 Report
PLAT192_ALERT_3_G A Non-default DELU Restraint Value for SecondPar     0.0004 Report
PLAT230_ALERT_2_G Hirshfeld Test Diff for    P2       --C53      .      429.3 s.u.
PLAT230_ALERT_2_G Hirshfeld Test Diff for    P2       --C54      .       33.9 s.u.
PLAT230_ALERT_2_G Hirshfeld Test Diff for    P2       --C60      .      438.5 s.u.
PLAT230_ALERT_2_G Hirshfeld Test Diff for    P2       --C65      .      423.8 s.u.
PLAT230_ALERT_2_G Hirshfeld Test Diff for    C4       --C7A      .      294.9 s.u.
PLAT230_ALERT_2_G Hirshfeld Test Diff for    C7       --C8       .        6.8 s.u.
PLAT230_ALERT_2_G Hirshfeld Test Diff for    C8       --C7A      .        7.0 s.u.
PLAT230_ALERT_2_G Hirshfeld Test Diff for    C12      --C7A      .      102.0 s.u.
PLAT230_ALERT_2_G Hirshfeld Test Diff for    C29      --C28A     .        8.0 s.u.
PLAT301_ALERT_3_G Main Residue  Disorder .............(Resd    1)         7% Note
PLAT335_ALERT_2_G Check Large C6 Ring C-C Range C1       -C6            0.15 Ang.
PLAT335_ALERT_2_G Check Large C6 Ring C-C Range C1       -C6A           0.19 Ang.
PLAT335_ALERT_2_G Check Large C6 Ring C-C Range C48      -C53           0.18 Ang.
PLAT410_ALERT_2_G Short Intra H...H Contact  H6       ..H42      .       1.84 Ang.
                                                      x,y,z  =      1_555 Check
PLAT410_ALERT_2_G Short Intra H...H Contact  H28      ..H40      .       1.79 Ang.
                                                      x,y,z  =      1_555 Check
PLAT410_ALERT_2_G Short Intra H...H Contact  H40      ..H28A     .       1.52 Ang.
                                                      x,y,z  =      1_555 Check
PLAT413_ALERT_2_G Short Inter XH3 .. XHn     H15A     ..H28A     .       2.10 Ang.
                                             1/2+x,-1/2+y,z  =      3_545 Check
PLAT606_ALERT_4_G Solvent Accessible VOID(S) in Structure ........          ! Info
PLAT773_ALERT_2_G Check long C-C Bond in CIF: C27      --C28A           1.80 Ang.
PLAT792_ALERT_1_G Model has Chirality at P1          (Polar  SpGr)          S Verify
PLAT860_ALERT_3_G Number of Least-Squares Restraints ............         854 Note
PLAT868_ALERT_4_G ALERTS Due to the Use of _smtbx_masks Suppressed          ! Info
PLAT909_ALERT_3_G Percentage of I>2sig(I) Data at Theta(Max) Still        55% Note
PLAT910_ALERT_3_G Missing # of FCF Reflection(s) Below Theta(Min).          1 Note
                1  1  0,
PLAT915_ALERT_3_G No Flack x Check Done: Low Friedel Pair Coverage         36 %
PLAT933_ALERT_2_G Number of HKL-OMIT Records in Embedded .res File          1 Note
```

```
                1  1  0,
PLAT941_ALERT_3_G Average HKL Measurement Multiplicity ..........        3.5 Low
PLAT955_ALERT_1_G Reported (CIF) and Actual (FCF) Lmax Differ by .         1 Units
PLAT969_ALERT_5_G The 'Henn et al.' R-Factor-gap value ..........      5.025 Note
             Predicted wR2: Based on SigI**2  6.02 or SHELX Weight 27.73
PLAT978_ALERT_2_G Number C-C Bonds with Positive Residual Density.         0 Info
```

```
   0 ALERT level A = Most likely a serious problem - resolve or explain
   0 ALERT level B = A potentially serious problem, consider carefully
  22 ALERT level C = Check. Ensure it is not caused by an omission or oversight
  70 ALERT level G = General information/check it is not something unexpected

   4 ALERT type 1 CIF construction/syntax error, inconsistent or missing data
  42 ALERT type 2 Indicator that the structure model may be wrong or deficient
  35 ALERT type 3 Indicator that the structure quality may be low
   9 ALERT type 4 Improvement, methodology, query or suggestion
   2 ALERT type 5 Informative message, check
```

It is advisable to attempt to resolve as many as possible of the alerts in all categories. Often the minor alerts point to easily fixed oversights, errors and omissions in your CIF or refinement strategy, so attention to these fine details can be worthwhile. In order to resolve some of the more serious problems it may be necessary to carry out additional measurements or structure refinements. However, the purpose of your study may justify the reported deviations and the more serious of these should normally be commented upon in the discussion or experimental section of a paper or in the "special_details" fields of the CIF. checkCIF was carefully designed to identify outliers and unusual parameters, but every test has its limitations and alerts that are not important in a particular case may appear. Conversely, the absence of alerts does not guarantee there are no aspects of the results needing attention. It is up to the individual to critically assess their own results and, if necessary, seek expert advice.

Publication of your CIF in IUCr journals

A basic structural check has been run on your CIF. These basic checks will be run on all CIFs submitted for publication in IUCr journals (*Acta Crystallographica*, *Journal of Applied Crystallography*, *Journal of Synchrotron Radiation*); however, if you intend to submit to *Acta Crystallographica Section C* or *E* or *IUCrData*, you should make sure that full publication checks are run on the final version of your CIF prior to submission.

Publication of your CIF in other journals

Please refer to the *Notes for Authors* of the relevant journal for any special instructions relating to CIF submission.

PLATON version of 13/05/2024; check.def file version of 04/05/2024

Datablock xlj - ellipsoid plot

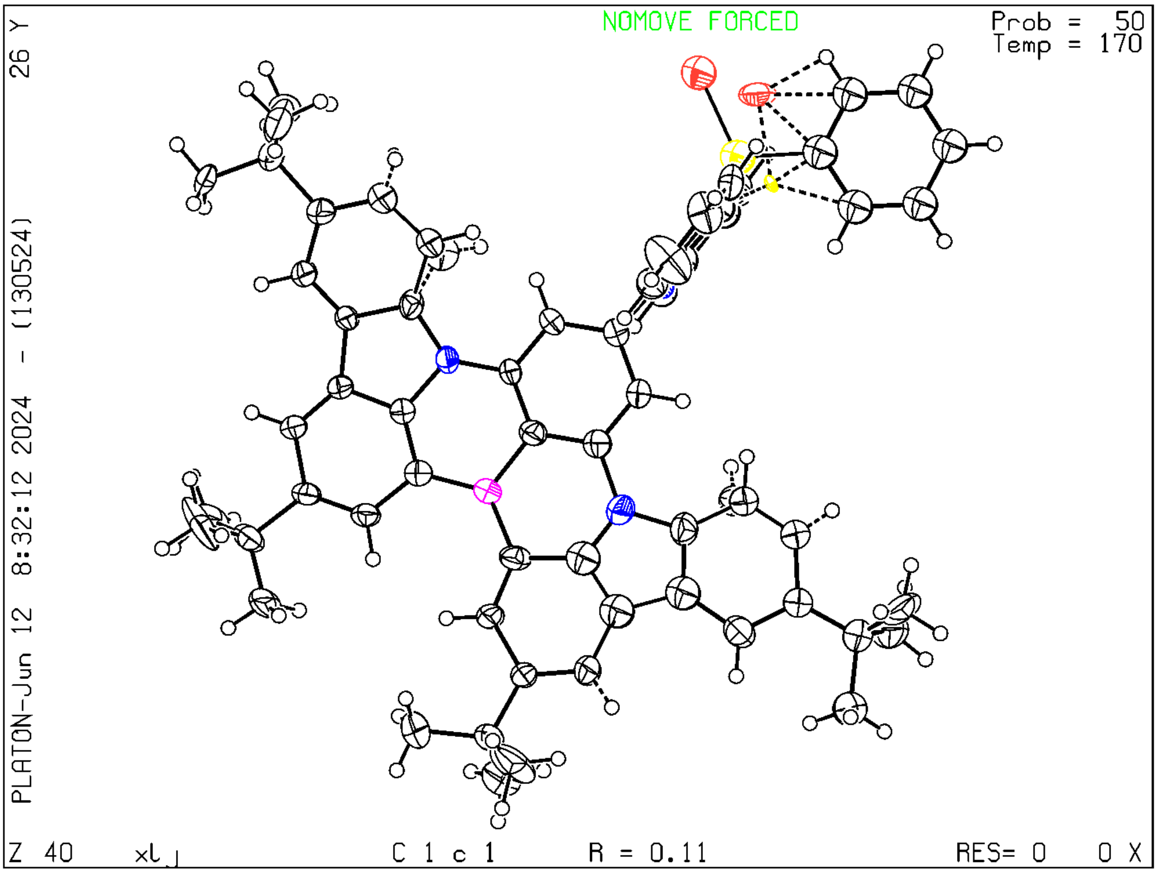

Supplement: Supplementary file 3 — Source Data [file 41467_2024_50370_MOESM3_ESM.zip › Source Data/BNCz-NPS-β_CCDC2247937.pdf]
